# Disorder-specific alterations of transient oscillatory dynamics during sleep across cortical and subcortical networks

**DOI:** 10.1038/s41598-025-33669-1

**Published:** 2026-01-20

**Authors:** Nazanin Biabani, Katarina Ilic, Adam Birdseye, Olga Ivanenko, Sean Higgins, Jan Rosenzweig, Zoran Cvetkovic, Alexander D. Nesbitt, Carlotta Mutti, Liborio Parrino, Sharon Naismith, Panagis Drakatos, Karl Morten, David O’Regan, Peter J. Goadsby, Robert Leech, Ivana Rosenzweig

**Affiliations:** 1 Sleep and Brain Plasticity Centre, Department of Neuroimaging, Institute of Psychiatry, Psychology and Neuroscience (IoPPN), King’s College London, London, UK; 2 Department of Neuroimaging, BRAIN Centre, Institute of Psychiatry Psychology and Neuroscience, King’s College London, London, UK; 3 Sleep Disorders Centre, Guy’s and St Thomas’ NHS Foundation Trust, London, UK; 4 Department of Engineering, King’s College London, London, UK; 5 Department of Neurology, Guy’s and St Thomas’ NHS Foundation Trust, London, UK; 6 Sleep Disorders Centre, Department of Medicine and Surgery, Mario Giovanni Terzano Interdepartmental Centre for Sleep Medicine, Parma University Hospital, University of Parma, Parma, Italy; 7 Healthy Brain Ageing Program, The Brain and Mind Centre, University of Sydney, Sydney, Australia; 8 School of Basic and Medical Biosciences, Faculty of Life Science and Medicine, King’s College London, London, UK; 9 Nuffield Department of Women’s and Reproductive Health, University of Oxford, Oxford, UK; 10 NIHR-Wellcome Trust King’s Clinical Research Facility, King’s College London, London, UK; 11 Centre for Neuroimaging Science, King’s College London, London, UK; 12 Sleep and Brain Plasticity Centre, Department of Neuroimaging, Institute of Psychiatry, Psychology and Neuroscience, De Crespigny Park, Box 089, London, SE5 8AF UK

**Keywords:** Sleep microstructure, Slow oscillations, Sleep spindles, time-frequency analysis, Sleep disorders, Neurology, Neuroscience

## Abstract

Transient sleep oscillations reflect the dynamic coordination of cortical and subcortical circuits, modulated by slow oscillatory activity. However, the disorder-specific signatures of these events across neurological, pain, and sleep disorders remain poorly characterized. In this exploratory study, we analyzed transient oscillatory dynamics in 99 individuals, including healthy controls and patients with narcolepsy type 1, non-REM parasomnia, idiopathic REM sleep behavior disorder, and fibromyalgia syndrome. Using slow oscillatory referenced time-frequency peak histograms, we applied principal and independent component analysis to uncover spectral and phase-coupling patterns across non-REM and REM stages. We identified reproducible, trait-like oscillatory structures in controls and disorder-specific deviations in patient groups, particularly during NREM sleep. Specifically, patients with narcolepsy type 1 and non-REM parasomnia exhibited altered fast sigma coupling and phase dispersion, while idiopathic REM sleep behavior disorder patients showed reduced fast sigma density and diminished phase synchrony, despite retention of spindle-like spectral structure. In internal cross-validation, slow oscillatory-power features supported robust group-level discrimination in select EEG derivations; however, broader validation in independent samples is required. These findings highlight distinctive, stage-specific microstructural alterations in sleep and pain pathologies and support the future potential of time-frequency peak analysis as a non-invasive tool for phenotyping thalamocortical and subcortical circuit function.

**Supplementary Information:**

The online version contains supplementary material available at 10.1038/s41598-025-33669-1.

## Introduction

Sleep is increasingly recognised not as a sequence of discrete stages, but as a continuum of evolving neural dynamics spanning multiple spatial and temporal scales^1,2^. Central to this organization are transient oscillatory events, brief, frequency-specific patterns that index thalamocortical synchrony and modulate cognition, arousal, and memory consolidation^3–5^. Canonical sleep scoring, based on amplitude and morphology thresholds, often fails to capture the fine-grained structure and timing of these oscillations, particularly their modulation by cortical slow oscillations (SOs)^6–8^. Emerging frameworks now conceptualise oscillations not as discrete events but as field-like fluctuations shaped by local SO phase and power^9–13^. Among these, the time-frequency peak (TF-peak) method enables fine-resolution mapping of oscillatory distributions across frequency, time, and state^14^. This approach has revealed stable, individual-specific traits in healthy sleep, yet its applicability to clinical populations remains unexplored.

Recent methodological advances have begun to reconceptualize sleep oscillations as field-like, rather than event-like, entities. In particular, the time-frequency peak (TF-peak) framework introduced by Stokes and colleagues^14^ allows for the unbiased characterization of transient events across the 4–25 Hz range, parametrized by their coupling to SO power and phase. In this framework, sigma-band TF-peaks (10–16 Hz) correspond closely to spindle-like thalamocortical bursts, and their distribution across SO-power bins and SO-phase angles captures when, relative to cortical down- and up-states, these spindle-like events tend to occur. For example, fast sigma activity (12–15 Hz) typically clusters around the SO trough, whereas slow sigma (10–12 Hz) can preferentially emerge on the up-slope towards the positive peak^14,15^. This approach has demonstrated that transient oscillatory distributions exhibit high trait stability, topographical specificity, and inter-individual variability, suggesting that they may serve as robust electrophysiological phenotypes. Complementary modelling by Chen and colleagues^15^ further revealed that spindle timing is more strongly governed by intrinsic history-dependent processes than by external SO phase alone, challenging assumptions of exogenous pacemaking and inviting a systems-level perspective on oscillatory coordination.

**Fig. 1:**
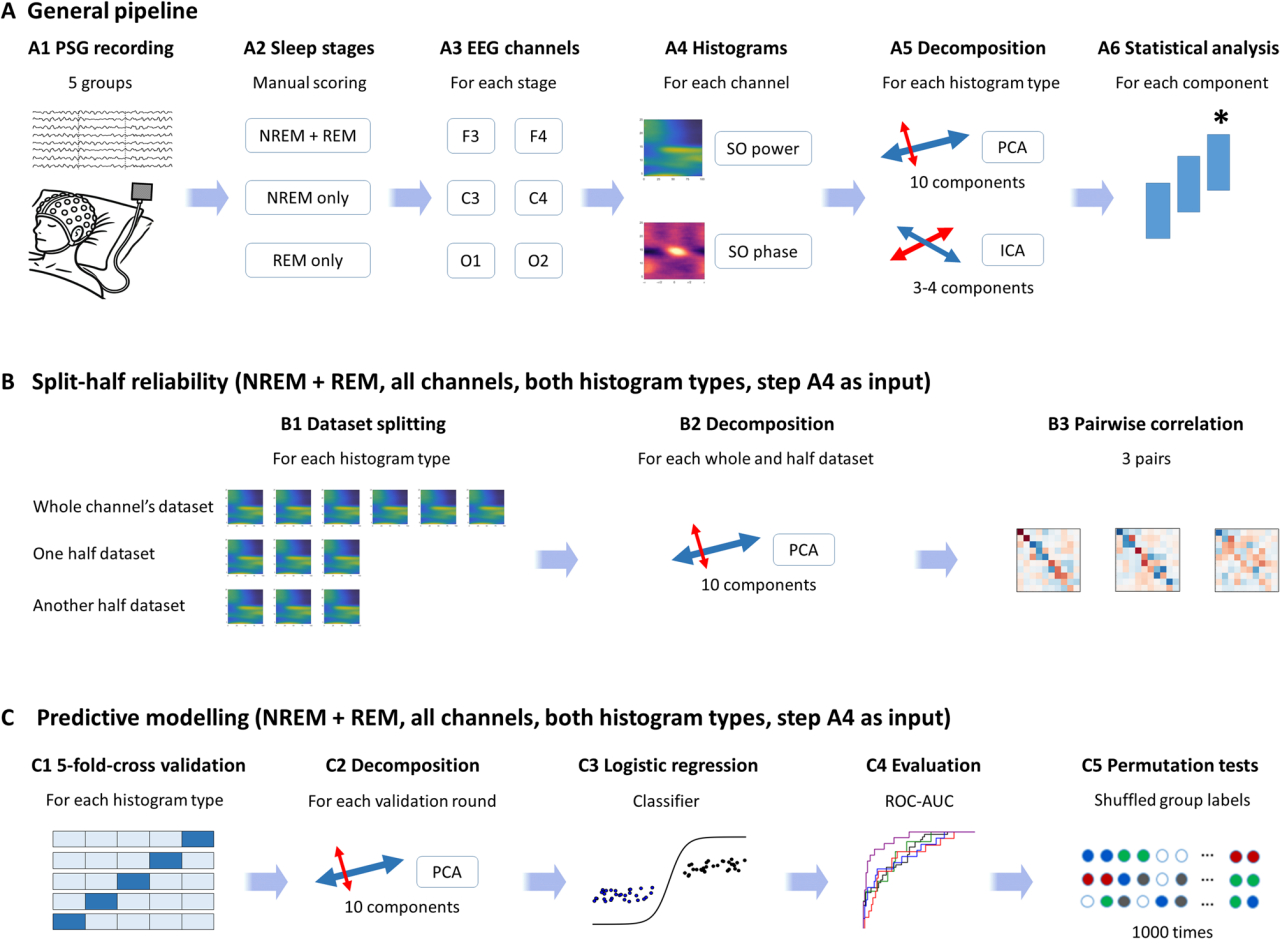
Overview of the data processing and analysis pipeline. (**A**) For each participant, six EEG channels (F3, F4, C3, C4, O1, O2) were extracted from overnight PSG recordings. Slow-oscillation (SO)–referenced TF-peak histograms were computed separately for SO-power and SO-phase in NREM + REM, NREM-only, and REM-only datasets. These histograms were then decomposed using principal component analysis (PCA) and independent component analysis (ICA), and the resulting component scores were submitted to group-wise statistical testing. (**B**) Split-half reliability analyses (NREM + REM, all channels, both histogram types) assessed the stability of PCA eigenvectors by comparing components derived from whole-channel and half-split datasets. (**C**) Predictive modelling (NREM + REM, all channels, both histogram types) used PCA projection scores as features in one-vs-all logistic regression classifiers with five-fold cross-validation; performance was quantified by the area under the receiver operating characteristic curve (ROC-AUC) and evaluated against null distributions obtained from 1,000 label-shuffle permutation tests. *AUC* area under the ROC curve, *EEG* electroencephalography, *ICA* independent component analysis, *NREM* non-rapid eye movement sleep, *PCA* principal component analysis, *PSG* polysomnography, *REM* rapid eye movement sleep, *ROC* receiver operating characteristic, *SO* slow oscillation, *TF-peak* time–frequency peak.

These findings raise critical questions about the nature and organization of transient oscillations in sleep disorders. To what extent do alterations in SO-coupled dynamics reflect disorder-specific pathophysiological processes? Are deviations stage-specific, or do they generalize across NREM and REM architecture? Can TF-peak distributions provide sensitive and specific markers of neural circuit dysfunction? To address these questions, we examined transient oscillatory dynamics across five well-characterized groups: healthy individuals and patients with fibromyalgia syndrome (FM)^16^, narcolepsy type 1 (NT1), non-REM parasomnia (NREMP), and idiopathic REM sleep behavior disorder (iRBD)^17^. These disorders were selected to span distinct etiological mechanisms, ranging from sensory amplification (FM)^16^ and neuromodulatory disruption (NT1), to cortical hyperexcitability (NREMP)^18^ and progressive neurodegeneration (iRBD)^19^. Each represents a unique perturbation of the broader thalamocortical and cortico-subcortical systems subserving sleep. An overview of the methods is presented in Fig. 1.

Source-level and magnetoencephalographic work has begun to link these oscillatory patterns to specific cortical and subcortical generators. For example, combined EEG–MEG and modelling studies have localised fast sigma activity to fronto-central thalamocortical networks, with slow sigma and low-frequency rhythms showing more anterior and posterior biases, respectively^13,20,21^. Related work by Brancaccio and colleagues and others has emphasised that slow oscillations and associated spindles emerge from coordinated interactions between cortical layers and thalamic nuclei, rather than from a single pacemaker structure^20^. Situating TF-peak structure within this framework allows us to interpret deviations in SO-power and SO-phase coupling in terms of altered thalamocortical and cortico-subcortical communication, rather than purely as changes in surface EEG morphology. Using histograms of TF-peak occurrences parametrized by SO power and SO phase, we decomposed oscillatory structure via principal and independent component analysis (PCA and ICA), enabling dimensionality reduction and cross-group comparison. Importantly, we analyzed NREM and REM stages both separately and jointly, permitting the identification of stage-specific alterations and their relation to underlying network states. We hypothesized that NT1 would show reduced coupling of fast sigma activity to high SO-power states and altered SO-phase preference, reflecting impaired spindle synchronization secondary to orexin deficiency. NREMP was expected to exhibit increased TF-peak density and broader phase dispersion, indicative of impaired inhibitory gating during transitions between cortical states. In iRBD, we anticipated changes in phase alignment, consistent with early brainstem-cortical desynchronization. FM, by contrast, was hypothesized to show modest, spatially restricted deviations reflecting localized thalamocortical dysregulation.

By situating transient oscillatory events within a continuous, SO-referenced framework, this exploratory study sought to advance the neurophysiological characterization of sleep, establish mechanistic signatures of disease-specific dysfunction, and lay the groundwork for non-invasive biomarker development grounded in sleep microstructure.

## Results

Demographic and sleep architecture data for all five cohorts are summarized in Supplementary Information (SI), Table S1.

### SO-power histograms

#### Group-level distributions

**Fig. 2:**
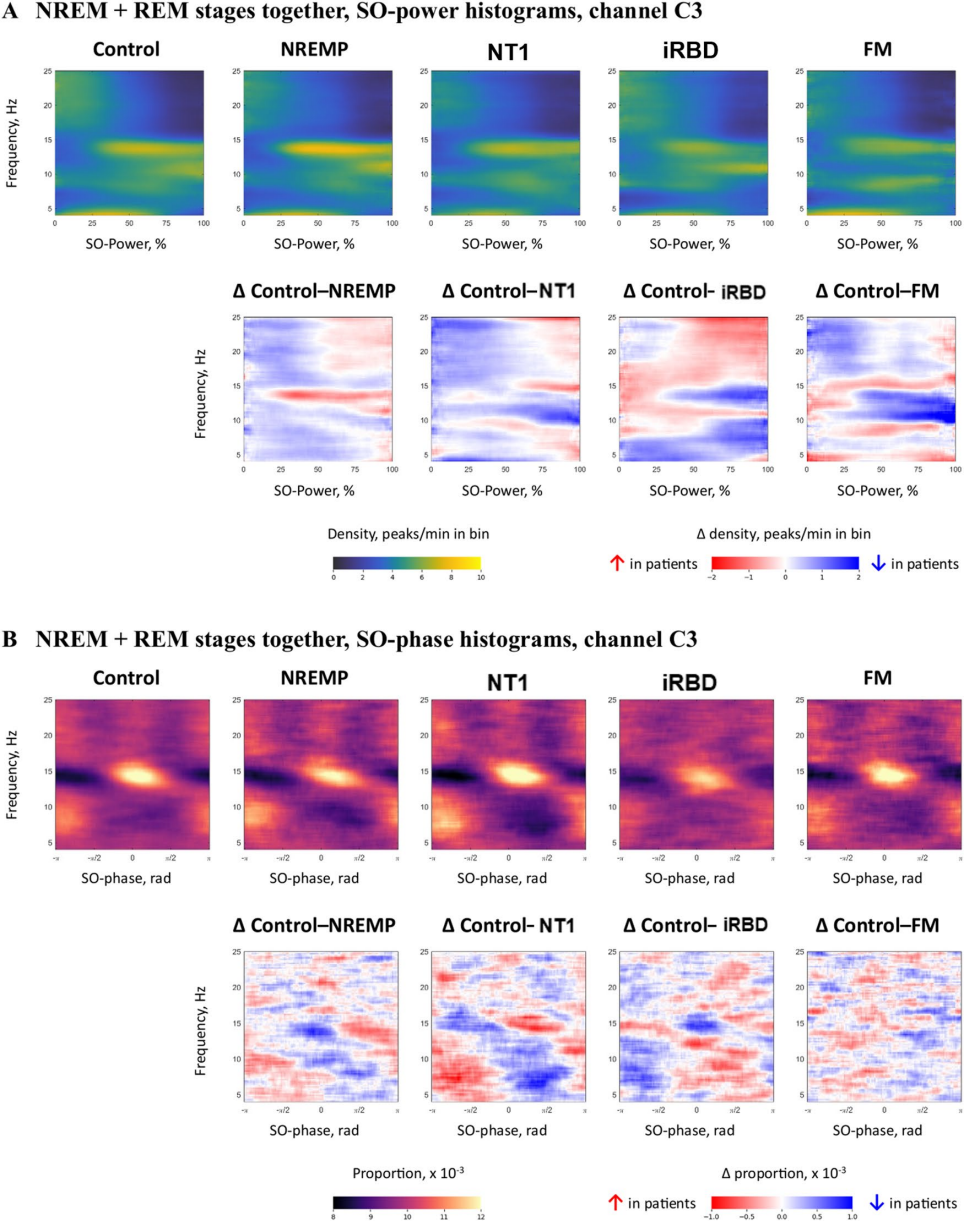
NREM + REM SO-power and SO-phase histograms in channel C3. (**A**) Group-averaged SO-power histograms (top row) and corresponding difference maps (bottom row) for controls and each patient group (NREMP, NT1, iRBD, FM). Histograms display TF-peak density as a function of peak frequency (y-axis) and SO-power bin (x-axis). Colour indicates absolute density (peaks/min in bin; left colour bar), while difference maps (Δ Control–patient) show the change in density relative to controls (right colour bar; Δ density, peaks/min in bin). Positive values (red) denote higher TF-peak density in patients; negative values (blue) denote lower density. (**B**) By visual inspection, group-averaged SO-phase histograms (top row) and corresponding difference maps (bottom row) for the same channel and groups. Histograms display the proportion of TF-peaks as a function of peak frequency (y-axis) and SO phase (x-axis, −π to π). Colour indicates absolute proportion (×10⁻³; left colour bar), and difference maps show the change in proportion relative to controls (right colour bar; Δ proportion ×10⁻³). Positive values (red) indicate a greater proportion of TF-peaks in patients at the corresponding frequency–phase combination; negative values (blue) indicate a lower proportion. *FM* fibromyalgia syndrome, *Hz*, hertz, *min* minutes,*NREM* non-rapid eye movement sleep, *NREMP* non-REM parasomnia, *NT1* narcolepsy type 1, *PCA* principal component analysis, *iRBD* idiopathic/isolated rapid eye movement sleep behaviour disorder, *rad* radians, *REM* rapid eye movement sleep,*SO* slow oscillation.

By visual inspection, in combined NREM + REM sleep stages, healthy controls showed distinct frequency–power coupling: fast sigma (12–15 Hz) peaks aligned with high SO power (≥ 50%), while slower sigma (10–12 Hz) TF peaks were associated with higher SO power (≥ 75%) (Fig. 2). Each SO-power histogram can be read as a two-dimensional map where the x-axis indexes peak frequency, the y-axis bins SO power into quartiles, and colour indicates TF-peak density (peaks per minute) in each frequency–power bin.

Clinical groups exhibited disorder-specific deviations. In NT1, both slow and fast sigma-band TF-peak density appeared reduced at high SO-power levels, particularly in frontal and central derivations. In iRBD, fast sigma density was reduced at high SO power, whereas slow sigma activity showed a more mixed pattern, with relative increases at slightly lower SO-power bins in some channels. NREMP showed alterations in both slow and fast sigma activity during high SO power, though the direction and magnitude of these effects varied by frequency band and derivation. FM displayed a combination of elevated theta and low-alpha (4–10 Hz) activity and relative reductions in sigma-band density at high SO-power levels. Difference maps highlighted these shifts: NT1 and iRBD showed decreased density in 12–15 Hz/high SO-power bins, while FM showed increases in low-frequency bins alongside modest sigma-band reductions. Please refer to Fig. 2 and SI Fig. S1–S4.

#### Component decomposition and statistical comparisons

**Fig. 3:**
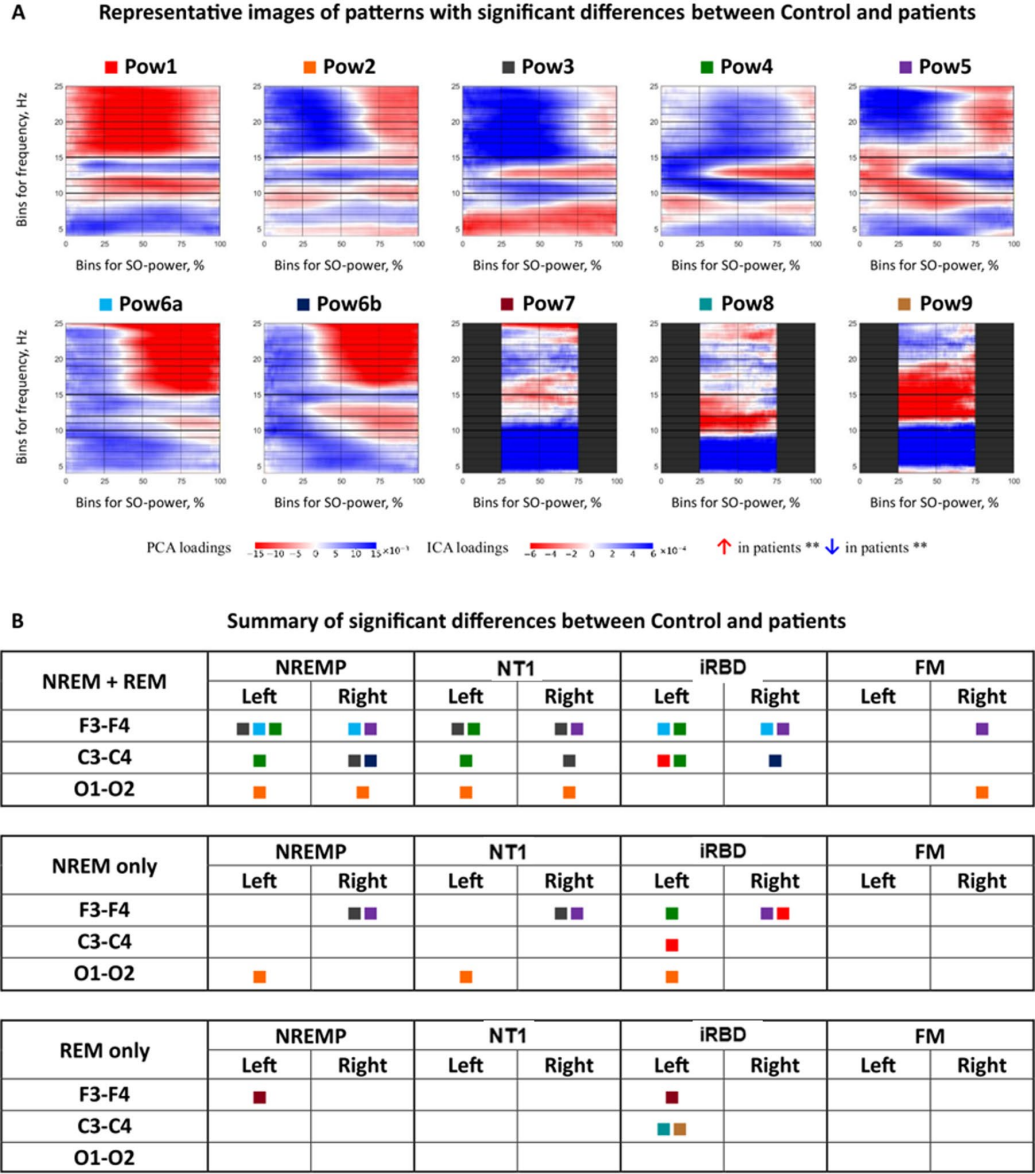
SO-power component patterns and pattern-level summary of group differences. (**A**) Spectral–SO-power profiles of the nine canonical SO-power patterns (Pow1–Pow9), shown as heatmaps. Each map illustrates the characteristic frequency–SO-power coupling structure derived from PCA and ICA loadings across all participants and channels. Red and blue values indicate bins in which higher or lower TF-peak density, respectively, is associated with higher component scores; patterns have been oriented so that increased values correspond to higher TF-peak density in patients in the majority of significant contrasts. (**B**) Pattern-level summary of significant group differences. Coloured squares indicate channels and hemispheres in which at least one PCA or ICA component belonging to the corresponding SO-power pattern showed a significant difference (*p* ≤ 0.05, Bonferroni-adjusted Mann–Whitney tests) between controls and the indicated patient group (NREMP, NT1, iRBD, FM) for each sleep stage (NREM + REM sleep, NREM-only sleep, REM-only sleep); empty cells indicate no significant effect. This panel provides an illustrative overview of the spatial and stage-specific distribution of SO-power patterns with significant group differences. A complete quantitative summary of the number of significant SO-power components per group and stage is given in Supplementary Figure S18 and Supplementary Tables S2–S16; sociodemographic and polysomnographic characteristics are reported in Supplementary Table S1. *CI* confidence interval, *FM* fibromyalgia, *Hz* hertz, *ICA* independent component analysis, *NREM* non-rapid eye movement sleep,*NREMP* non-REM parasomnia, *NT1* narcolepsy type 1, *PCA* principal component analysis, *Pow1–Pow9* SO-power patterns 1–9, *iRBD* idiopathic/isolated rapid eye movement sleep behaviour disorder, *REM* rapid eye movement sleep, *SO* slow oscillation,*%* percent.

For SO power histograms, PCA and ICA resulted in nine recurring component patterns (Pow1–Pow9; Fig. 3 and SI Fig. S5) capturing distinct frequency-power motifs. Kruskal–Wallis tests revealed significant group differences across multiple components. For instance, in RBD-specific pattern Pow1, there was a decrease in fast, and an increase in slow sigma band density. In Pow3, fast sigma was elevated in NT1 and NREMP. Finally, in Pow6a, high-frequency bins > 15 Hz/50%+ SO-power had higher values in RBD and NREMP versus Control.

Effect sizes (η²) ranged from 0.12 to 0.35, strongest in frontal (F4) and central (C3) leads. Detailed post hoc results are in SI, Tables S2–S16.

#### Stage-specific patterns

Disorder-specific alterations were primarily driven by NREM sleep (Fig. 3, SI Fig. S5). NT1 and NREMP retained significant deviations in Pow3 during NREM + REM sleep and NREM-only sleep analyses. FM did not significantly differ from controls in NREM. In REM, only iRBD and NREMP showed significant deviations; specifically, both disorders were noted, for example, for a decrease in frequency bands below 9 Hz. These findings underscore NREM sleep as the dominant stage for sleep-spindle related microstructural alterations.

#### Reliability of component structure

To evaluate the stability of extracted spectral features, we performed a split-half reliability analysis across all EEG derivations (SI, Fig. S12). Principal component structures derived from the SO-power histograms demonstrated strong internal consistency. Specifically, Spearman correlation coefficients between components extracted from each subset and those from the full dataset exceeded |0.75| for the first five components in most of the cases. These results indicate that the identified frequency–power patterns reflect reproducible signal structure rather than noise or sample-specific variance, supporting their utility in comparative analyses and downstream modelling.

#### Exploratory classification analyses

**Fig. 4:**
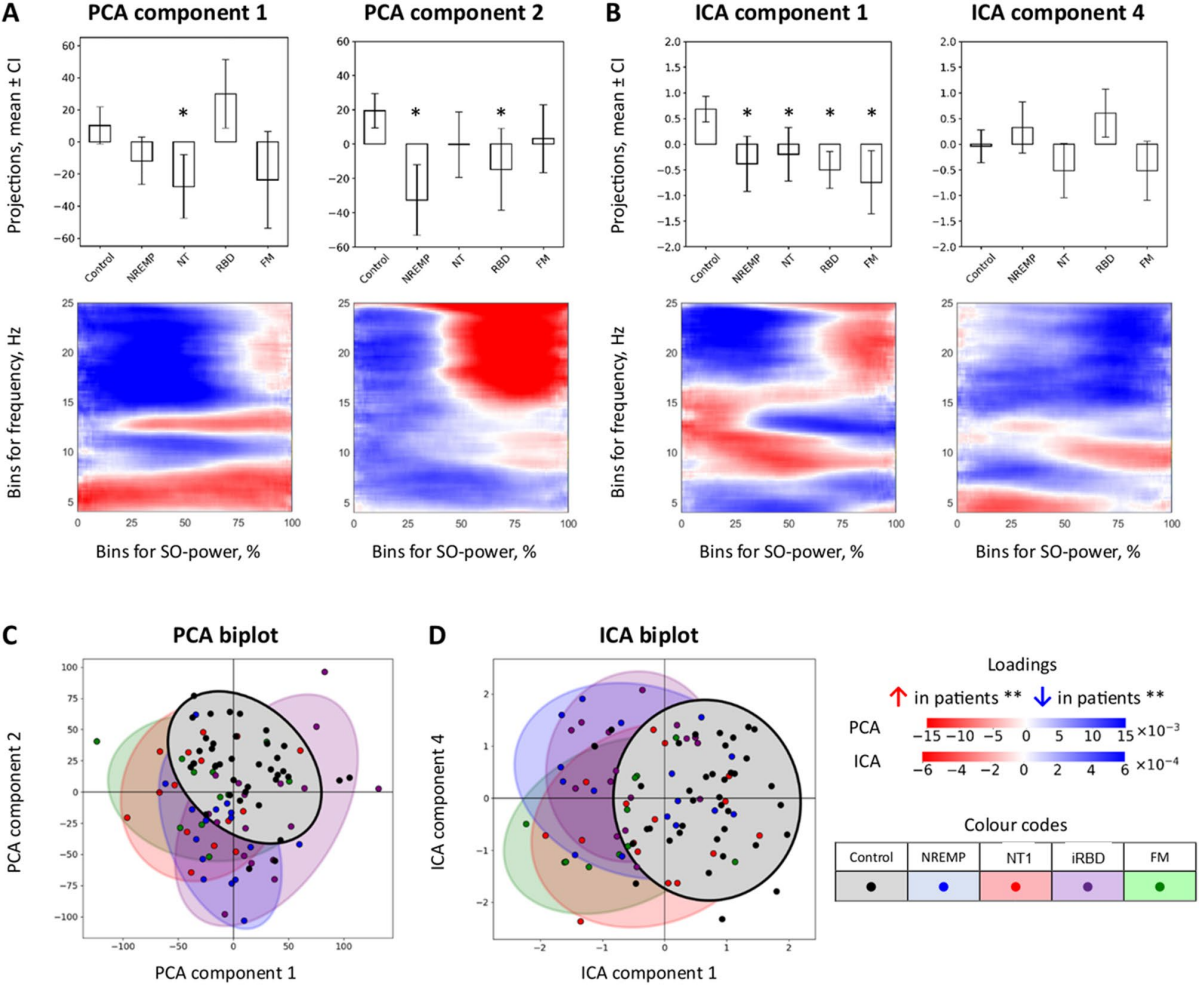
PCA and ICA projections in channel F4 (NREM + REM). (**A**) Boxplots of PCA projection scores for SO-power components in F4 that showed significant between-group differences. PCA Component 1 primarily separates NT1 from controls, whereas Component 2 differentiates NREMP and iRBD. (**B**) Boxplots of ICA projection scores for components capturing shared variance across clinical groups, with ICA Component 1 showing broadly reduced scores in patients relative to controls. Significance is determined by non-parametric Mann–Whitney tests with Bonferroni correction (*p* ≤ 0.05) and is not based on visual overlap of confidence intervals; asterisks (*) denote significant differences versus controls. (**C**) PCA biplot of individual subjects in the space of Components 1 and 2, coloured by group, illustrating partial separation of NT1, NREMP and iRBD from controls. (**D**) ICA biplot of individual subjects in the space of two leading ICA components, with group-wise confidence ellipses. Together, these projections demonstrate that TF-peak–SO-power structure in F4 carries information that discriminates clinical groups from controls. For a comprehensive summary of SO-power components and statistics, see Fig. 3, Supplementary Tables S2–S16, and Supplementary Table S1. *CI* 95% confidence interval, *FM* fibromyalgia, *Hz* hertz, *ICA* independent component analysis, *NREM* non-rapid eye movement sleep,*NREMP* non-REM parasomnia, *NT1* narcolepsy type 1, *PCA* principal component analysis, *iRBD* idiopathic/isolated rapid eye movement sleep behaviour disorder, *REM* rapid eye movement sleep, *SO* slow oscillation, *%* percent

**Fig. 5:**
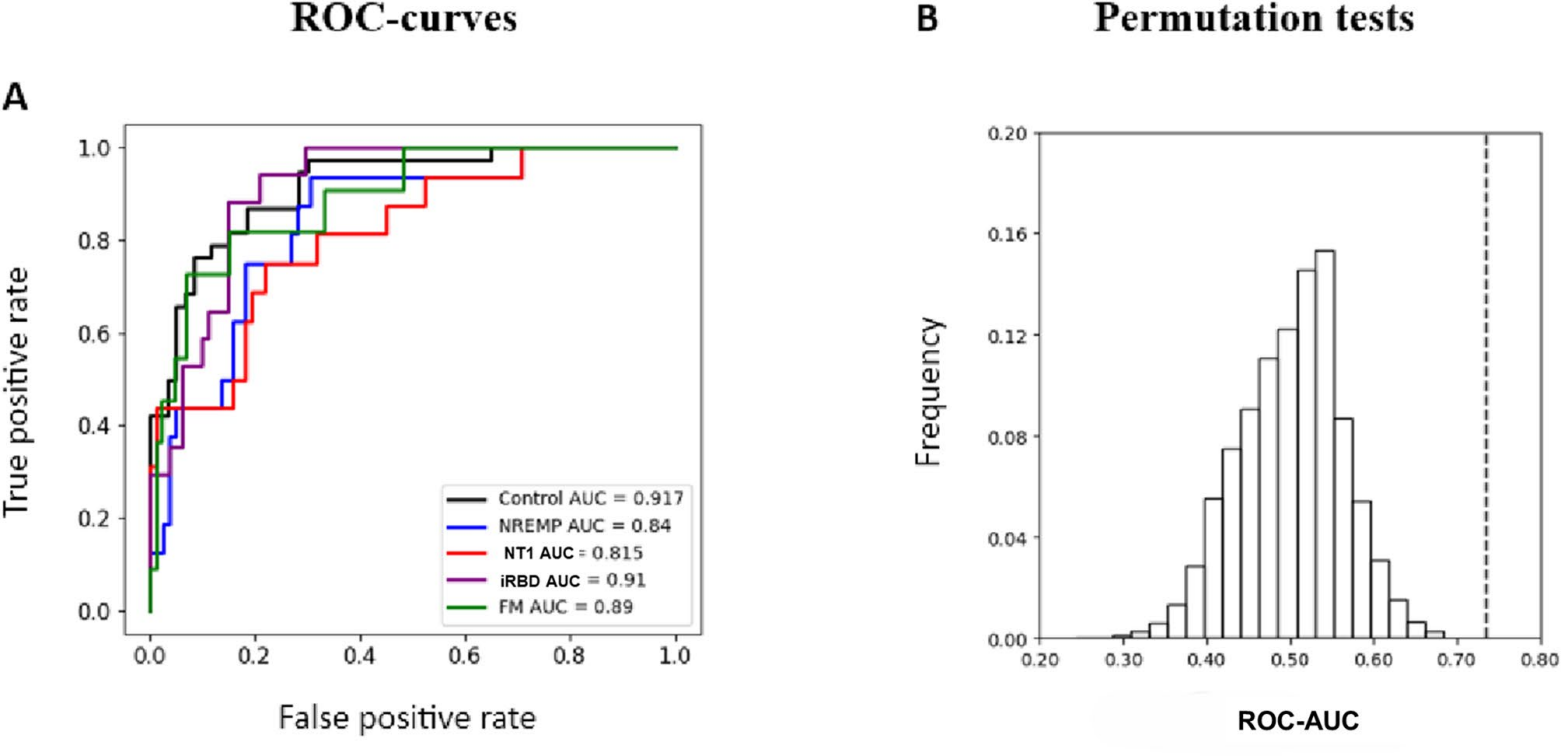
Group-level discrimination based on PCA-derived SO-power features in channel F4.(**A**) Receiver operating characteristic (ROC) curves for one-vs-all logistic regression classifiers trained on PCA projection scores from F4 SO-power histograms (NREM + REM). Each curve illustrates the discriminative performance for one group versus all others under five-fold stratified cross-validation, quantified by the area under the curve (AUC). Apparent discriminability is highest for NT1, NREMP and iRBD (AUC ≳ 0.8). (**B**) Histograms of ROC-AUC values from 1,000 label-shuffle permutation tests (null distributions) and the corresponding true ROC-AUC (dashed line) for each classifier. In all cases, the observed ROC-AUC exceeds the bulk of the null distribution, indicating that the classification performance is unlikely to arise by chance. These analyses are exploratory and illustrate the potential of TF-peak–SO-power features for non-invasive group differentiation. Additional classification and permutation results for other channels and histogram types are provided in Supplementary Figures S14–S17, and group characteristics are shown in Supplementary Table S1. *AUC* area under the ROC curve, *FM* fibromyalgia,*NREM* non-rapid eye movement sleep, *NREMP* non-REM parasomnia, *NT1* narcolepsy type 1, *PCA* principal component analysis; *ROC* receiver operating characteristic,*iRBD* idiopathic/isolated rapid eye movement sleep behaviour disorder, *REM* rapid eye movement sleep, *SO* slow oscillation, *TF-peak*, time–frequency peak.

We next evaluated whether SO-power features could support group-level discrimination using exploratory logistic regression classifiers (SI, Figure S14). Models were trained on principal component projection scores and assessed using internal five-fold cross-validation. Performance, quantified via the area under the receiver operating characteristic curve (AUC), was highest in frontal and central EEG derivations. Classifiers’ ability to distinguish each of the groups from others was measured by the area under the receiver operating characteristic curve metric (ROC-AUC) with the following results in the channel F4: 0.917, 0.840, 0.815, 0.910, and 0.890 for Control, NREMP, NT1, RBD, and FM, respectively. Please refer to Figs. 4 and 5.

To assess robustness, we conducted permutation tests using 1,000 label shuffles (SI, Fig. S16). In all cases, the true model performance exceeded the null distribution, yielding p-values below 0.05.

### SO-phase histograms

#### Group-level distributions

In a complementary analysis, we examined the phase alignment of transient oscillatory events relative to the slow oscillatory cycle. Among healthy controls (please see Fig. 2), fast sigma (12–16 Hz) TF-peaks reliably clustered around the SO trough (0 radians), a pattern consistent with previously reported^14^ SO-spindle coupling. Here, the x-axis again indexes peak frequency, the y-axis bins SO phase from –π to π radians, and colour indicates the proportion of TF-peaks occurring at each frequency–phase combination. As expected, this organization was evident in NREM sleep and combined NREM + REM sleep stages, but absent during REM sleep, where SO amplitude and rhythmicity are diminished.

Patient groups displayed distinctive alterations in this phase-coupling architecture. In NT1 and NREMP, fast sigma (12–16 Hz) TF-peak distributions were broader and showed reduced clustering around the SO trough (0 radians), with relative increases in earlier SO phases. This pattern reflects phase dispersion and altered temporal alignment relative to the slow oscillatory cycle. iRBD participants showed preserved spectral profiles but a marked attenuation in phase-locking, alongside reduced fast sigma density, suggesting disrupted temporal coordination in the context of partially preserved oscillatory structure. FM participants, by contrast, did not exhibit a consistent group-level shift, although their phase histograms were more granular and less structured, pointing to increased variability. Please refer to Fig. 2 and SI, Fig. S6-S9.

#### Component patterns and statistical tests

Dimensionality reduction via PCA and ICA identified seven consistent phase-coupling components, labelled Pha1 through Pha7 (SI, Fig. S10-S11). For instance, the Pha1 component, which characterized tight sigma coupling to the SO trough, was significantly diminished in iRBD, reflecting the loss of coherent phase alignment. The Pha2 component, showing an inverted phase preference, was elevated in NT1 and indicative of early-phase shifts. Pha6 captured phase dispersion across broader frequency ranges (mainly 12–15 Hz) and was specific for NREMP. These deviations were supported by Kruskal–Wallis and Mann–Whitney tests with appropriate correction for multiple comparisons (please see SI, Tables S17-S28).

Effect size analyses reinforced these findings and align with our hypothesis that phase-based coupling metrics are sensitive to circuit-level disruption, even when spectral power remains preserved.

#### Stage-specific analyses

The specificity of phase-coupling alterations to sleep stage was further evaluated (SI, Fig. S10-S11). Across all derivations, significant group differences were confined to NREM sleep and NREM + REM sleep conditions; no components reached statistical significance in REM-only sleep analyses. This asymmetry supports the notion that SO-phase coupling, and its pathological disruption, are most robust during NREM sleep, when cortical synchronization is highest and thalamocortical gating mechanisms are most active. Although REM sleep may still carry relevant oscillatory information, its reduced SO amplitude likely limits the reliability of SO-referenced phase-based metrics during this stage.

#### Reliability and classification

Split-half analysis confirmed the reproducibility of phase-based PCA components, with the first five components again exhibiting correlation coefficients above |0.7| in most of the cases (SI, Fig. S13). However, discriminative modelling using these features yielded more variable performance: AUC values ranged from 0.717 to 0.902 in frontal channels, but permutation tests did not consistently achieve statistical significance (SI, Fig. S17). Compared to SO-power features, SO-phase features appeared to exhibit lower effective signal-to-noise ratios and higher inter-subject variability, particularly in clinical groups, as suggested by the more diffuse component loadings and broader projection distributions (SI Fig. S10–S11, S13). These observations suggest that while SO-phase patterns offer mechanistic insight, their standalone discriminative power in this dataset may be more limited in practice.

## Discussion

This study characterizes the temporal and spectral architecture of transient oscillatory activity in sleep, revealing stage-specific and disorder-specific deviations in TF-peak distributions across four clinical and one control groups. By anchoring TF-peak dynamics to continuous slow oscillatory (SO) power and phase metrics, we move beyond traditional event-based approaches and provide a framework for identifying subtle disruptions in thalamocortical and cortico-subcortical coordination. Our results suggest that transient sleep oscillations may encode robust inter-individual signatures in health, while exhibiting systematic and physiologically interpretable alterations in neurological and sleep disorders. Within the constraints of a six-channel montage, the component topographies we identified map reasonably onto known generators of sleep oscillations: frontal sigma-band components likely index medial prefrontal and anterior thalamic circuits; central components correspond to classical sensorimotor thalamocortical loops; and occipital components capture posterior cortico-thalamic projections^4,13^. The disorder-specific combinations of altered sigma-band SO-power and SO-phase coupling we observe therefore point to selective vulnerabilities within these broader networks, brainstem–cortical pathways in iRBD, orexin-dependent thalamocortical modulation in NT1, and more distributed fronto-parietal disinhibition in NREMP and FM, rather than to a unitary “global” sleep deficit.

**Fig. 6:**
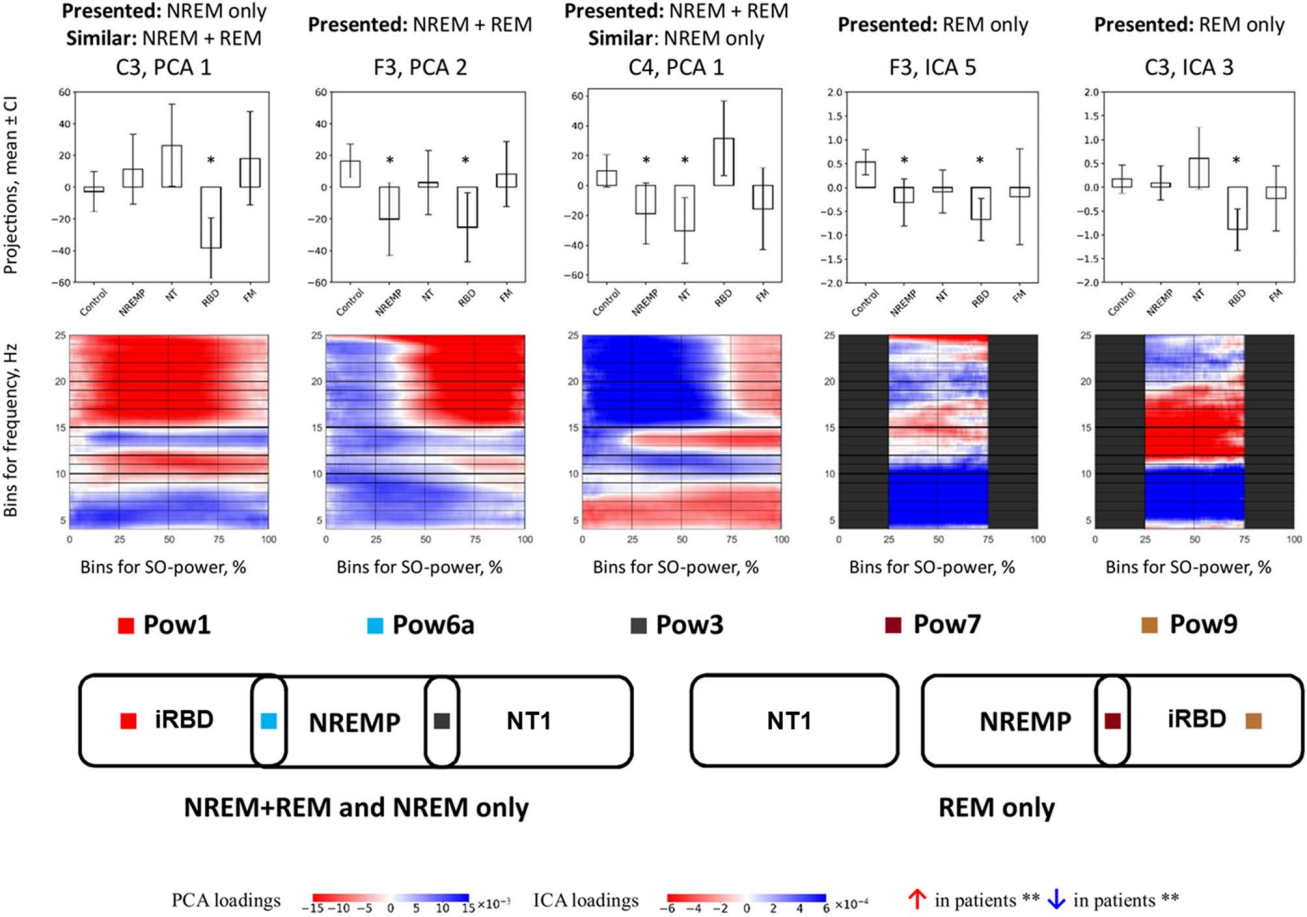
Core SO-power pattern differences across groups. Representative PCA and ICA components corresponding to key SO-power patterns (Pow1, Pow3, Pow6a, Pow7, Pow9) that exhibited significant differences between controls and at least one patient group. Each panel shows (top) boxplots of subject-level projection scores and (bottom) the associated SO-power loading map. Pow1 and Pow9 highlight alterations primarily in iRBD; Pow3 shows changes in NT1 and NREMP; Pow6a and Pow7 capture additional deviations in NREMP and iRBD. Across patterns, the most prominent differences occur in the sigma range (approximately 10–15 Hz) at higher SO-power levels. Significance is determined by non-parametric Mann–Whitney tests with Bonferroni correction (*p* ≤ 0.05) and is not inferred from visual overlap of confidence intervals in the boxplots; asterisks (*) denote significant differences versus controls. Red and blue regions in the loading maps indicate increased and decreased TF-peak density, respectively, associated with higher component scores; patterns are oriented to allow a consistent interpretation across groups. For a full summary of SO-power results and statistical metrics, see Fig. 3, Supplementary Tables S2–S16, and Supplementary Table S1. *CI* confidence interval, *FM* fibromyalgia, *Hz* hertz, *ICA* independent component analysis, *NREM* non-rapid eye movement sleep, *NREMP* non-REM parasomnia, *NT1* narcolepsy type 1, *PCA* principal component analysis, *Pow1–Pow9* SO-power patterns 1–9, *iRBD* idiopathic/isolated rapid eye movement sleep behaviour disorder, *REM* rapid eye movement sleep, *SO* slow oscillation, *%* percent.

In NT1, SO-power histograms (Fig. 2) and component projections (Figs. 3 and 6) revealed significant changes in fast sigma (12–15 Hz) activity at high SO-power levels, accompanied by dispersed SO-phase coupling (Fig. 3 and Supplement, Fig. S10 and S11). These effects suggest impaired recruitment and timing of spindle-like sigma activity during periods of cortical synchrony. Rather than demonstrating changes in classical spindle counts, our TF-peak-based metrics point to altered coupling of sigma-band events to high SO-power states and to shifts in preferred SO-phase angles, consistent with a disruption in the temporal precision of thalamocortical feedback, likely attributable to orexinergic dysfunction^22^. Canonical work has linked slow oscillation–spindle coupling, particularly the alignment of fast spindles to the SO trough, to memory consolidation and emotional regulation^23,24^, and spindles are understood to be the main physiological substrate of sigma-band activity in N2 and N3 sleep. Although our analyses span all NREM sleep stages and do not explicitly isolate N2, the observed temporal desynchrony, if confirmed in larger and longitudinal samples, may help explain cognitive and affective disturbances commonly observed in NT1^25^. These findings highlight the translational potential of TF-peak–based metrics as non-invasive indicators of thalamocortical instability in sleep–wake disorders.

In NREMP, we identified altered TF-peak density within both slow and fast sigma bands during periods of elevated SO power, though the direction and magnitude of these effects varied across cortical derivations and component topographies (Fig. 2a; Supplementary Fig. S3). These disruptions were most evident in components such as Pow2 and Pow6a, which exhibited significant group-level deviations alongside substantial inter-individual variability (Fig. 3; Supplementary Tables S6–S7). Complementing these spectral shifts, NREMP patients also displayed broadened SO-phase distributions in sigma frequencies (Supplement, Figure S10, Pha6), possibly indicative of reduced temporal precision in thalamocortical coordination. Such phase dispersion and density instability are consistent with impaired inhibitory gating and cortical disinhibition, mechanisms thought to underlie the arousal-prone transitions characteristic of parasomnias^26^. The localization of these alterations to NREM sleep, the stage most vulnerable to state dissociation, further argues their pathophysiological relevance^26^. Although behavioral arousals were not quantified here, the observed microstructural instabilities suggest that TF-peak dynamics may encode a latent susceptibility to sleep-wake fragmentation^27,28^. Future studies integrating high-resolution electroclinical data could determine whether these spectral-phase features anticipate abnormal motor behaviors or dissociative transitions, thereby offering a mechanistic bridge between neural dynamics and clinical expression^26,29^. Mixed-frequency components such as Pow6 and Pha6, which combine sigma and neighbouring beta or theta bands, are more difficult to interpret mechanistically. One possibility is that they reflect transient excursions into local arousal or microstate transitions in which sigma activity co-occurs with higher-frequency desynchronisation^27,30^. The prominence of such components in NREMP and NT1 is consistent with models that posit unstable boundaries between sleep and wake states in these disorders^26,29^, but these hypotheses will require direct testing with higher-density and multimodal recordings.

iRBD was distinguished by a specific profile characterised by reduced fast sigma density and markedly diminished phase coupling (Figs. 3 and 6). This decoupling was evident in NREM sleep and in NREM + REM sleep analyses; no SO-phase components reached significance in REM-only sleep analyses, consistent with the lower SO amplitude and less reliable phase estimates in this stage. Importantly, although sigma-band activity remained most prominent over frontal and central derivations, the combination of reduced event density and weakened alignment to the SO trough indicates a disruption in the temporal coordination of thalamocortical circuits, despite the presence of morphologically spindle-like oscillations. This dissociation between preserved structure and impaired timing aligns with evidence for early degeneration of brainstem nuclei and their cortical projections in α-synucleinopathies^31–33^. Conventional spindle metrics, which prioritise amplitude or count, may therefore overlook early dysfunction. The disruption of SO-coupled temporal structure in iRBD may serve as a physiological marker of subcortical-cortical disintegration, potentially refining early detection strategies in at-risk populations^34^.

Of note, FM exhibited the most spatially and spectrally circumscribed alterations among the clinical groups^35^. Within our TF-peak framework, we did not observe robust group-level differences in sigma-range TF-peak density or SO-phase coupling relative to controls, but increased TF-peak activity was evident in the theta (4–6 Hz) and low-alpha (8–10 Hz) bands during NREM + REM combined sleep stages (Figs. 2 and 3), with frontal and occipital derivations most affected^30^.This enhancement of low-frequency activity may reflect increased cortical excitability or sensory gain, possibly consistent with prior reports of alpha intrusions and heightened arousability in FM^30,36^. The absence of fast sigma or phase-coupling disruptions suggests that thalamocortical rhythm generators remain functionally intact, despite localised spectral deviations. These findings align with the interpretation of FM as a condition of altered perceptual filtering rather than global network dysfunction, and they highlight the sensitivity of TF-peak methods to subtle, non-structural alterations in sleep microarchitecture.

Stage-specific analyses confirmed that the most robust deviations occurred during NREM sleep. Across all disorders, NREM sleep histograms and components showed clearer group differences relative to controls than REM-only sleep modes (Figs. 3 and 6, SI Fig. S5, S10–S11). REM sleep-stage data, although more variable, still proved informative in iRBD through SO-power features, but we did not observe statistically robust SO-phase decoupling when REM sleep was analysed in isolation. This asymmetry underscores the differential vulnerability of NREM and REM sleep to circuit-level disruption. While NREM sleep remains the dominant substrate for large-scale oscillatory synchrony, REM sleep may still expose more subtle instabilities, particularly in conditions involving degeneration or dysregulation of subcortical modulatory pathways.

### Clinical implications

These findings indicate that transient oscillatory dynamics, referenced to slow oscillation (SO) power and phase, may offer a principled means of probing thalamocortical and subcortical function. The observed alterations, disorder-specific in topology and stage sensitivity, support a view of sleep microstructure as a physiologically meaningful readout of underlying circuit dynamics^11,14^. Importantly, such features are not only mechanistically interpretable but also clinically accessible, requiring minimal instrumentation and offering millisecond-level temporal precision^14^.

This approach may, thus, carry translational utility across a range of diagnostic contexts. For instance, the attenuation of SO-coupled phase synchrony in iRBD could serve as an early physiological indicator of brainstem-cortical disintegration, potentially anticipating neurodegenerative trajectories^37^. In NT1, reduced spindle-SO alignment may reflect latent thalamocortical dysregulation, with relevance to cognitive and affective symptoms. More broadly, TF-peak–derived signatures may aid in differential classification where behavioural phenotypes are ambiguous, and in refining diagnostic boundaries across syndromic spectra such as parasomnia and hypersomnia.

Beyond diagnosis, the high dimensionality and temporal specificity of this method may support longitudinal tracking in clinical trials, where changes in oscillatory coordination could precede overt symptomatology. Compared to conventional sleep metrics, such as spindle density or stage proportions, TF-peak histograms may arguably provide a richer, state-dependent fingerprint of network behaviour^14^. Future studies incorporating longitudinal designs, cognitive phenotyping, and pharmacological modulation may clarify whether these microstructural features anticipate clinical progression, therapeutic response, or phenotypic conversion.

### Limitations and future directions

This work has several limitations that should temper interpretation. The study was conceived as exploratory and is based on modestly sized clinical samples, particularly in an exploratory fibromyalgia cohort (*n* = 11). Such numbers are common in overnight EEG studies of well-phenotyped sleep pathology, yet they inevitably constrain statistical power, especially for subtle or spatially restricted effects, and limit the generalisability of the findings. Deviations observed in FM and NREMP, particularly those confined to a single derivation or narrow frequency band, should therefore be regarded as provisional and in need of replication in larger, independently recruited cohorts. The marked clinical heterogeneity within both FM and NREMP syndromes further reinforces the need for future work in prospectively assembled samples with more granular symptom profiling^29,38–40^.

The clinical and control groups also differed on several aspects of sleep architecture, including total sleep time, sleep onset latency, N2 and N3 percentages of total sleep, and arousal index (Table S1). Some of these differences were counter to what might be anticipated from classical clinical descriptions; for example, the NT1 group in this sample showed a higher mean N3% and a longer mean REM sleep latency than controls. These patterns almost certainly reflect the modest cohort sizes, retrospective recruitment, and clinical variability, but they nonetheless introduce uncertainty. We did not formally adjust TF-peak, PCA, or ICA features for age or for these architecture variables, and thus cannot determine to what extent group differences in TF-peak structure are mediated by such factors. Although participants were screened for medications known to markedly alter sleep architecture, the use of other centrally acting agents (such as analgesics in FM or residual stimulants in NT1) cannot be fully excluded, and smoking status and milder psychiatric comorbidities were not systematically recorded. Taken together, these considerations impose important constraints on generalisability and underscore the need for larger, prospectively phenotyped cohorts with harmonised clinical, pharmacological, and lifestyle data, in keeping with recent calls for standardised, open sleep datasets^41,42^.

There are also limitations inherent to the recording and signal properties. The use of a standard six-channel EEG montage necessarily restricts spatial resolution and precludes precise source-level inference. With six scalp sites we cannot disentangle thalamocortical from cortico-cortical or brainstem–cortical contributions to the observed patterns; at best, we can attribute components to broad frontal, central, or occipital territories. More anatomically specific insights into the organisation of transient oscillatory dynamics will require high-density EEG, magnetoencephalography, or combined EEG–MEG with explicit source modelling^13,20,43^. In addition, the control and patient datasets were originally acquired with different reference schemes. All data were subsequently re-referenced and standardised offline, and all statistical analyses were channel-specific rather than pooled across sites, yet residual variance attributable to acquisition differences cannot be entirely discounted. The fact that component patterns and group differences were observed across multiple derivations, and were not confined to any single site, is reassuring but does not fully remove this potential confound.

From a methodological standpoint, the decomposition and classification framework also imposes constraints. PCA and ICA provide powerful yet idealised representations of the underlying TF-peak structure, and both are sensitive to preprocessing choices and inter-dataset variability. ICA, in particular, lacks a natural component ordering and can be less stable across subsamples; for this reason, we placed greater emphasis on PCA components, whose eigenvectors showed good split-half reproducibility. Alternative dimensionality-reduction strategies, such as non-linear manifold learning or supervised embeddings, might reveal structure not captured here and could be explored in future work^44,45^. Furthermore, phase metrics in scalp EEG are inherently noisier than power-based measures, especially during REM sleep, in which slow oscillation amplitude is low and phase estimates are less reliable. Observed SO-phase effects, particularly in REM sleep and in smaller clinical groups, should therefore be interpreted with appropriate caution, and we deliberately emphasised the more robust SO-power-coupled findings in our mechanistic discussion.

The classification analyses, while encouraging, must also be viewed in context. Apparent performance was strongest for NT1, iRBD, and NREMP; however, all models were trained and evaluated using cross-validation within the same dataset that was used to construct the PCA feature space. This design raises a non-trivial risk of overfitting, even with regularisation and permutation testing, and precludes any firm diagnostic claims. Moreover, the groups under study were diagnostically well-defined and non-overlapping, conditions that tend to enhance separability relative to the more ambiguous presentations encountered in clinical practice. External validation in independent cohorts, ideally including patients with overlapping symptoms, mixed or uncertain diagnoses, and common comorbidities, will be essential before TF-peak-based classifiers can be considered for translational use. The establishment of shared, publicly accessible benchmarking datasets for TF-peak analyses could further support reproducibility and comparison across laboratories^41,42^.

Finally, this study is cross-sectional and cannot establish causal or longitudinal relationships between transient oscillatory dynamics and clinical outcomes. In idiopathic RBD, for example, it remains unknown whether the observed deviations in SO-coupled sigma activity anticipate subsequent phenoconversion to synucleinopathy or simply index current network dysfunction^46–48^. Similarly, we did not systematically examine correlations between TF-peak metrics and symptom severity, such as cataplexy frequency in NT1, episode burden in NREMP, or pain and fatigue scores in FM, because clinical rating scales were not uniformly available across cohorts and any post-hoc analyses would have been severely underpowered. Addressing these questions will require dedicated, longitudinal studies in at-risk and early-stage populations, with harmonised clinical measures and repeated polysomnography, to determine whether TF-peak-based features track symptom burden, predict progression, or respond to treatment.

## Conclusion

By mapping transient sleep oscillations onto continuous measures of SO power and phase, this study reveals reproducible alterations across several clinical populations. These deviations, whether reductions in synchronized spindling, phase dispersion, or increased sub-sigma activity, reflect distinct pathophysiological signatures of network disruption. Together, our findings argue for a redefinition of sleep microstructure not in terms of discrete events, but as a spectrum of dynamic, state-dependent oscillatory activity. This paradigm may inform the development of electrophysiological phenotypes in sleep medicine, with future applications in diagnosis, monitoring, and mechanistic understanding.

## Materials and methods

A retrospective exploratory cross-sectional study was conducted on 99 adults (≥ 18 years) across five diagnostic categories: idiopathic REM sleep behavior disorder (iRBD; *n* = 17), narcolepsy type 1 (NT1; *n* = 16), non-REM parasomnia (NREMP; *n* = 16), fibromyalgia syndrome (FM; *n* = 11), and healthy controls (*n* = 39; from The Montreal Archive of Sleep Studies)^41^. All clinical diagnoses were made in accordance with the International Classification of Sleep Disorders – Third Edition (ICSD-3) and confirmed by board-certified sleep specialists^16,17^. Participants included were ≥ 18 years of age and had no major psychiatric or neurological comorbidities, substance dependence, or use of medications known to alter sleep architecture. The clinical cohorts were drawn from consecutive patients referred to the Sleep Disorders Centre at Guy’s and St Thomas’ NHS Foundation Trust, and the healthy controls from the Montreal Archive of Sleep Studies^41^. Inclusion criteria for all participants were: age ≥ 18 years; availability of at least one full-night polysomnogram with standard six-channel EEG; and, for clinical groups, a stable diagnosis according to ICSD-3 criteria^16,17^ confirmed by a board-certified sleep specialist. Exclusion criteria included: history of epilepsy or other major neurological disease (e.g., stroke, traumatic brain injury), moderate to severe obstructive sleep apnoea (apnoea–hypopnoea index > 15 events/hour), severe psychiatric disorder (e.g., psychosis, bipolar disorder), current substance dependence, and use of medications known to markedly alter sleep architecture (e.g. high-dose benzodiazepines, antipsychotics, certain antidepressants). Smoking status and milder psychiatric comorbidities (e.g. past depressive episodes) were not systematically recorded and may therefore introduce residual heterogeneity.

Ethical approval for the study was granted by the institutional Research Ethics Committee (Project No. 12436)^49,50^. The analysis was conducted on fully anonymized retrospective data, in compliance with the UK Data Protection Act and the General Data Protection Regulation (GDPR) (Regulation (EU) 2016/679). Informed consent was not required due to the retrospective design and the use of non-identifiable data^49,50^. The study was carried out in accordance with the Declaration of Helsinki (WMA, 2013).

All overnight polysomnographic (PSG) recordings included standard six-channel EEG using a 10–20 montage (F3, F4, C3, C4, O1, O2). Referencing schemes were harmonized during preprocessing to ensure consistency across datasets. Sleep staging was performed manually in accordance with American Academy of Sleep Medicine (AASM) criteria^51^. Summary socio-demographic and sleep architecture metrics are provided in SI, Table S1.

### EEG preprocessing and TF-peak extraction

EEG preprocessing was conducted using MNE-Python (v1.5.0)^52^ and the DYNAM-O toolbox (version 1.0)^14^. Signals were down-sampled to 100 Hz, bandpass filtered (0.1–40 Hz), and re-referenced. Channels with persistent artefacts were excluded per DYNAM-O’s artefact rejection pipeline. Across all subjects, this procedure resulted in the exclusion of at most one EEG channel per recording; the majority of datasets retained the full six-channel montage. Because the clinical cohorts were relatively small, we did not discard subjects based on channel dropout, but instead analysed each available channel independently while using channel-specific statistical tests to avoid bias.

TF-peaks were extracted using a watershed-based algorithm that identifies local maxima across the 4–25 Hz frequency range^14^. Each TF-peak was annotated by the concurrent SO power (0.3–1.5 Hz) and SO phase at its time of occurrence, yielding two-dimensional histograms per channel for both power- and phase-anchored events.

Histograms were generated for three conditions: NREM + REM sleep combined, NREM-only sleep, and REM-only sleep. For REM sleep, only the interquartile range (25–75%) of SO power was included to reduce noise, reflecting the lower SO amplitude in this stage.

### Statistical decomposition and group comparisons

Each histogram (per stage and channel) was standardized^53^ and submitted to PCA^54^ and ICA^55^ using Scikit-learn (v1.3.2)^56^. For each subject, the resulting PCA and ICA models yielded a set of projection scores, corresponding to that subject’s position along each component. These subject-level projection scores (one value per subject per component per channel and stage) served as the input to all subsequent statistical tests (Kruskal–Wallis across groups and Mann–Whitney pairwise comparisons) and to the logistic regression models used for classification. The first 10 PCA components (capturing ~ 70% of total variance) were retained. ICA was performed with 5 components for SO-power and 3–4 for SO-phase histograms, based on convergence criteria (yielding up to five labelled ICA components per histogram set, as illustrated in SI Fig. S5).

Group comparisons were conducted using Kruskal–Wallis tests^57^ across all five groups, with false discovery rate (FDR) control via the Benjamini–Yekutieli method^58^ (α = 0.1). When significant, post hoc Mann–Whitney U tests^59^ were performed between controls and each patient group, with Bonferroni-adjusted^60^ p-values (*p* ≤ 0.05) reported in SI, Tables S2-S28. η2 effect size^61^ was calculated for all the cases (Tables S2-S28).

### Reliability and classification analyses

Split-half reliability was assessed by randomly dividing subjects into balanced subsets, performing PCA separately, and correlating eigenvectors between subsets and the full sample. Components with Spearman^62^ r > |0.75| (first five PCs) were deemed highly reliable; those with r > |0.5| moderately reliable.

To evaluate the discriminative potential of histogram-derived PCA features, we trained logistic regression models^53,63^ using five-fold cross-validation^64,65^. Each subject served once in the validation fold. Performance in each fold was quantified by the area under the receiver operating characteristic curve (ROC-AUC)^66^. Fold-wise ROC curves and AUC values were then averaged to obtain the summary performance estimates shown in Fig. 5 and SI Figures S14–S15. Statistical significance of classification was tested via 1,000 permutation tests^67^, using ROC-AUC as the test statistic. In each permutation, group labels were shuffled across subjects, the logistic regression model was refit, and ROC-AUC recomputed. The p-value was defined as the proportion of shuffled AUCs greater than or equal to the true (unshuffled) AUC. ROC curves and AUC values were computed from the predicted class probabilities output by the logistic regression model and the true binary group labels (target group vs. all other groups).

### Visualization and software

Data visualizations were generated using DYNAM-O (v1.0)^14^, Matplotlib (v3.8.0)^68^, and Seaborn (v0.13.0)^69^. Statistical calculations were performed by SciPy (v1.11.4)^70^ and Statsmodels (v0.14.1)^58^.

## Supplementary Information

Below is the link to the electronic supplementary material.

Supplementary Material 1

## Data Availability

The clinical polysomnography and EEG recordings analysed in this study contain sensitive health information and are subject to the data-protection policies of Guy’s and St Thomas’ NHS Foundation Trust and King’s College London. In accordance with the conditions of ethics approval (project number 12436) and UK data-protection legislation, raw recordings cannot be shared publicly. Control recordings were drawn from the Montreal Archive of Sleep Studies (MASS), which is publicly accessible as described in reference 41.
