# Supplementary material for: Disorder-specific alterations of transient oscillatory dynamics during sleep across cortical and subcortical networks
Source: Sci Rep. 2026 Jan 20;16:3630. doi: 10.1038/s41598-025-33669-1 (PMC12847891; doi:10.1038/s41598-025-33669-1)
Supplement: Supplementary file 1 — Supplementary Material 1 [file 41598_2025_33669_MOESM1_ESM.pdf]

Supplementary Information (SI)

Table of Contents

1. Sociodemographic and polysomnographic characteristics
1.1 Sociodemographic and sleep architecture measures – Table S1

2. **General pipeline and SO-power histograms**
2.1 Group-averaged SO-power histograms and arithmetic differences
• NREM+REM sleep stages together – Figures S1
• NREM-only and REM-only sleep stages – Figures S2–S4
2.2 Statistically significant SO-power patterns
• PCA/ICA components and pattern maps (Pow1–Pow9) – Figures S5-1–S5-4 (collectively Figure S5)
2.3 Summary of SO-power statistical tests
• NREM+REM sleep stages together – Tables S2–S8
• NREM-only sleep stages – Tables S9–S13
• REM-only sleep stage – Tables S14–S16

3. **SO-phase histograms**
3.1 Group-averaged SO-phase histograms and arithmetic differences
• NREM+REM sleep stages together, NREM-only sleep, and REM-onlysleep – Figures S6–S9
3.2 Statistically significant SO-phase patterns
• PCA/ICA components and pattern maps (Pha1–Pha7) – Figure S10
3.3 Pattern-level summary of SO-phase differences
• Overview of significant patterns across channels and groups – Figure S11
3.4 Summary of SO-phase statistical tests
• NREM+REM sleep stages together – Tables S17–S23
• NREM-only sleep stages – Tables S24–S28

4. **Split-half reliability**
• Split-half correlation matrices for SO-power and SO-phase PCA eigenvectors – Figures S12–S13

5. **Predictive modelling**
• ROC curves illustrating group-level discrimination – Figures S14–S15
• Permutation tests for classifier performance – Figures S16–S17

6. **Global summaries of significant components**
• SO-power components: counts per group and stage – Figure S18
• SO-phase components: counts per group and stage – Figure S19

Table S1. Sociodemographic and polysomnographic characteristics

Variable	Control		NREMP		NT1		iRBD		FM		χ2	p
Gender, % females	48.72		50.00		50.00		11.76		90.91		0.040	.842
**Variable**	**Control**		**NREMP**		**NT**		**iRBD**		**FM**		**U**	**p**
	**Mean**	**SD**	**Mean**	**SD**	**Mean**	**SD**	**Mean**	**SD**	**Mean**	**SD**		
Age (years)	45.949	16.540	39.375	6.869	30.875	11.506	60.176	9.639	45.091	6.534	1288.500	.398
TST (min)	420.969	36.058	382.863	39.840	412.525	49.319	361.176	58.091	339.382	89.307	1697.500	1.6E-04*
WASO (min)	60.313	39.087	52.063	42.734	60.675	27.395	90.929	46.740	106.400	67.992	999.000	.222
SOT (min)	21.621	13.170	10.069	7.667	6.275	4.690	21.159	26.371	30.855	38.433	1686.500	2.2E-04*
SE (%)	84.185	8.262	86.494	8.006	85.956	5.877	76.441	12.031	71.227	16.433	1296.500	.367
SL to N1 (min)	21.621	13.170	10.069	7.667	6.275	4.690	21.159	26.371	30.855	38.433	1686.500	2.2E-04*
SL to N2 (min)	26.236	13.689	14.663	8.196	10.869	7.486	26.718	27.673	45.764	49.438	1614.500	.001*
SL to N3 (min)	43.185	18.689	28.944	13.157	20.244	10.547	36.394	28.433	65.627	55.479	1638.000	.001*
SL to REM (min)	81.910	30.172	87.375	23.370	88.406	27.710	109.618	77.669	89.600	43.982	1020.500	.347
AHI	1.846	0.709	1.281	1.928	1.463	1.377	3.571	3.135	1.282	1.550	1411.500	.083
AI	11.685	3.938	15.669	6.699	17.244	5.036	21.106	8.132	20.355	9.002	477.000	7.1E-07*
N1 % of TST	8.972	4.076	8.425	2.841	10.675	5.693	12.265	5.439	13.836	13.664	939.000	.099
N2 % of TST	47.279	7.086	47.863	8.872	42.775	6.552	42.988	8.980	40.736	7.913	1445.000	.049*
N3 % of TST	21.100	6.016	21.469	8.637	26.119	7.154	24.588	6.948	24.327	7.771	872.500	.033*
REM % of TST	22.636	5.066	22.250	3.778	20.438	5.481	20.176	6.321	21.100	7.446	1321.500	.280

Notes. The table depicts results of statistical tests for Control (n=39) versus the combined patient group (NREMP n=16, NT n=16, iRBD n=17, FM n=11). The Pearson χ2 test was utilized for categorical variables, and the Mann-Whitney test was employed for ordinary variables.

Abbreviations: **AHI,** apnea-hypopnea index; **AI,** arousal index; **DF,** degrees of freedom; **FM,** fibromyalgia; **IQR**, interquartile range; **min,** minutes; **n**, number; **N1, N2, N3,** non-rapid eye movement sleep stages 1,2,3; **NREMP,** non-REM parasomnia; **NT1,** narcolepsy type 1; **p,** p-value; **iRBD,** idiopathic REM behavior disorder; **REM,** rapid eye movement sleep; **SD,** standard deviation; **SE,** sleep efficiency; **SL,** sleep latency; **SOT**, sleep onset time; **Total N,** total number of subjects in 5 groups; **TST,** total sleep time; **U**, Mann-Whitney test statistics; **WASO,** wake after sleep onset; **χ2,** chi squared statistics; **%,** percent.

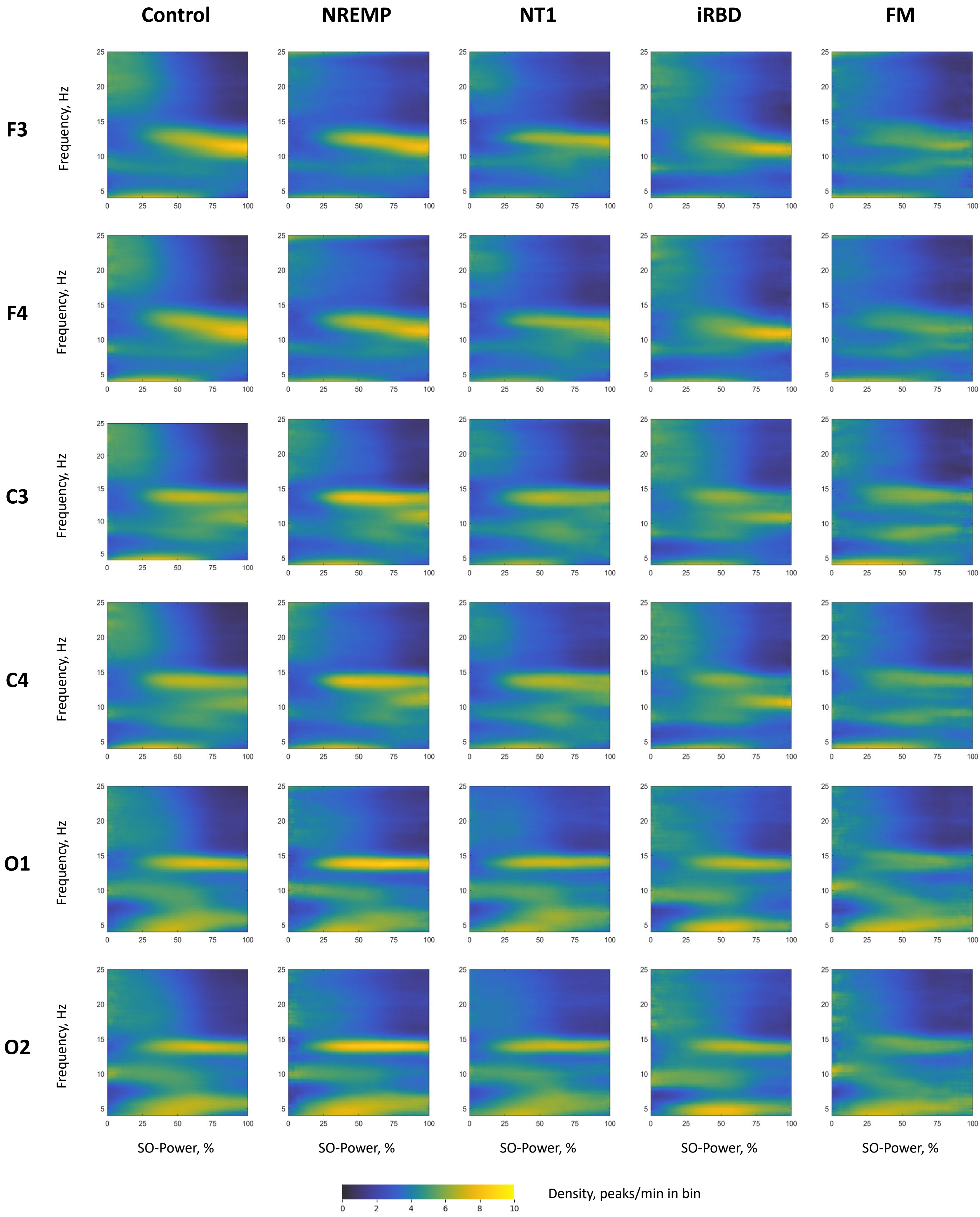

Fig S1. Group average SO-power histograms for NREM + REM sleep stages together. Please refer to **Table S1** for detailed information about the groups.

Abbreviations: **[C3, C4, F3, F4, O1, O2],** channels' codes in the standard 10-20% electroencephalography montage; **FM,** fibromyalgia; **NREM,** non-rapid eye movement stages; **NREMP**, non-REM parasomnia**; NT1**, narcolepsy type 1; **iRBD,** rapid eye movement sleep behavior disorder; **REM,** rapid eye movement stage, **SO,** slow oscillations; **%,** percent.

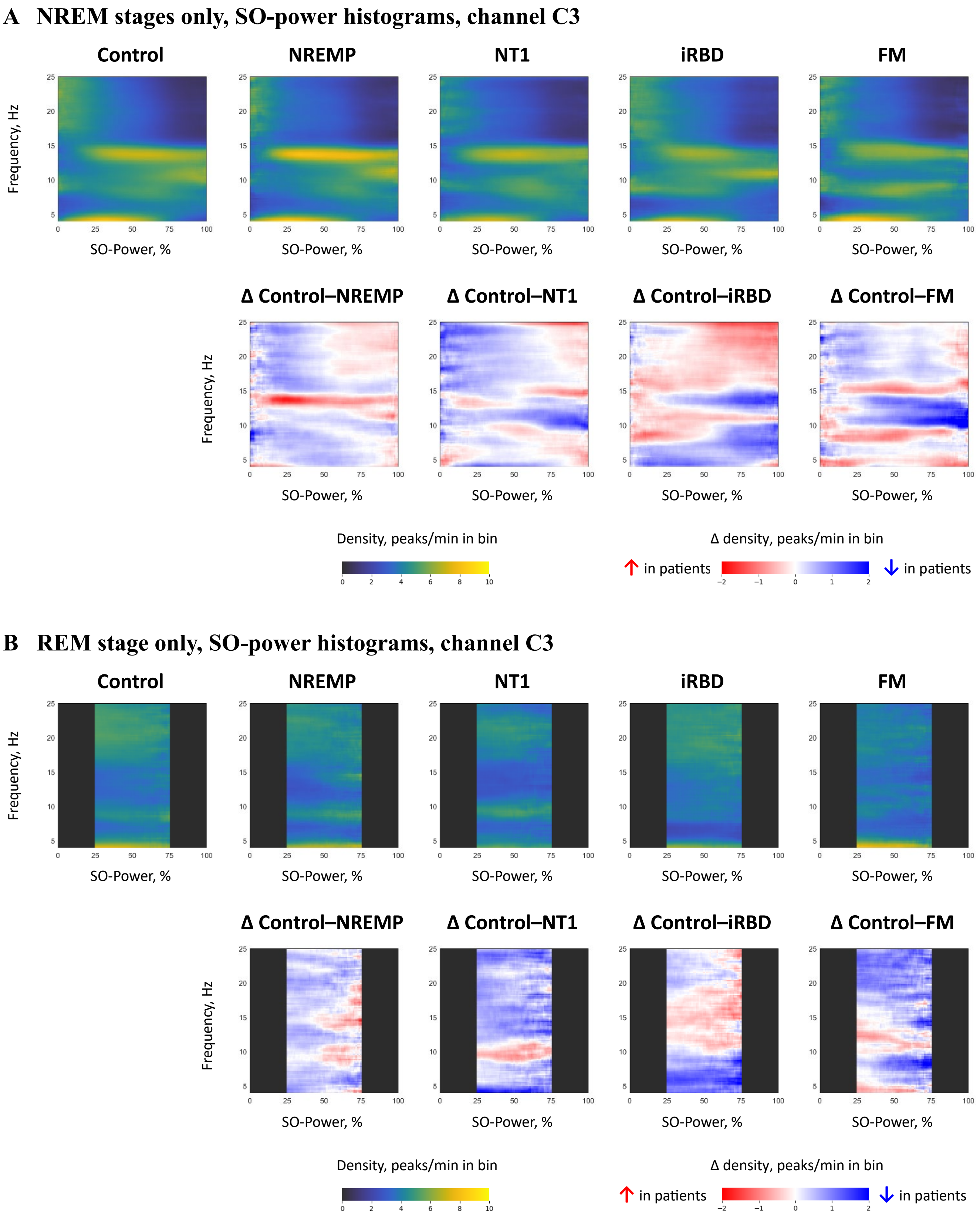

Fig. S2. SO-power histograms for NREM only sleep and REM only sleep stages (A and B, respectively) at channel C3. In each panel, the upper row of histograms illustrate group average, while the lower row shows arithmetical differences between histogram matrices. For REM histograms, only the diapason of 25-75% SO power was considered due to inconsistency in other diapasons (please see **Materials and Methods** section). Please see **Figure 2** for SO-power histograms at NREM+REM stages together and **Table S1** for detailed information about the groups.

Abbreviations: **C3,** channels' codes in the standard 10-20% electroencephalography montage; **FM,** fibromyalgia; **Hz,** hertz; **min**, minute; **NREM,** non-rapid eye movement stages; **NREMP**, non-REM parasomnia**; NT1**, narcolepsy type 1; **iRBD, idiopathic/isolated** rapid eye movement sleep behavior disorder; **REM,** rapid eye movement sleep stage; **SO,** slow oscillations; **%,** percent.

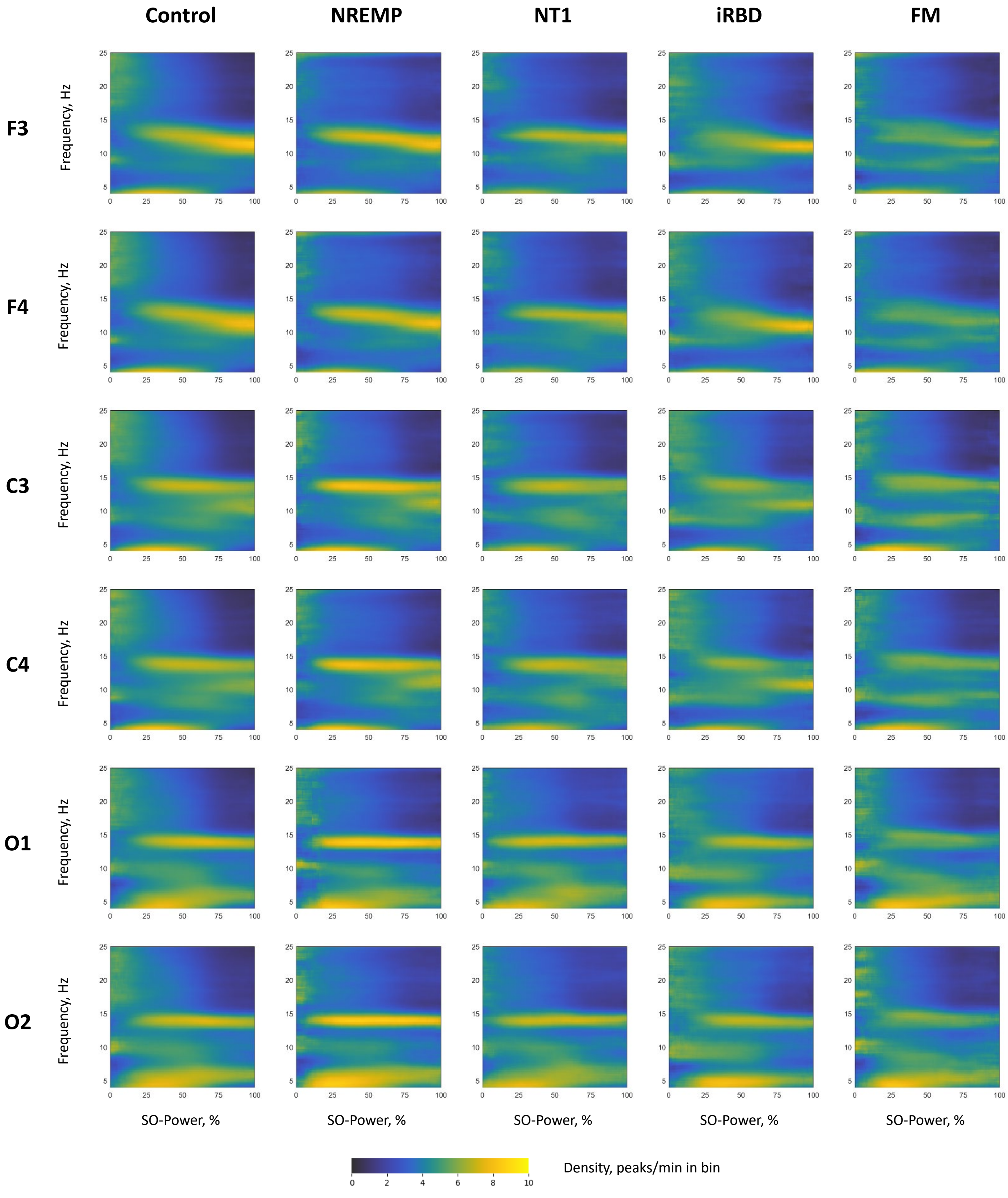

Fig. S3. Group average SO-power histograms for NREM sleep stages only. Please see **Table S1** for detailed information about the groups.

Abbreviations: **[C3, C4, F3, F4, O1, O2],** channels' codes in the standard 10-20% electroencephalography montage; **FM,** fibromyalgia; **NREM,** non-rapid eye movement stages; **NREMP**, non-REM parasomnia; **NT1**, narcolepsy type 1; **iRBD,** idiopathic/isolated rapid eye movement sleep behavior disorder; **SO,** slow oscillations; **%,** percent.

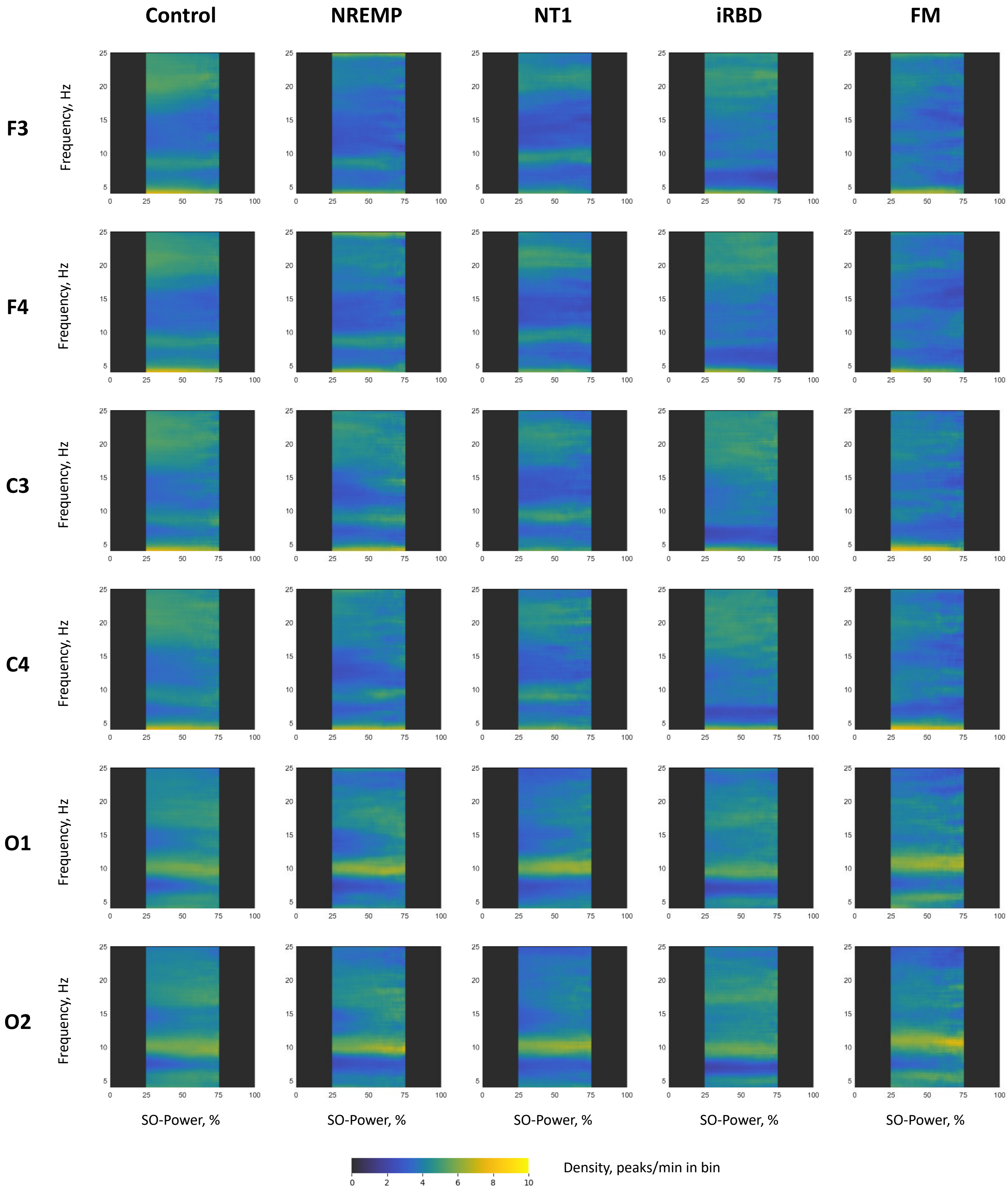

Fig. S4. Group average SO-power histograms for REM sleep stages only. Please see **Table S1** for detailed information about the groups.

Abbreviations: **[C3, C4, F3, F4, O1, O2],** channels' codes in the standard 10-20% electroencephalography montage; **FM,** fibromyalgia; **NREMP**, non-REM parasomnia**; NT1**, narcolepsy type 1; **iRBD,** idiopathic/isolated rapid eye movement sleep behavior disorder; **REM,** rapid eye movement stage; **SO,** slow oscillations; **%,** percent.

Patterns of PCA/ICA components with significant differences between Control and patients, part 1 of 4

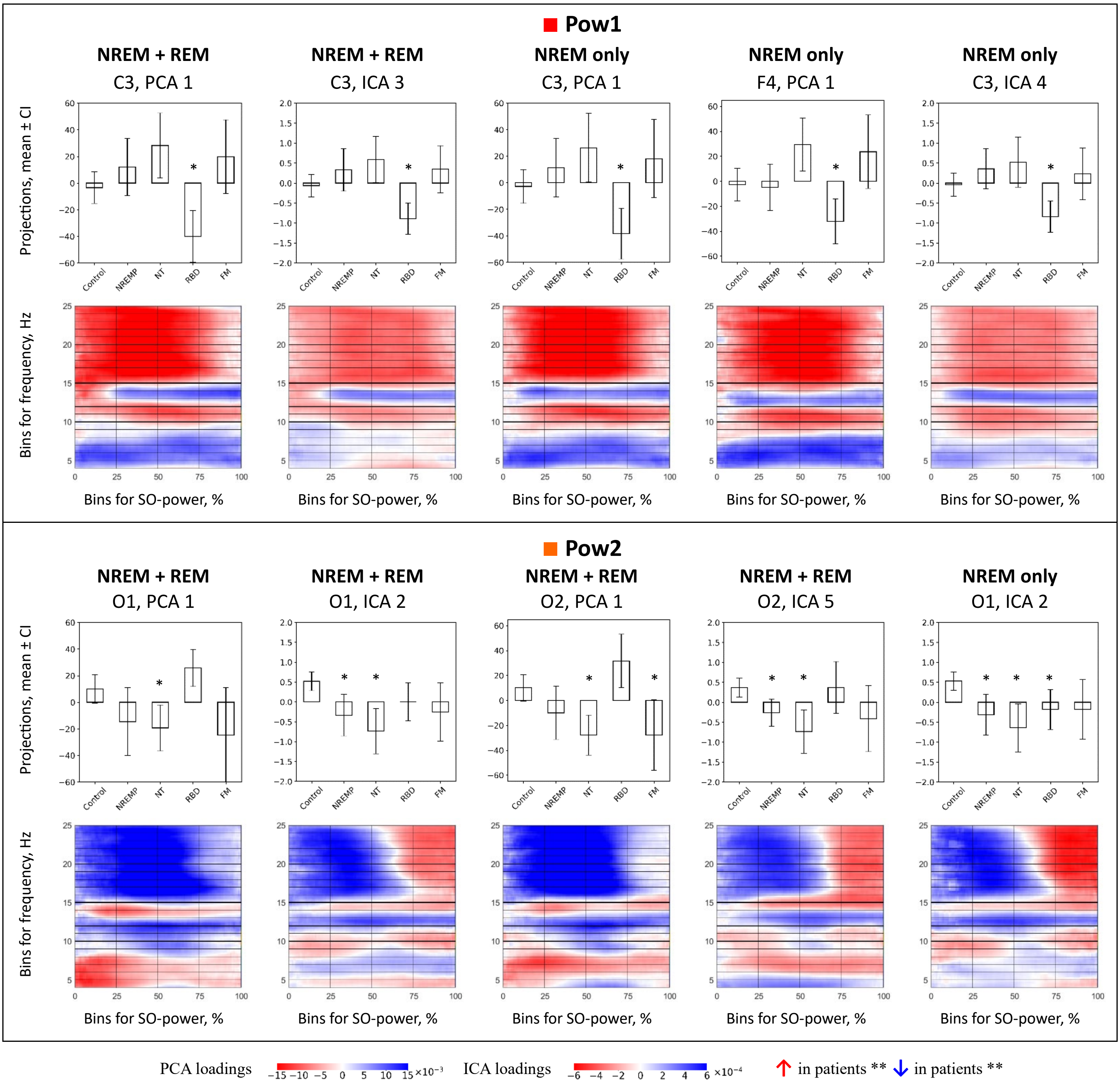

Fig. S5-1. SO-power histograms: PCA and ICA components that were associated with significant differences between groups (pairwise comparisons of Control versus each of the patient groups, p≤.05 Bonferroni adjusted). Grid lines indicate frequency bands (1 Hz resolution) and SO-power quartiles. Thick lines mark bands of slow 10-12 Hz and fast 12-15 Hz sigma. Component histograms were classified into categories Pow1-Pow9 based on the visual similarity of the depicted patterns. Please refer to **Figure 3**, **Tables S2-S16,** and **Table S1** for summary of between-group differences, detailed statistics, and information about the groups, respectively.

* denotes statistically significant Bonferroni-corrected p-values, p ≤ .05.
** red (increase) and blue (decrease) colors indicate these changes in patient groups with significant differences versus Control; all the patterns are oriented in such a way as to unify the interpretation approaches.

Abbreviations: [C3, C4, F3, F4, O1, O2], channel's code in the standard 10-20% electroencephalography montage; **CI,** confidence intervals; **Hz**, hertz; **FM,** fibromyalgia; **ICA,** independent component analysis; **ICA x**, ICA component x; **NREM**, non-rapid eye movement stages; **NREMP**, non-REM parasomnia**; NT1**, narcolepsy; **p,** p-value; **PCA**, principal component analysis; **PCA x,** PCA component x; **Pow1-Pow9**, SO-power patterns 1-9; **iRBD,** idiopathic/isolated rapid eye movement sleep behavior disorder; **REM,** rapid eye movement sleep stage **SO,** slow oscillations; **%,** percent.

Patterns of PCA/ICA components with significant differences between Control and patients, part 2 of 4

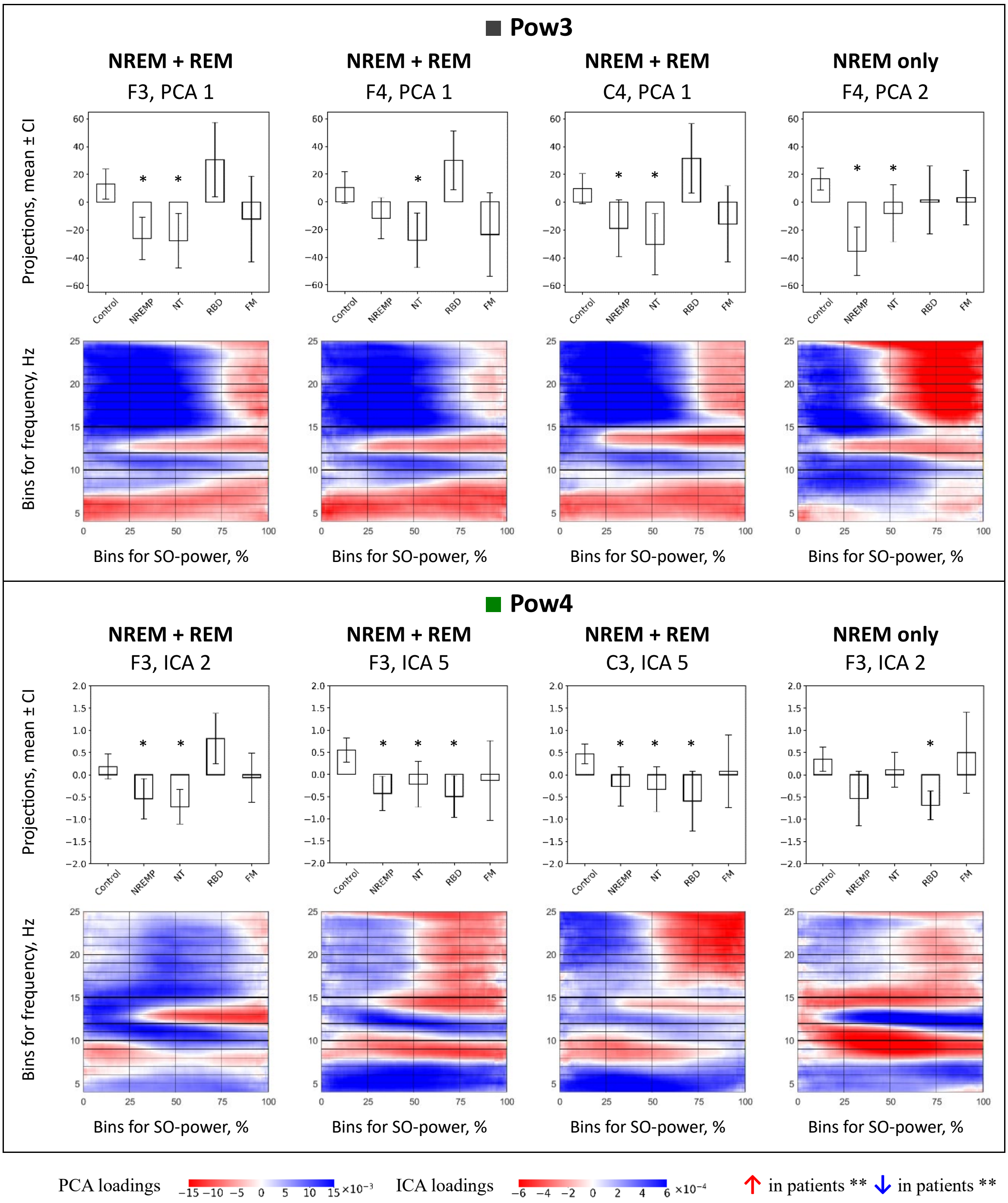

Fig. S5-2. SO-power histograms: PCA and ICA components that were associated with significant differences between groups (pairwise comparisons of Control versus each of the patient groups, p≤.05 Bonferroni adjusted). Grid lines indicate frequency bands (1 Hz resolution) and SO-power quartiles. Thick lines mark bands of slow 10-12 Hz and fast 12-15 Hz sigma.
Component histograms were classified into categories Pow1-Pow9 based on the visual similarity of the depicted patterns. Please refer **Figure 3**, **Tables S2-S16,** and **Table S1** for summary of between-group differences, detailed statistics, and information about the groups, respectively.

* denotes statistically significant Bonferroni-corrected p-values, p ≤ .05.
** red (increase) and blue (decrease) colors indicate these changes in patient groups with significant differences versus Control; all the patterns are oriented in such a way as to unify the interpretation approaches.

Abbreviations: [C3, C4, F3, F4, O1, O2], channel's code in the standard 10-20% electroencephalography montage; **CI,** confidence intervals; **Hz**, hertz; **FM,** fibromyalgia; **ICA,** independent component analysis; **ICA x**, ICA component x; **NREM**, non-rapid eye movement stages; **NREMP**, non-REM parasomnia**; NT1**, narcolepsy type 1; **p,** p-value; **PCA**, principal component analysis; **PCA x,** PCA component x; **Pow1-Pow9**, SO-power patterns 1-9; **iRBD,** idiopathic/isolated rapid eye movement sleep behavior disorder; **REM,** rapid eye movement sleep stage **SO,** slow oscillations; **%,** percent.

Patterns of PCA/ICA components with significant differences between Control and patients, part 3 of 4

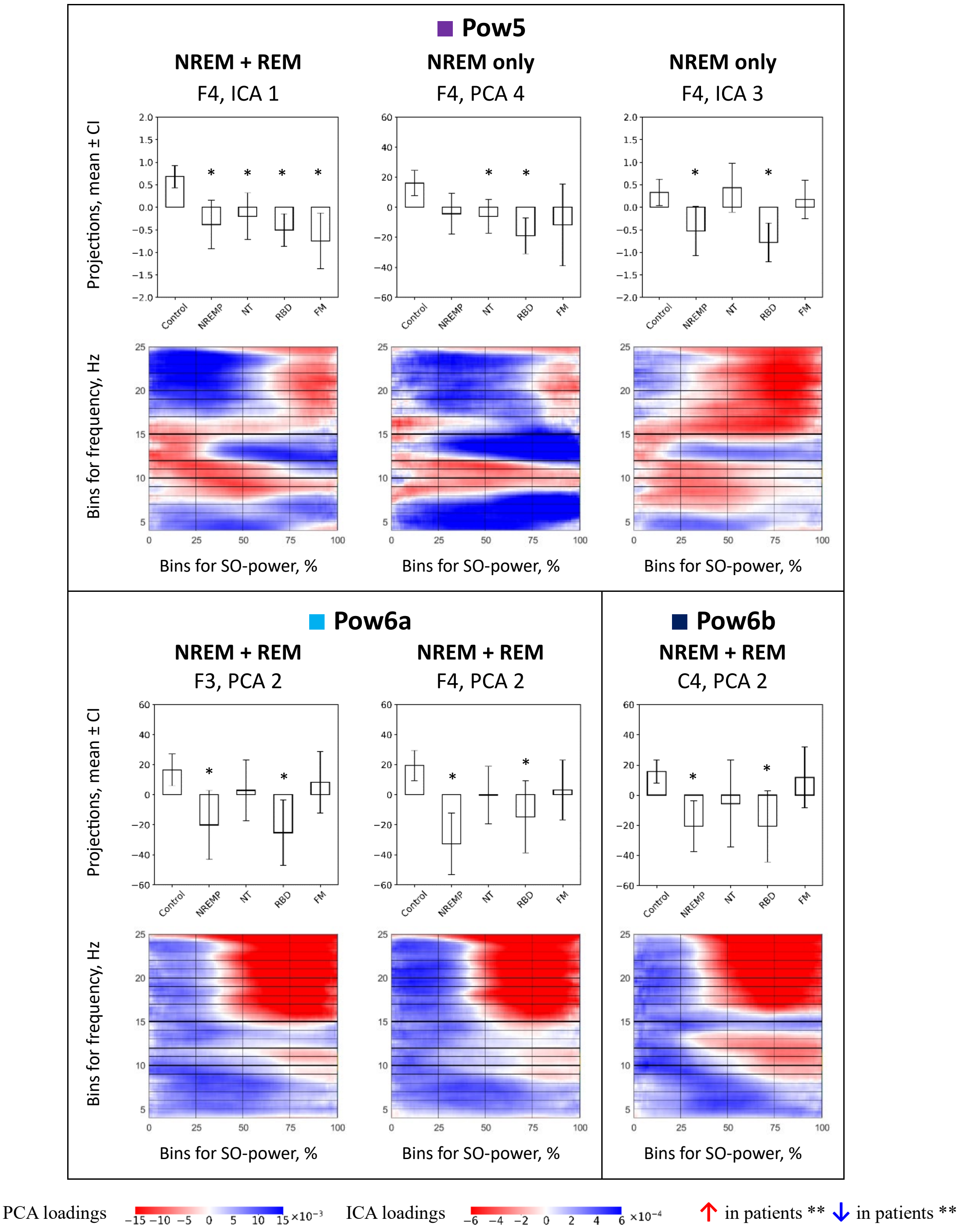

Fig. S5-3. SO-power histograms: PCA and ICA components that were associated with significant differences between groups (pairwise comparisons of Control versus each of the patient groups, p≤.05 Bonferroni adjusted). Grid lines indicate frequency bands (1 Hz resolution) and SO-power quartiles. Thick lines mark bands of slow 10-12 Hz and fast 12-15 Hz sigma. Component histograms were classified into categories Pow1-Pow9 based on the visual similarity of the depicted patterns. Please refer to **Figure 3**, **Tables S2-S16,** and **Table S1** for summary of between-group differences, detailed statistics, and information about the groups, respectively.

* denotes statistically significant Bonferroni-corrected p-values, p ≤ .05.
** red (increase) and blue (decrease) colors indicate these changes in patient groups with significant differences versus Control; all the patterns are oriented in such a way as to unify the interpretation approaches.

Abbreviations: [**C3, C4, F3, F4, O1, O2]**, channel's code in the standard 10-20% electroencephalography montage; **CI,** confidence intervals; **Hz**, hertz; **FM,** fibromyalgia; **ICA,** independent component analysis; **ICA x**, ICA component x; **NREM**, non-rapid eye movement stages; **NREMP**, non-REM parasomnia**; NT1**, narcolepsy type 1; **p,** p-value; **PCA**, principal component analysis; **PCA x,** PCA component x; **Pow1-Pow9**, SO-power patterns 1-9; **iRBD,** idiopathic/isolated rapid eye movement sleep behavior disorder; **REM,** rapid eye movement sleep stage **SO,** slow oscillations; **%,** percent.

Patterns of PCA/ICA components with significant differences between Control and patients, part 4 of 4

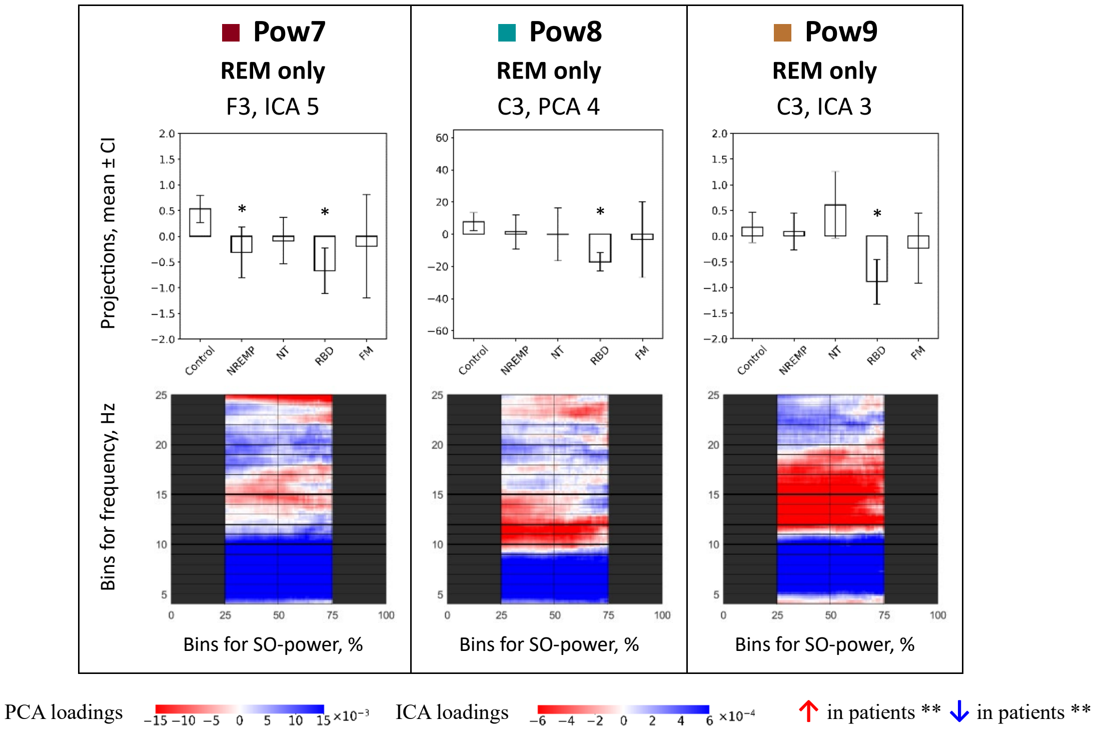

Figure S5-4. SO-power histograms: PCA and ICA components that were associated with significant differences between groups (pairwise comparisons of Control versus each of the patient groups, p≤.05 Bonferroni adjusted). Grid lines indicate frequency bands (1 Hz resolution) and SO-power quartiles. Thick lines mark bands of slow 10-12 Hz and fast 12-15 Hz sigma. Component histograms were classified into categories Pow1-Pow9 based on the visual similarity of the depicted patterns. Please refer to **Figure 3**, **Tables S2-S16,** and **Table S1** for summary of between-group differences, detailed statistics, and information about the groups, respectively.

* denotes statistically significant Bonferroni-corrected p-values, p ≤ .05.
** red (increase) and blue (decrease) colors indicate these changes in patient groups with significant differences versus Control; all the patterns are oriented in such a way as to unify the interpretation approaches.

Abbreviations: [**C3, C4, F3, F4, O1, O2],** channel's code in the standard 10-20% electroencephalography montage; **CI,** confidence intervals; **Hz**, hertz; **FM,** fibromyalgia; **ICA,** independent component analysis; **ICA x**, ICA component x; **NREM**, non-rapid eye movement stages; **NREMP**, non-REM parasomnia**; NT1**, narcolepsy type 1; **p,** p-value; **PCA**, principal component analysis; **PCA x,** PCA component x; **Pow1-Pow9**, SO-power patterns 1-9; **iRBD,** idiopathic/isolated rapid eye movement sleep behavior disorder; **REM,** rapid eye movement sleep stage **SO,** slow oscillations; **%,** percent.

Table S2. Summary of Kruskal–Wallis test between five groups (PCA and ICA components of SO-power histogram datasets; NREM + REM sleep stages together; components with significant differences only)

Channel	Component	Total N	H	DF	η2	p	α
F3	PCA 1	97	26.054	4	0.240	3.086E-05	**.005 ***
F3	PCA 2	97	19.273	4	0.166	6.947E-04	**.027 ***
F3	ICA 2	97	24.083	4	0.218	7.688E-05	**.003 ***
F3	ICA 5	97	24.407	4	0.222	6.619E-05	**.003 ***
F4	PCA 1	98	24.998	4	0.226	5.037E-05	**.005 ***
F4	PCA 2	98	23.933	4	0.214	8.238E-05	**.006 ***
F4	ICA 1	98	30.849	4	0.289	3.286E-06	**3.938E-04***
F4	ICA 4	98	15.031	4	0.119	.005	**.069 ***
C3	PCA 1	97	21.079	4	0.186	3.054E-04	**.014 ***
C3	ICA 3	97	21.478	4	0.190	2.545E-04	**.008 ***
C3	ICA 5	97	19.423	4	0.168	0.001	**.016 ***
C4	PCA 1	97	22.004	4	0.196	2.001E-04	**.011 ***
C4	PCA 2	97	18.596	4	0.159	9.432E-04	**.032 ***
O1	PCA 1	98	18.319	4	0.154	1.069E-03	**.032 ***
O1	ICA 2	98	19.002	4	0.161	.001	**.016 ***
O2	PCA 1	95	25.638	4	0.240	3.744E-05	**.005 ***
O2	ICA 5	95	17.696	4	0.152	.001	**.024 ***

Notes: * (in bold) denotes statistically significant differences for adjusted α-values after the Benjamini-Yekutieli procedure, * $\alpha \leq .1$. Please refer to **Table S1** for detailed information about the groups.

Abbreviations: **[C3, C4, F3, F4, O1, O2],** channels' codes in the standard 10-20% electroencephalography montage; **DF,** degree of freedom; **H**, Kruskal-Wallis H test statistics; **ICA**, independent component analysis; **NREM,** non-rapid eye movement stages; **p**, p-value; **PCA**, principal component analysis; **REM,** rapid eye movement sleep; **SO,** slow oscillations; **Total N**, number of subjects; **α,** α-value; **η2**, effect size.

Table S3. Summary of the two-tailed Mann–Whitney pairwise tests between Control group and each of patients' groups (NREM + REM sleep stages together, channel F3, PCA and ICA projections of SO-power histogram datasets)

Patient group	Control				Patient group				Mann-Whitney statistics				
	Mean	SE	95% CI lower	95% CI upper	Mean	SE	95% CI lower	95% CI upper	U	z	η2	p	adj p
**PCA component 1**													
**NREMP**	**12.934**	**5.324**	**2.135**	**23.732**	**-26.259**	**7.148**	**-41.496**	**-11.023**	**488.000**	**3.720**	**0.261**	**2.071E-04**	**.001***
**NT**					**-27.719**	**9.162**	**-47.247**	**-8.190**	**472.000**	**3.410**	**0.219**	**.001**	**.003***
iRBD					30.557	12.642	3.757	57.357	259.000	-1.034	0.020	.306	1.000
FM					-12.215	13.886	-43.155	18.725	272.000	1.680	0.059	.095	.381
**PCA component 2**													
**NREMP**	**16.557**	**5.204**	**6.003**	**27.111**	**-20.091**	**10.788**	**-43.085**	**2.903**	**458.000**	**3.139**	**0.186**	**.002**	**.007***
NT					2.881	9.496	-17.360	23.121	340.000	0.852	0.014	.399	1.000
**iRBD**					**-25.176**	**10.315**	**-47.044**	**-3.309**	**512.000**	**3.678**	**0.251**	**2.435E-04**	**.001***
FM					8.249	9.191	-12.230	28.728	218.000	0.356	0.003	.731	1.000
**ICA component 2**													
**NREMP**	**0.186**	**0.140**	**-0.099**	**0.471**	**-0.539**	**0.214**	**-0.994**	**-0.083**	**430.000**	**2.596**	**0.127**	**.010**	**.039***
**NT**					**-0.716**	**0.183**	**-1.106**	**-0.327**	**463.000**	**3.236**	**0.198**	**.001**	**.005***
iRBD					0.817	0.267	0.252	1.383	214.000	-1.872	0.065	.063	.250
FM					-0.064	0.249	-0.619	0.490	231.000	0.675	0.009	.508	1.000
**ICA component 5**													
**NREMP**	**0.551**	**0.134**	**0.279**	**0.823**	**-0.431**	**0.183**	**-0.822**	**-0.041**	**502.000**	**3.991**	**0.301**	**6.849E-05**	**2.739E-04***
**NT**					**-0.221**	**0.242**	**-0.737**	**0.295**	**440.000**	**2.790**	**0.147**	**.005**	**.022***
**iRBD**					**-0.497**	**0.224**	**-0.972**	**-0.023**	**525.000**	**3.920**	**0.285**	**9.186E-05**	**3.674E-04***
FM					-0.136	0.403	-1.035	0.762	290.000	2.122	0.094	.035	.140

Notes: pairwise comparisons were performed only for PCA/ICA components for those with significant differences adjusted in the Kruskal–Wallis test between all 5 groups together (**Table S2**).

* (in bold) denotes statistically significant differences for adjusted p-values after the Bonferroni correction, p ≤ .05. Please refer to **Table S1** for detailed information about the groups.

Abbreviations: **adj p,** Bonferroni-adjusted p-value**; F3,** channel's code in the standard 10-20% electroencephalography montage; **FM,** fibromyalgia; **ICA**, independent component analysis; **NREM,** non-rapid eye movement stages; **NREMP**, non-REM parasomnia; **NT1,** narcolepsy; **p**, p-value; **PCA**, principal component analysis; **iRBD,** idiopathic/isolated rapid eye movement sleep behavior disorder; **REM,** rapid eye movement sleep stage; **SE,** standard error of mean; **SO,** slow oscillations; **U**, Mann–Whitney U test statistics; **z,** z-score; **η2,** effect size; **95% CI lower/upper**, 95% confidence interval lower/upper.

Table S4. Summary of the two-tailed Mann–Whitney pairwise tests between Control group and each of patients' groups (NREM + REM sleep stages together, channel F4, PCA and ICA projections of SO-power histogram datasets)

Patient group	Control				Patient group				Mann-Whitney statistics				
	Mean	SE	95% CI lower	95% CI upper	Mean	SE	95% CI lower	95% CI upper	U	z	η2	p	adj p
**PCA component 1**													
NREMP	10.190	5.638	-1.233	21.613	-11.858	6.949	-26.669	2.953	417.000	2.141	0.085	.033	.132
**NT1**					**-27.898**	**9.256**	**-47.626**	**-8.170**	**481.000**	**3.353**	**0.208**	**.001**	**.003***
iRBD					29.972	10.034	8.700	51.243	230.000	-1.694	0.052	.092	.368
FM					-23.697	13.552	-53.892	6.499	306.000	2.324	0.110	.021	.083
**PCA component 2**													
**NREMP**	**19.486**	**4.944**	**9.468**	**29.504**	**-32.638**	**9.643**	**-53.191**	**-12.084**	**533.000**	**4.338**	**0.348**	**1.501E-05**	**6.003E-05***
NT1					-0.202	8.939	-19.255	18.852	390.000	1.629	0.049	.105	.421
**iRBD**					**-14.712**	**11.253**	**-38.567**	**9.142**	**502.000**	**3.260**	**0.193**	**.001**	**.005***
FM					3.189	8.870	-16.574	22.952	274.000	1.558	0.050	.122	.489
**ICA component 1**													
**NREMP**	**0.684**	**0.122**	**0.437**	**0.931**	**-0.381**	**0.254**	**-0.922**	**0.160**	**485.000**	**3.429**	**0.218**	**.001**	**.003***
**NT1**					**-0.198**	**0.243**	**-0.715**	**0.319**	**459.000**	**2.936**	**0.160**	**.003**	**.014***
**iRBD**					**-0.503**	**0.170**	**-0.863**	**-0.143**	**566.000**	**4.426**	**0.356**	**1.002E-05**	**4.010E-05***
**FM**					**-0.744**	**0.277**	**-1.362**	**-0.126**	**367.000**	**3.786**	**0.293**	**.000**	**.001***
**ICA component 4**													
NREMP	-0.040	0.156	-0.356	0.276	0.325	0.236	-0.178	0.828	239.000	-1.231	0.028	.222	.887
NT1					-0.515	0.246	-1.039	0.010	392.000	1.667	0.051	.097	.390
iRBD					0.604	0.222	0.133	1.075	202.000	-2.204	0.088	.028	.113
FM					-0.520	0.257	-1.092	0.053	280.000	1.701	0.059	.091	.365

Notes: pairwise comparisons were performed only for PCA/ICA components for those with significant differences adjusted in the Kruskal–Wallis test between all 5 groups together (**Table S2**).

* (in bold) denotes statistically significant differences for adjusted p-values after the Bonferroni correction, p ≤ .05. Please refer to **Table S1** for detailed information about the groups.

Abbreviations: **adj p,** Bonferroni-adjusted p-value**; F4,** channel's code in the standard 10-20% electroencephalography montage; **FM,** fibromyalgia; **ICA**, independent component analysis; **NREM,** non-rapid eye movement stages; **NREMP**, non-REM parasomnia; **NT1,** narcolepsy type 1; **p**, p-value; **PCA**, principal component analysis; **iRBD,** idiopathic/isolated rapid eye movement sleep behavior disorder; **REM,** rapid eye movement sleep stage; **SE,** standard error of mean; **SO,** slow oscillations; **U**, Mann–Whitney U test statistics; **z,** z-score; **η2,** effect size; **95% CI lower/upper**, 95% confidence interval lower/upper.

Table S5. Summary of the two-tailed Mann–Whitney pairwise tests between Control group and each of patients' groups (NREM + REM sleep stages together, channel C3, PCA and ICA projections of SO-power histogram datasets)

Patient group	Control				Patient group				Mann-Whitney statistics				
	Mean	SE	95% CI lower	95% CI upper	Mean	SE	95% CI lower	95% CI upper	U	z	η2	p	adj p
**PCA component 1**													
NREMP	-3.410	5.878	-15.309	8.490	12.034	10.044	-9.374	33.441	245.000	-1.242	0.028	.218	.871
NT1					28.216	11.418	3.727	52.705	166.000	-2.443	0.111	.015	.060
**iRBD**					**-40.090**	**9.191**	**-59.573**	**-20.606**	**492.000**	**2.860**	**0.146**	**.004**	**.017***
FM					19.872	12.234	-7.804	47.548	133.000	-1.538	0.048	.127	.508
**ICA component 3**													
NREMP	-0.064	0.141	-0.348	0.221	0.331	0.248	-0.199	0.860	230.000	-1.520	0.042	.131	.524
NT1					0.590	0.271	0.009	1.172	178.000	-2.211	0.091	.028	.111
**iRBD**					**-0.890**	**0.187**	**-1.287**	**-0.494**	**502.000**	**3.038**	**0.165**	**.002**	**.010***
FM					0.348	0.261	-0.243	0.938	143.000	-1.290	0.034	.201	.806
**ICA component 5**													
**NREMP**	**0.471**	**0.111**	**0.247**	**0.696**	**-0.264**	**0.208**	**-0.707**	**0.179**	**489.000**	**3.280**	**0.196**	**.001**	**.004***
**NT1**					**-0.324**	**0.236**	**-0.830**	**0.182**	**449.000**	**3.022**	**0.169**	**.003**	**.010***
**iRBD**					**-0.591**	**0.319**	**-1.266**	**0.084**	**514.000**	**3.252**	**0.189**	**0.001**	**.005***
FM					0.075	0.361	-0.741	0.891	265.000	1.736	0.062	.085	.339

Notes: pairwise comparisons were performed only for PCA/ICA components for those with significant differences adjusted in the Kruskal–Wallis test between all 5 groups together (**Table S2**).

* (in bold) denotes statistically significant differences for adjusted p-values after the Bonferroni correction, p ≤ .05. Please refer to **Table S1** for detailed information about the groups.

Abbreviations: **adj p,** Bonferroni-adjusted p-value**;** **C3,** channel's code in the standard 10-20% electroencephalography montage; **FM,** fibromyalgia; **ICA**, independent component analysis; **NREM,** non-rapid eye movement stages; **NREMP**, non-REM parasomnia; **NT1,** narcolepsy type 1; **p**, p-value; **PCA**, principal component analysis; **iRBD,** idiopathic/isolated rapid eye movement sleep behavior disorder; **REM,** rapid eye movement sleep stage; **SE,** standard error of mean; **SO,** slow oscillations; **U**, Mann–Whitney U test statistics; **z,** z-score; **η2,** effect size; **95% CI lower/upper**, 95% confidence interval lower/upper.

Table S6. Summary of the two-tailed Mann–Whitney pairwise tests between Control group and each of patients' groups (NREM + REM sleep stages together, channel C4, PCA and ICA projections of SO-power histogram datasets)

Patient group	Control				Patient group				Mann-Whitney statistics				
	Mean	SE	95% CI lower	95% CI upper	Mean	SE	95% CI lower	95% CI upper	U	z	η2	p	adj p
**PCA component 1**													
**NREMP**	**9.671**	**5.334**	**-1.127**	**20.468**	**-18.839**	**9.607**	**-39.444**	**1.765**	**423.000**	**2.520**	**0.118**	**.012**	**.048***
**NT1**					**-30.384**	**10.236**	**-52.339**	**-8.430**	**464.000**	**3.312**	**0.203**	**.001**	**.004***
iRBD					31.401	11.774	6.440	56.361	242.000	-1.595	0.045	.113	.451
FM					-15.692	12.304	-43.107	11.723	304.000	2.096	0.088	.037	.149
**PCA component 2**													
**NREMP**	**15.758**	**3.786**	**8.093**	**23.424**	**-20.644**	**7.848**	**-37.477**	**-3.811**	**488.000**	**3.776**	**0.264**	**.000**	**.001***
NT1					-5.585	13.432	-34.392	23.223	372.000	1.535	0.044	.127	.508
**iRBD**					**-20.648**	**11.160**	**-44.307**	**3.011**	**513.000**	**3.234**	**0.187**	**.001**	**.005***
FM					11.807	9.110	-8.491	32.105	223.000	0.199	0.001	.851	1.000

Notes: pairwise comparisons were performed only for PCA/ICA components for those with significant differences adjusted in the Kruskal–Wallis test between all 5 groups together (**Table S2**).

* (in bold) denotes statistically significant differences for adjusted p-values after the Bonferroni correction, p ≤ .05. Please refer to **Table S1** for detailed information about the groups.

Abbreviations: **adj p,** Bonferroni-adjusted p-value**; C4,** channel's code in the standard 10-20% electroencephalography montage; **FM,** fibromyalgia; **ICA**, independent component analysis; **NREM,** non-rapid eye movement stages; **NREMP**, non-REM parasomnia; **NT1,** narcolepsy type 1; **p**, p-value; **PCA**, principal component analysis; **iRBD,** idiopathic/isolated rapid eye movement sleep behavior disorder; **REM,** rapid eye movement sleep stage; **SE,** standard error of mean; **SO,** slow oscillations; **U**, Mann–Whitney U test statistics; **z,** z-score; **η2,** effect size; **95% CI lower/upper**, 95% confidence interval lower/upper.

Table S7. Summary of the two-tailed Mann–Whitney pairwise tests between Control group and each of patients' groups (NREM + REM sleep stages together, channel O1, PCA and ICA projections of SO-power histogram datasets)

Patient group	Control				Patient group				Mann-Whitney statistics				
	Mean	SE	95% CI lower	95% CI upper	Mean	SE	95% CI lower	95% CI upper	U	z	η2	p	adj p
**PCA component 1**													
NREMP	9.875	5.270	-0.803	20.554	-14.605	11.975	-40.129	10.919	416.000	2.122	0.083	.035	.139
**NT1**					**-19.317**	**8.115**	**-36.614**	**-2.020**	**445.000**	**2.671**	**0.132**	**.008**	**.031***
iRBD					25.814	6.586	11.852	39.775	225.000	-1.785	0.058	.076	.303
FM					-24.667	15.973	-60.258	10.924	289.000	1.917	0.075	.057	.227
**ICA component 2**													
**NREMP**	**0.523**	**0.117**	**0.286**	**0.760**	**-0.333**	**0.247**	**-0.859**	**0.193**	**461.000**	**2.974**	**0.164**	**.003**	**.012***
**NT1**					**-0.735**	**0.267**	**-1.304**	**-0.167**	**500.000**	**3.713**	**0.255**	**2.127E-04**	**.001***
iRBD					0.002	0.224	-0.473	0.476	428.000	1.912	0.066	.057	.228
FM					-0.256	0.329	-0.988	0.476	293.000	2.013	0.083	.045	.182

Notes: pairwise comparisons were performed only for PCA/ICA components for those with significant differences adjusted in the Kruskal–Wallis test between all 5 groups together (**Table S2**).

* (in bold) denotes statistically significant differences for adjusted p-values after the Bonferroni correction, p ≤ .05. Please refer to **Table S1** for detailed information about the groups.

Abbreviations: **adj p,** Bonferroni-adjusted p-value**; FM,** fibromyalgia; **ICA**, independent component analysis; **NREM,** non-rapid eye movement stages; **NREMP**, non-REM parasomnia; **NT1,** narcolepsy type 1; **p**, p-value; **O1,** channel's code in the standard 10-20% electroencephalography montage; **PCA**, principal component analysis; **iRBD,** idiopathic/isolated rapid eye movement sleep behavior disorder; REM, rapid eye movement sleep stage; **SE,** standard error of mean; **SO,** slow oscillations; **U**, Mann–Whitney U test statistics; **z,** z-score; **η2,** effect size; **95% CI lower/upper**, 95% confidence interval lower/upper.

Table S8. Summary of the two-tailed Mann–Whitney pairwise tests between Control group and each of patients' groups (NREM + REM sleep stages together, channel O2, PCA and ICA projections of SO-power histogram datasets)

Patient group	Control				Patient group				Mann-Whitney statistics				
	**Mean**	**SE**	**95% CI lower**	**95% CI upper**	**Mean**	**SE**	**95% CI lower**	**95% CI upper**	**U**	**z**	**η2**	**p**	**adj p**
**PCA component 1**													
NREMP	10.126	5.205	-0.419	20.671	-10.044	9.865	-31.355	11.268	359.000	1.919	0.071	.056	.225
**NT1**					**-27.809**	**7.521**	**-43.840**	**-11.779**	**494.000**	**3.599**	**0.240**	**3.310E-04**	**.001***
iIRBD					31.668	10.038	10.273	53.063	225.000	-1.497	0.041	.137	.548
**FM**					**-27.811**	**12.767**	**-56.257**	**0.635**	**321.000**	**2.684**	**0.147**	**.008**	**.030***
**ICA component 5**													
**NREMP**	**0.368**	**0.115**	**0.134**	**0.601**	**-0.262**	**0.155**	**-0.598**	**0.074**	**400.000**	**2.764**	**0.147**	**.006**	**.024***
**NT1**					**-0.732**	**0.255**	**-1.276**	**-0.189**	**489.000**	**3.505**	**0.227**	**4.740E-04**	**.002***
iRBD					0.370	0.306	-0.282	1.023	307.000	0.057	0.000	.962	1.000
FM					-0.411	0.371	-1.237	0.416	294.000	2.037	0.085	.043	.172

Notes: pairwise comparisons were performed only for PCA/ICA components for those with significant differences adjusted in the Kruskal–Wallis test between all 5 groups together (**Table S2**).

* (in bold) denotes statistically significant differences for adjusted p-values after the Bonferroni correction, p ≤ .05. Please refer to **Table S1**for detailed information about the groups.

Abbreviations: **adj p,** Bonferroni-adjusted p-value**; FM,** fibromyalgia; **ICA**, independent component analysis; **NREM,** non-rapid eye movement stages; **NREMP**, non-REM parasomnia; **NT1** narcolepsy type 1; **p**, p-value; **O2,** channel's code in the standard 10-20% electroencephalography montage; **PCA**, principal component analysis; **iRBD,** idiopathic/isolated rapid eye movement sleep behaviour disorder; REM, rapid eye movement sleep stage; **SE,** standard error of mean; **SO,** slow oscillations; **U**, Mann–Whitney U test statistics; **z,** z-score; **η2,** effect size; **95% CI lower/upper**, 95% confidence interval lower/upper.

Table S9. Summary of Kruskal–Wallis test between five groups (PCA and ICA components of SO-power histogram datasets; NREM sleep stages only; components with significant differences only)

Channel	Component	Total N	H	DF	η2	p	α
F3	PCA 1	97	18.185	4	0.154	.001	**.064**
F3	ICA 2	97	18.898	4	0.162	.001	**.025**
F4	PCA 1	98	20.148	4	0.174	4.700E-04	**.044**
F4	PCA 2	98	24.558	4	0.221	6.000E-05	**.017**
F4	PCA 4	98	20.749	4	0.180	3.600E-04	**.044**
F4	ICA 3	98	19.833	4	0.170	.001	**.025**
C3	PCA 1	97	18.312	4	0.156	.001	**.064**
C3	ICA 4	97	17.238	4	0.144	.002	**.039**
O1	ICA 2	98	19.146	4	0.163	.001	**.025**

Notes: * (in bold) denotes statistically significant differences for adjusted α-values after the Benjamini-Yekutieli procedure, * $\alpha \leq .1$. There were no significant differences for C4 and O2 channels ($\alpha > .1$ in all the cases). Please refer to **Table S1** for detailed information about the groups.

Abbreviations: **[C3, C4, F3, F4, O1, O2],** channels' codes in the standard 10-20% electroencephalography montage; **DF,** degree of freedom; **H**, Kruskal-Wallis H test statistics; **ICA**, independent component analysis; **NREM,** non-rapid eye movement stages; **p**, p-value; **PCA**, principal component analysis; **REM,** rapid eye movement sleep stage; **SO,** slow oscillations; **Total N**, number of subjects; **α,** α-value; **η2**, effect size.

Table S10. Summary of the two-tailed Mann–Whitney pairwise tests between Control group and each of patients' groups (NREM sleep stages only, channel F3, PCA and ICA projections of SO-power histogram datasets)

Patient group	Control				Patient group				Mann-Whitney statistics				
	Mean	SE	95% CI lower	95% CI upper	Mean	SE	95% CI lower	95% CI upper	U	z	η2	p	adj p
**PCA component 1**													
NREMP	5.126	6.344	-7.741	17.992	-13.472	7.675	-29.831	2.887	367.000	1.376	0.036	.172	.688
NT1					-28.843	10.219	-50.623	-7.063	415.000	2.306	0.100	.022	.087
iRBD					36.993	11.209	13.232	60.755	187.000	-2.375	0.104	.018	.072
FM					-12.863	13.108	-42.069	16.343	241.000	0.920	0.018	.364	1.000
**ICA component 2**													
NREMP	0.348	0.136	0.073	0.623	-0.533	0.285	-1.141	0.075	407.000	2.151	0.087	.032	.129
NT1					0.116	0.185	-0.278	0.509	341.000	0.872	0.014	.389	1.000
**iRBD**					**-0.687**	**0.152**	**-1.008**	**-0.365**	**536.000**	**4.125**	**0.315**	**3.855E-05**	**1.542E-04***
FM					0.498	0.411	-0.417	1.412	206.000	0.061	0.000	.961	1.000

Notes: pairwise comparisons were performed only for PCA/ICA components for those with significant differences adjusted in the Kruskal–Wallis test between all 5 groups together (**Table S9**).

* (in bold) denotes statistically significant differences for adjusted p-values after the Bonferroni correction, p ≤ .05. Please refer to **Table S1** for detailed information about the groups.

Abbreviations: **adj p,** Bonferroni-adjusted p-value**; F3,** channel's code in the standard 10-20% electroencephalography montage; **FM,** fibromyalgia; **ICA**, independent component analysis; **NREM,** non-rapid eye movement stages; **NREMP**, non-REM parasomnia; **NT1,** narcolepsy type 1; **p**, p-value; **PCA**, principal component analysis; **iRBD,** idiopathic/isolated rapid eye movement sleep behavior disorder; **REM,** rapid eye movement sleep stage; **SE,** standard error of mean; **SO,** slow oscillations; **U**, Mann–Whitney U test statistics; **z,** z-score; **η2,** effect size; **95% CI lower/upper**, 95% confidence interval lower/upper.

Table S11. Summary of the two-tailed Mann–Whitney pairwise tests between Control group and each of patients' groups (NREM sleep stages only, channel F4, PCA and ICA projections of SO-power histogram datasets)

Patient group	Control				Patient group				Mann-Whitney statistics				
	**Mean**	**SE**	**95% CI lower**	**95% CI upper**	**Mean**	**SE**	**95% CI lower**	**95% CI upper**	**U**	**z**	**η2**	**p**	**adj p**
**PCA component 1**													
NREMP	-2.769	6.490	-15.918	10.380	-4.870	8.768	-23.559	13.820	321.000	0.322	0.002	.755	1.000
NT1					29.327	9.971	8.075	50.579	174.000	-2.463	0.112	.014	.057
**iRBD**					**-32.134**	**8.521**	**-50.197**	**-14.071**	**461.000**	**2.513**	**0.115**	**.012**	**.049***
FM					23.654	13.224	-5.812	53.119	151.000	-1.390	0.039	.168	.673
**PCA component 2**													
**NREMP**	**16.678**	**3.911**	**8.754**	**24.602**	**-35.347**	**8.131**	**-52.678**	**-18.015**	**556.000**	**4.774**	**0.422**	**1.895E-06**	**7.582E-06***
**NT**					**-8.074**	**9.627**	**-28.593**	**12.446**	**444.000**	**2.652**	**0.130**	**.008**	**.033***
iRBD					1.482	11.524	-22.948	25.911	423.000	1.821	0.060	.070	.280
FM					3.252	8.763	-16.273	22.777	259.000	1.198	0.029	.236	.942
**PCA component 4**													
NREMP	16.183	4.084	7.907	24.459	-4.239	6.325	-17.720	9.242	433.000	2.444	0.111	.015	.060
**NT1**					**-5.998**	**5.229**	**-17.143**	**5.147**	**449.000**	**2.747**	**0.140**	**.006**	**.025***
**iRBD**					**-18.964**	**5.623**	**-30.884**	**-7.043**	**543.000**	**4.007**	**0.292**	**6.395E-05**	**2.558E-04***
FM					-11.707	12.248	-38.999	15.584	301.000	2.204	0.099	.028	.113
**ICA component 3**													
**NREMP**	**0.333**	**0.144**	**0.042**	**0.625**	**-0.523**	**0.257**	**-1.070**	**0.025**	**437.000**	**2.519**	**0.118**	**.012**	**.048***
NT1					0.437	0.255	-0.107	0.981	284.000	-0.379	0.003	.712	1.000
**iRBD**					**-0.777**	**0.203**	**-1.207**	**-0.348**	**524.000**	**3.661**	**0.244**	**2.605E-04**	**.001***
FM					0.175	0.191	-0.249	0.600	228.000	0.455	0.004	.658	1.000

Notes: pairwise comparisons were performed only for PCA/ICA components for those with significant differences adjusted in the Kruskal–Wallis test between all 5 groups together (**Table S9**).

* (in bold) denotes statistically significant differences for adjusted p-values after the Bonferroni correction, $p \leq .05$. Please refer to **Table S1** for detailed information about the groups.

Abbreviations: **adj p,** Bonferroni-adjusted p-value**;** **F4,** channel's code in the standard 10-20% electroencephalography montage; **FM,** fibromyalgia; **ICA**, independent component analysis; **NREM,** non-rapid eye movement stages; **NREMP**, non-REM parasomnia; **NT1,** narcolepsy type 1; **p**, p-value; **PCA**, principal component analysis; **iRBD,** idiopathic/isolated rapid eye movement sleep behavior disorder; **REM,** rapid eye movement sleep stage; **SE,** standard error of mean; **SO,** slow oscillations; **U**, Mann–Whitney U test statistics; **z,** z-score; **η2,** effect size; **95% CI lower/upper**, 95% confidence interval lower/upper.

Table S12. Summary of the two-tailed Mann–Whitney pairwise tests between Control group and each of patients' groups (NREM sleep stages only, channel C3, PCA and ICA projections of SO-power histogram datasets)

Patient group	Control				Patient group				Mann-Whitney statistics				
	Mean	SE	95% CI lower	95% CI upper	Mean	SE	95% CI lower	95% CI upper	U	z	η2	p	adj p
**PCA component 1**													
NREMP	-2.723	6.292	-15.460	10.014	11.247	10.315	-10.740	33.234	254.000	-1.075	0.021	.287	1.000
NT1					26.380	12.074	0.485	52.276	179.000	-2.192	0.089	.029	.116
**iRBD**					**-38.305**	**9.035**	**-57.459**	**-19.152**	**483.000**	**2.700**	**0.130**	**.007**	**.029***
FM					18.171	13.022	-11.286	47.629	141.000	-1.340	0.037	.184	.738
**ICA component 4**													
NREMP	-0.042	0.143	-0.331	0.248	0.357	0.236	-0.145	0.859	236.000	-1.408	0.036	.162	.647
NT1					0.526	0.293	-0.103	1.155	197.000	-1.844	0.063	.067	.266
**iRBD**					**-0.840**	**0.183**	**-1.229**	**-0.452**	**490.000**	**2.824**	**0.142**	**.005**	**.019***
FM					0.231	0.287	-0.418	0.880	155.000	-0.992	0.020	.327	1.000

Notes: pairwise comparisons were performed only for PCA/ICA components for those with significant differences adjusted in the Kruskal–Wallis test between all 5 groups together (**Table S9**).

* (in bold) denotes statistically significant differences for adjusted p-values after the Bonferroni correction, $p \leq .05$. Please refer to **Table S1** for detailed information about the groups.

Abbreviations: **adj p,** Bonferroni-adjusted p-value**;** **C3,** channel's code in the standard 10-20% electroencephalography montage; **FM,** fibromyalgia; **ICA**, independent component analysis; **NREM,** non-rapid eye movement stages; **NREMP**, non-REM parasomnia; **NT1,** narcolepsy type 1; **p**, p-value; **PCA**, principal component analysis; **iRBD,** idiopathic/isolated rapid eye movement sleep behavior disorder; **REM,** rapid eye movement sleep stage; **SE,** standard error of mean; **SO,** slow oscillations; **U**, Mann–Whitney U test statistics; **z,** z-score; **η2,** effect size; **95% CI lower/upper**, 95% confidence interval lower/upper.

Table S13. Summary of the two-tailed Mann–Whitney pairwise tests between Control group and each of patients' groups (NREM sleep stages only, channel O1, PCA and ICA projections of SO-power histogram datasets)

Patient group	Control				Patient group				Mann-Whitney statistics				
	Mean	SE	95% CI lower	95% CI upper	Mean	SE	95% CI lower	95% CI upper	U	z	η2	p	adj p
**ICA component 2**													
**NREMP**	**0.532**	**0.113**	**0.303**	**0.760**	**-0.309**	**0.239**	**-0.819**	**0.201**	**454.000**	**2.842**	**0.150**	**.005**	**.019***
**NT1**					**-0.638**	**0.282**	**-1.241**	**-0.036**	**506.000**	**3.827**	**0.271**	**1.350E-04**	**.001***
**iRBD**					**-0.179**	**0.236**	**-0.680**	**0.321**	**462.000**	**2.532**	**0.117**	**.012**	**.047***
FM					-0.180	0.336	-0.929	0.568	292.000	1.989	0.081	.048	.192

Notes: pairwise comparisons were performed only for PCA/ICA components for those with significant differences adjusted in the Kruskal–Wallis test between all 5 groups together (**Table S9**).

* (in bold) denotes statistically significant differences for adjusted p-values after the Bonferroni correction, p ≤ .05. Please refer to **Table S1** for detailed information about the groups.

Abbreviations: **adj p,** Bonferroni-adjusted p-value**; FM,** fibromyalgia; **ICA**, independent component analysis; **NREM,** non-rapid eye movement stages; **NREMP**, non-REM parasomnia; **NT1,** narcolepsy type 1; **O1,** channel's code in the standard 10-20% electroencephalography montage; **p**, p-value; **PCA**, principal component analysis; **iRBD,** idiopathic/isolated rapid eye movement sleep behavior disorder; **REM,** rapid eye movement sleep stage; **SE,** standard error of mean; **SO,** slow oscillations; **U**, Mann–Whitney U test statistics; **z,** z-score; **η2,** effect size; **95% CI lower/upper**, 95% confidence interval lower/upper.

Table S14. Summary of Kruskal–Wallis test between five groups (PCA and ICA components of SO-power histogram datasets; REM sleep stage only; components with significant differences only)

Channel	Component	Total N	H	DF	η2	p	α
F3	ICA 2	97	18.898	4	0.162	.001	**.025**
C3	PCA 1	97	18.312	4	0.156	.001	**.064**
C3	ICA 4	97	17.238	4	0.144	.002	**.039**

Notes: * (in bold) denotes statistically significant differences for adjusted α-values after the Benjamini-Yekutieli procedure, * α ≤ .1. There were no significant differences for F4, C4, O1, and O2 channels (α>.1 in all the cases). Please refer to **Table S1** for detailed information about the groups.

Abbreviations: [**C3, C4, F3, F4, O1, O2],** channels' codes in the standard 10-20% electroencephalography montage; **DF,** degree of freedom; **H**, Kruskal-Wallis H test statistics; **ICA**, independent component analysis; **NREM,** non-rapid eye movement stages; **p**, p-value; **PCA**, principal component analysis; **REM,** rapid eye movement sleep stage; **SO,** slow oscillations; **Total N**, number of subjects; **α,** α-value; **η2**, effect size.

Table S15. Summary of the two-tailed Mann–Whitney pairwise tests between Control group and each of patients' groups (REM sleep stage only, channel F3, PCA and ICA projections of SO-power histogram datasets)

Patient group	Control				Patient group				Mann-Whitney statistics				
	Mean	SE	95% CI lower	95% CI upper	Mean	SE	95% CI lower	95% CI upper	U	z	η2	p	adj p
**ICA component 5**													
**NREMP**	**0.532**	**0.132**	**0.264**	**0.799**	**-0.312**	**0.228**	**-0.799**	**0.175**	**453.000**	**3.042**	**0.175**	**.002**	**.010***
NT					-0.087	0.211	-0.537	0.363	413.000	2.267	0.097	.024	.096
**iRBD**					**-0.671**	**0.207**	**-1.110**	**-0.232**	**532.000**	**4.051**	**0.304**	**5.311E-05**	**2.124E-04***
FM					-0.190	0.445	-1.195	0.816	243.000	1.508	0.048	.135	.540

Notes: pairwise comparisons were performed only for PCA/ICA components for those with significant differences adjusted in the Kruskal–Wallis test between all 5 groups together (**Table S14**).

* (in bold) denotes statistically significant differences for adjusted p-values after the Bonferroni correction, p ≤ .05. Please refer to **Table S1** for detailed information about the groups.

Abbreviations: **adj p,** Bonferroni-adjusted p-value**;** **F3,** channel's code in the standard 10-20% electroencephalography montage; **FM,** fibromyalgia; **ICA**, independent component analysis; **NREM,** non-rapid eye movement stages; **NREMP**, non-REM parasomnia; **NT1,** narcolepsy type 1; **p**, p-value; **PCA**, principal component analysis; **iRBD,** idiopathic/isolated rapid eye movement sleep behavior disorder; **REM,** rapid eye movement sleep stage; **SE,** standard error of mean; **SO,** slow oscillations; **U**, Mann–Whitney U test statistics; **z,** z-score; **η2,** effect size; **95% CI lower/upper**, 95% confidence interval lower/upper.

Table S16. Summary of the two-tailed Mann–Whitney pairwise tests between Control group and each of patients' groups (REM sleep stage only, channel C3, PCA and ICA projections of SO-power histogram datasets)

Patient group	Control				Patient group				Mann-Whitney statistics				
	**Mean**	**SE**	**95% CI lower**	**95% CI upper**	**Mean**	**SE**	**95% CI lower**	**95% CI upper**	**U**	**z**	**η2**	**p**	**adj p**
**PCA component 4**													
NREMP	7.765	2.734	2.231	13.299	1.444	4.968	-9.145	12.033	401.000	1.649	0.049	.101	.404
NT1					-0.213	7.624	-16.565	16.139	371.000	1.516	0.043	.132	.528
**iRBD**					**-17.179**	**2.699**	**-22.901**	**-11.457**	**591.000**	**4.624**	**0.382**	**3.926E-06**	**1.570E-05***
FM					-3.411	10.209	-26.953	20.130	234.000	1.545	0.050	.126	.502
**ICA component 3**													
NREMP	0.170	0.145	-0.124	0.464	0.092	0.170	-0.270	0.454	333.000	0.389	0.003	.704	1.000
NT1					0.609	0.303	-0.040	1.259	228.000	-1.246	0.029	.216	.866
**iRBD**					**-0.889**	**0.206**	**-1.324**	**-0.453**	**532.000**	**3.573**	**0.228**	**3.654E-04**	**.001***
FM					-0.236	0.295	-0.916	0.444	220.000	1.175	0.029	.245	.981

Notes: pairwise comparisons were performed only for PCA/ICA components for those with significant differences adjusted in the Kruskal–Wallis test between all 5 groups together (**Table S14**).

* (in bold) denotes statistically significant differences for adjusted p-values after the Bonferroni correction, p ≤ .05. Please refer to **Table S1** for detailed information about the groups.

Abbreviations: **adj p,** Bonferroni-adjusted p-value**; C3,** channel's code in the standard 10-20% electroencephalography montage; **FM,** fibromyalgia; **ICA**, independent component analysis; **NREM,** non-rapid eye movement stages; **NREMP**, non-REM parasomnia; **NT,** narcolepsy; **p**, p-value; **PCA**, principal component analysis; **iRBD,** idiopathic/isolated rapid eye movement sleep behavior disorder; **REM,** rapid eye movement sleep stage; **SE,** standard error of mean; **SO,** slow oscillations; **U**, Mann–Whitney U test statistics; **z,** z-score; **η2,** effect size; **95% CI lower/upper**, 95% confidence interval lower/upper.

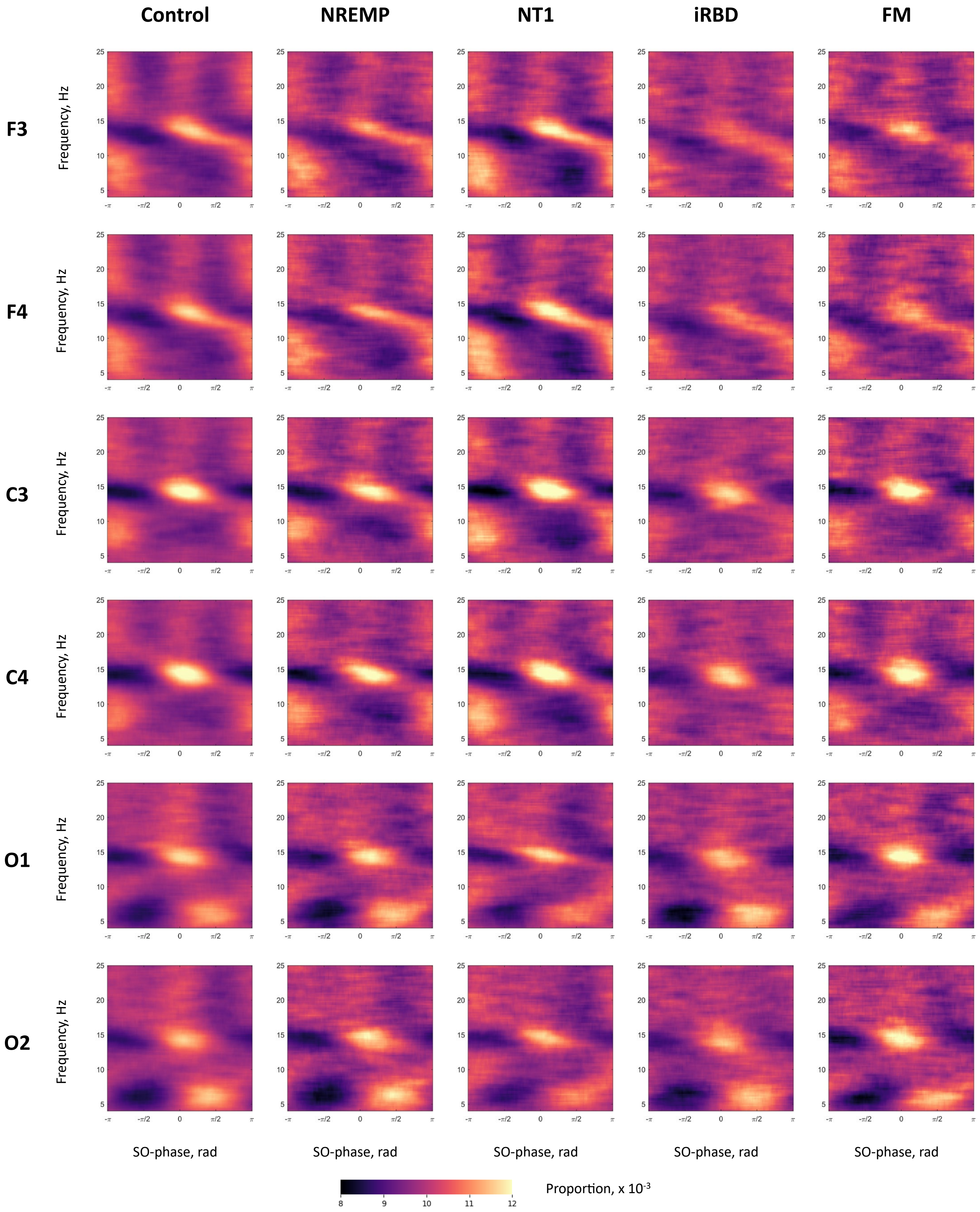

Fig. S6. Group average SO-phase histograms for NREM + REM stages together. Please see **Table S1**for detailed information about the groups.

Abbreviations: [**C3, C4, F3, F4, O1, O2],** channels' codes in the standard 10-20% electroencephalography montage; **FM,** fibromyalgia; **NREM,** non-rapid eye movement stages; **NREMP**, non-REM parasomnia; **NT1**, narcolepsy type 1; **rad,** radian; **iRBD,** idiopathic/isolated rapid eye movement sleep behavior disorder; **REM,** rapid eye movement stage, **SO,** slow oscillations.

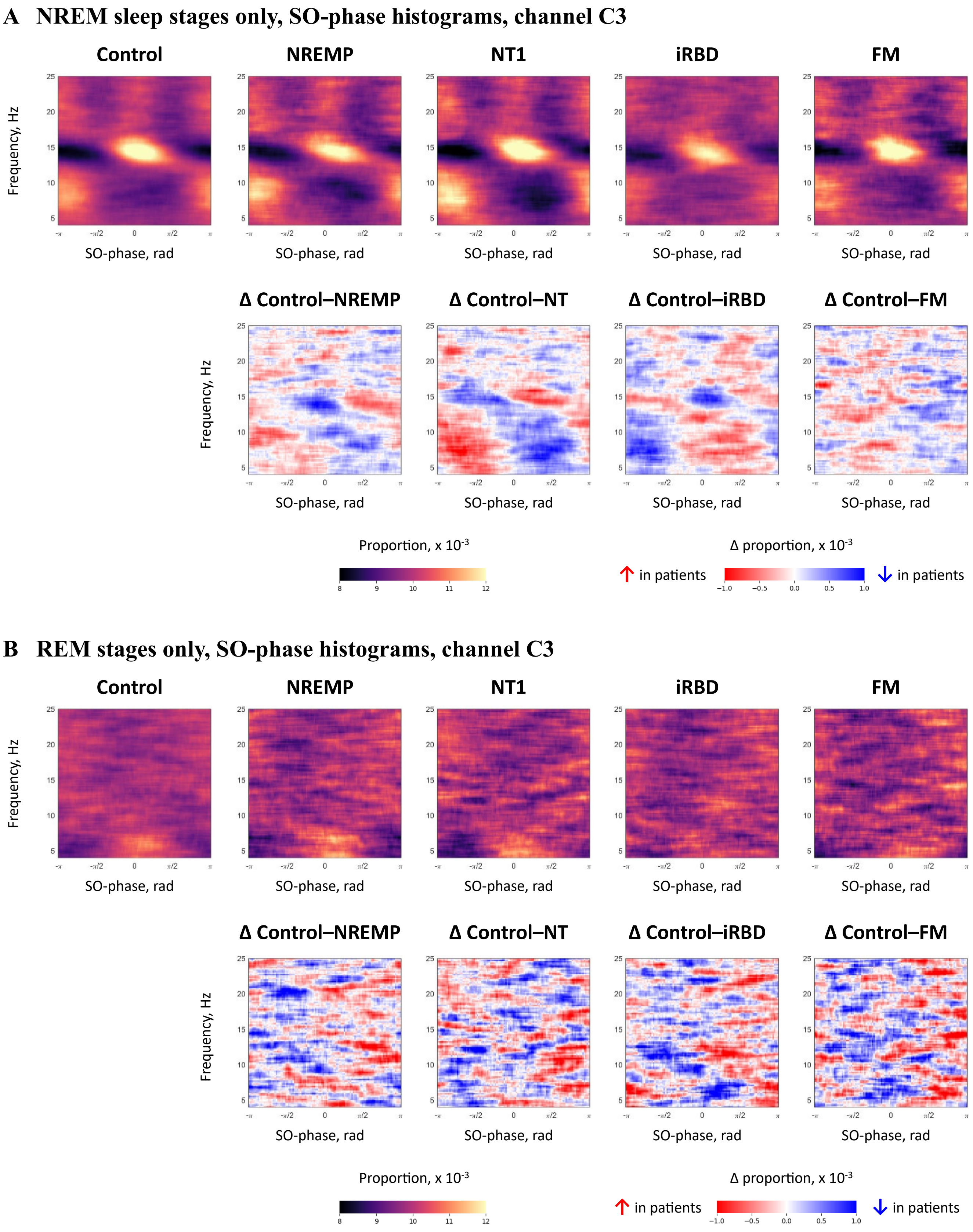

Fig. S7. SO-phase histograms for NREM sleep only and REM only sleep stages (A and B, respectively) at channel C3. In each panel, the upper row of histograms illustrate group average, while the lower row shows arithmetical differences between histogram matrices. Please see **Figure 2** for SO-phase histograms at NREM+REM stages together and **Table S1** for detailed information about the groups.

Abbreviations: **C3,** channels' codes in the standard 10-20% electroencephalography montage; **FM,** fibromyalgia; **Hz,** hertz; **min,** minute; **NREM,** non-rapid eye movement stages; **NREMP**, non-REM parasomnia**;** **NT1**, narcolepsy type 1; **rad,** radian; **iRBD,** idiopathic/isolated rapid eye movement sleep behavior disorder; **REM,** rapid eye movement sleep stage; **SO,** slow oscillations.

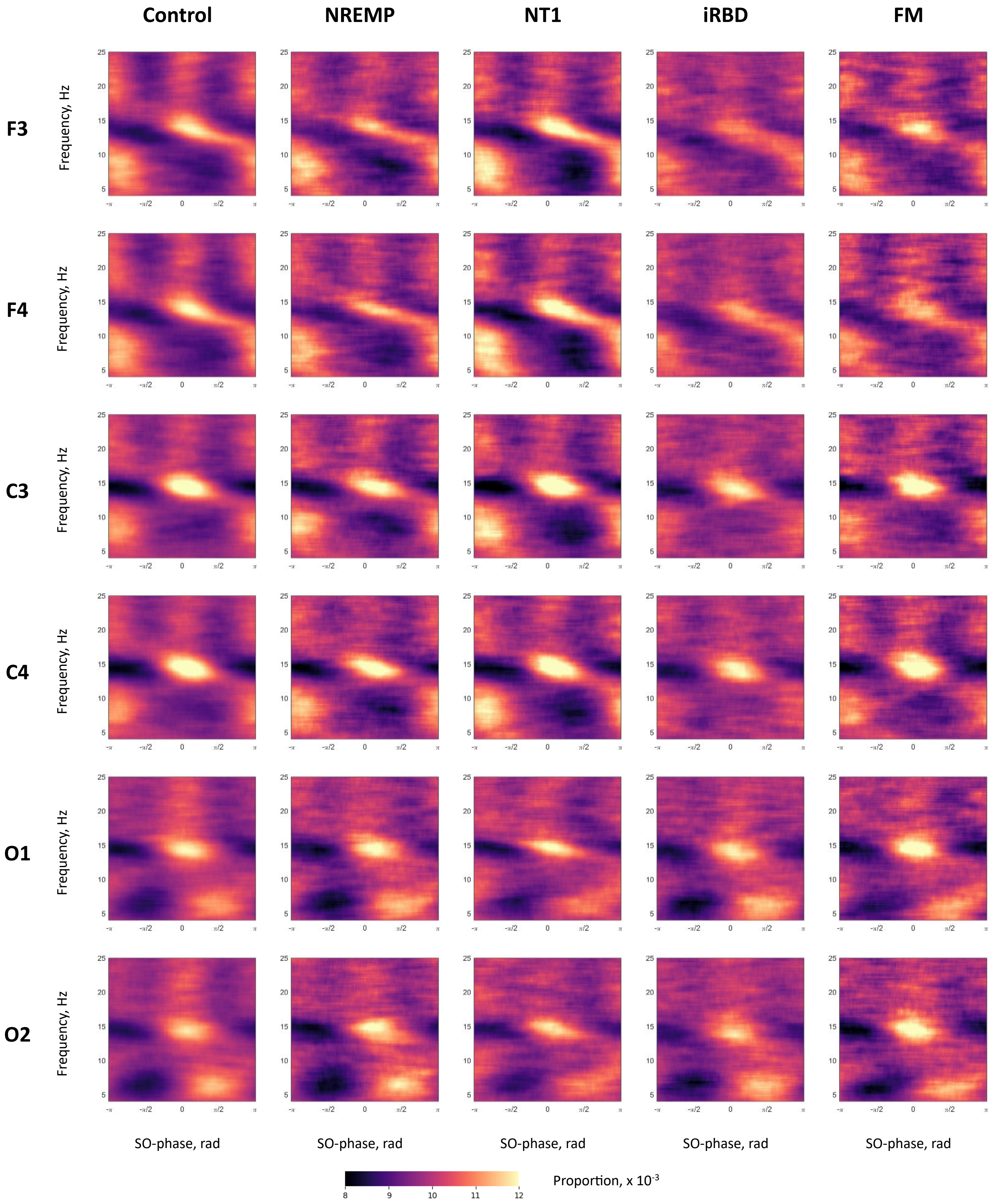

Fig. S8. Group average SO-phase histograms for NREM sleep stages only. Please see **Table S1** for detailed information about the groups.

Abbreviations: **[C3, C4, F3, F4, O1, O2],** channels' codes in the standard 10-20% electroencephalography montage; **FM,** fibromyalgia; **NREM,** non-rapid eye movement stages; **NREMP**, non-REM parasomnia; **NT1**, narcolepsy type 1; **rad,** radian; **iRBD,** idiopathic/isolated rapid eye movement sleep behavior disorder; **SO,** slow oscillations.

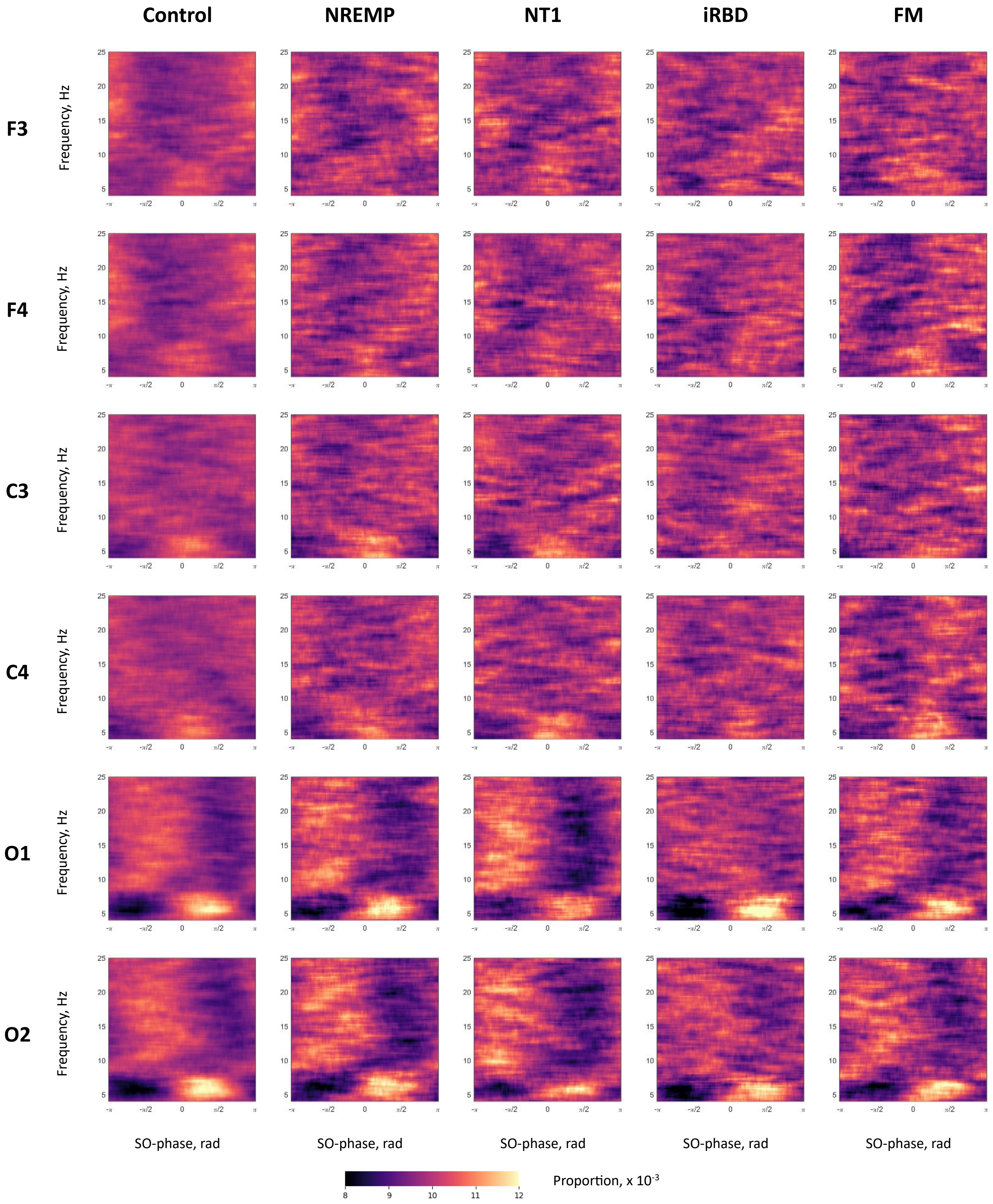

Fig. S9. Group average SO-phase histograms for REM sleep stage only. Please see **Table S1** for detailed information about the groups.

Abbreviations: **[C3, C4, F3, F4, O1, O2],** channels' codes in the standard 10-20% electroencephalography montage; **FM,** fibromyalgia; **NREM,** non-rapid eye movement stages; **NREMP**, non-REM parasomnia; **NT1**, narcolepsy type 1; **rad,** radian; **iRBD,** idiopathic/isolated rapid eye movement sleep behavior disorder; **REM,** rapid eye movement stage, **SO,** slow oscillations.

Patterns of PCA/ICA components with significant differences between Control and patients

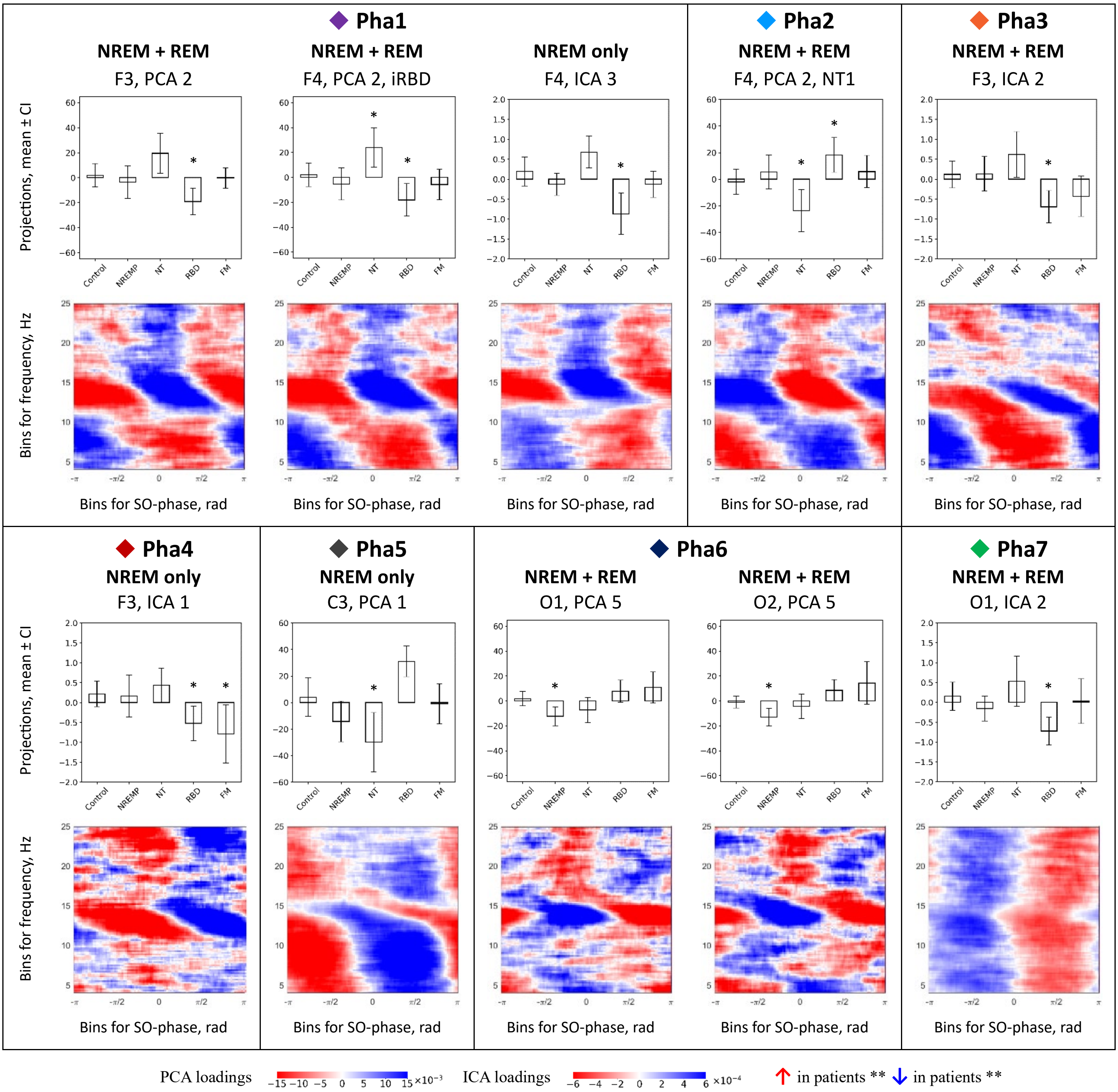

Fig. S10. SO-phase histograms: PCA and ICA components that were associated with significant differences between groups (pairwise comparisons of Control versus each of the patient groups, p≤.05 Bonferroni adjusted). There were no significant differences for SO-phase histograms during REM in any case. Component histograms were classified into categories Pha1-Pha7 based on the visual similarity of the depicted patterns. Please refer to **Fig. S11**, **Tables S17-S28,** and **Table S1** for summary of between-group differences, detailed statistics, and information about the groups, respectively.

* denotes statistically significant Bonferroni-corrected p-values, p ≤ .05.
** red (increase) and blue (decrease) colors indicate these changes in patient groups with significant differences versus Control; all the patterns are oriented in such a way as to unify the interpretation approaches.
Note: Pattern Pha2 F4, PCA 2, NT1 is an inverted version of Pha1 F4, PCA 2, iRBD.

Abbreviations: **[C3, C4, F3, F4, O1, O2],** channel's code in the standard 10-20% electroencephalography montage; **CI,** confidence intervals; **Hz**, hertz; **FM,** fibromyalgia; **ICA,** independent component analysis; **ICA x**, ICA component x; **NREM**, non-rapid eye movement stages; **NREMP**, non-REM parasomnia**; NT1**, narcolepsy type 1; **p,** p-value; **PCA**, principal component analysis; **PCA x,** PCA component x; **Pha1-Pha7**, SO-phase patterns 1-7; **rad,** radian; **iRBD,** idiopathic/isolated rapid eye movement sleep behavior disorder; **REM,** rapid eye movement sleep stage **SO,** slow oscillations.

Summary of significant differences between Control and patient groups, SO phase histograms

NREM + REM	NREMP		NT1		iRBD		FM	
	Left	Right	Left	Right	Left	Right	Left	Right
F3-F4				◆ (Pha2)	◆ (Pha1) ◆ (Pha3)	◆ (Pha1)		
C3-C4								
O1-O2	◆ (Pha6)	◆ (Pha6)			◆ (Pha7)			

NREM only	NREMP		NT1		iRBD		FM	
	Left	Right	Left	Right	Left	Right	Left	Right
F3-F4					◆ (Pha4)	◆ (Pha1)	◆ (Pha4)	
C3-C4			◆ (Pha5)					
O1-O2								

Pattern codes ◆ **Pha1** ◆ **Pha2** ◆ **Pha3** ◆ **Pha4** ◆ **Pha5** ◆ **Pha6** ◆ **Pha7**

Figure S11. Pattern-level summary of significant SO-phase differences between controls and patient groups. Schematic overview of SO-phase patterns (Pha1–Pha7) showing significant differences between controls and patient groups across channels and stages. Each coloured symbol denotes the presence of at least one PCA or ICA component belonging to the corresponding SO-phase pattern (as defined in Figure S10) that differed significantly between controls and the indicated patient group (Bonferroni-adjusted $p \leq 0.05$) in that channel and stage. Symbols are arranged by sleep stage (NREM+REM sleep, NREM-only sleep) and by left/right hemispheric derivations (F3/F4, C3/C4, O1/O2). The figure is intended as an illustrative, pattern-level map of where phase-coupled TF-peak alterations occur, rather than an exhaustive enumeration of all significant components. A complete quantitative summary of the number of significant SO-phase components per group and stage is provided in Supplementary Figure S19 and in Supplementary Tables S17–S23 (NREM+REM sleep) and S24–S28 (NREM-only sleep).

Abbreviations: **[C3, C4, F3, F4, O1, O2],** channel's code in the standard 10-20% electroencephalography montage; **FM,** fibromyalgia; **ICA,** independent component analysis; **NREM**, non-rapid eye movement stages; **NREMP**, non-REM parasomnia**; NT1**, narcolepsy type 1; **p,** p-value; **PCA**, principal component analysis; **Pha1-Pha7**, SO-phase patterns 1-7; **iRBD,** idiopathic/isolated rapid eye movement sleep behavior disorder; **REM,** rapid eye movement sleep stage **SO,** slow oscillations.

Table S17. Summary of Kruskal–Wallis test between five groups (PCA and ICA components of SO-phase histogram datasets; NREM + REM sleep stages together; components with significant differences only)

Channel	Component	Total N	H	DF	η2	p	α
F3	PCA 2	97	18.717	4	0.160	.001	**.096 ***
F3	ICA 2	97	18.612	4	0.159	.001	**.043 ***
F4	PCA 2	98	19.191	4	0.163	.001	**.096 ***
C3	PCA 1	97	17.969	4	0.152	.001	**.096 ***
C4	PCA 1	97	17.713	4	0.149	.001	**.096 ***
O1	PCA 5	98	16.873	4	0.138	.002	**.096 ***
O1	ICA 2	98	18.459	4	0.155	.001	**.043 ***
O2	PCA 5	95	17.239	4	0.147	.002	**.096 ***

Notes: * (in bold) denotes statistically significant differences for adjusted α-values after the Benjamini-Yekutieli procedure, * α ≤ .1. Please refer to **Table S1** for detailed information about the groups.

Abbreviations: **[C3, C4, F3, F4, O1, O2],** channels' codes in the standard 10-20% electroencephalography montage; **DF,** degree of freedom; **H**, Kruskal-Wallis H test statistics; **ICA**, independent component analysis; **NREM,** non-rapid eye movement stages; **p**, p-value; **PCA**, principal component analysis; **REM,** rapid eye movement sleep stage; **SO,** slow oscillations; **Total N**, number of subjects; **α,** α-value; **η2**, effect size.

Table S18. Summary of the two-tailed Mann–Whitney pairwise tests between Control group and each of patients' groups (NREM + REM sleep stages together, channel F3, PCA and ICA projections of SO-phase histogram datasets)

Patient group	Control				Patient group				Mann-Whitney statistics				
	Mean	SE	95% CI lower	95% CI upper	Mean	SE	95% CI lower	95% CI upper	U	z	η2	p	adj p
**PCA component 2**													
NREMP	1.929	4.610	-7.421	11.278	-3.478	6.069	-16.413	9.457	329.000	0.639	0.008	.529	1.000
NT1					19.485	7.547	3.399	35.571	189.000	-2.073	0.081	.039	.156
**iRBD**					**-19.108**	**5.015**	**-29.740**	**-8.476**	**468.000**	**2.859**	**0.151**	**.004**	**.018***
FM					-0.239	3.657	-8.387	7.909	197.000	-0.159	0.001	.883	1.000
**ICA component 2**													
NREMP	0.120	0.167	-0.219	0.458	0.136	0.202	-0.293	0.566	291.000	-0.097	0.000	.931	1.000
NT1					0.618	0.270	0.043	1.192	217.000	-1.531	0.044	.128	.513
**iRBD**					**-0.693**	**0.191**	**-1.099**	**-0.287**	**476.000**	**3.008**	**0.168**	**.003**	**.011***
FM					-0.429	0.226	-0.933	0.074	269.000	1.607	0.054	.111	.443

Notes: pairwise comparisons were performed only for PCA/ICA components for those with significant differences adjusted in the Kruskal–Wallis test between all 5 groups together (**Table S17**).

* (in bold) denotes statistically significant differences for adjusted p-values after the Bonferroni correction, $p \leq .05$. Please refer to **Table S1** for detailed information about the groups.

Abbreviations: **adj p,** Bonferroni-adjusted p-value**;** **F3,** channel's code in the standard 10-20% electroencephalography montage; **FM,** fibromyalgia; **ICA**, independent component analysis; **NREM,** non-rapid eye movement stages; **NREMP**, non-REM parasomnia; **NT1,** narcolepsy type 1; **p**, p-value; **PCA**, principal component analysis; **iRBD,** idiopathic/isolated rapid eye movement sleep behavior disorder; **REM,** rapid eye movement sleep stage; **SE,** standard error of mean; **SO,** slow oscillations; **U**, Mann–Whitney U test statistics; **z,** z-score; **η2,** effect size; **95% CI lower/upper**, 95% confidence interval lower/upper.

Table S19. Summary of the two-tailed Mann–Whitney pairwise tests between Control group and each of patients' groups (NREM + REM sleep stages together, channel F4, PCA and ICA projections of SO-phase histogram datasets)

Patient group	Control				Patient group				Mann-Whitney statistics				
	Mean	SE	95% CI lower	95% CI upper	Mean	SE	95% CI lower	95% CI upper	U	z	η2	p	adj p
**PCA component 2**													
NREMP	2.033	4.651	-7.392	11.457	-5.347	6.016	-18.170	7.476	350.000	0.871	0.014	.389	1.000
**NT**					**23.798**	**7.416**	**7.991**	**39.606**	**157.000**	**-2.785**	**0.144**	**.006**	**.022***
**iRBD**					**-18.191**	**6.153**	**-31.234**	**-5.147**	**462.000**	**2.532**	**0.117**	**.012**	**.047***
FM					-5.748	5.480	-17.958	6.463	252.000	1.030	0.022	.309	1.000

Notes: pairwise comparisons were performed only for PCA/ICA components for those with significant differences adjusted in the Kruskal–Wallis test between all 5 groups together (**Table S17**).

* (in bold) denotes statistically significant differences for adjusted p-values after the Bonferroni correction, p ≤ .05. Please refer to **Table S1** for detailed information about the groups.

Abbreviations: **adj p,** Bonferroni-adjusted p-value**; F4,** channel's code in the standard 10-20% electroencephalography montage; **FM,** fibromyalgia; **ICA**, independent component analysis; **NREM,** non-rapid eye movement stages; **NREMP**, non-REM parasomnia; **NT1,** narcolepsy type 1; **p**, p-value; **PCA**, principal component analysis; **iRBD,** idiopathic/isolated rapid eye movement sleep behavior disorder; **REM,** rapid eye movement sleep stage; **SE,** standard error of mean; **SO,** slow oscillations; **U**, Mann–Whitney U test statistics; **z,** z-score; **η2,** effect size; **95% CI lower/upper**, 95% confidence interval lower/upper.

Table S20. Summary of the two-tailed Mann–Whitney pairwise tests between Control group and each of patients' groups (NREM + REM sleep stages together, channel C3, PCA and ICA projections of SO-phase histogram datasets)

Patient group	Control				Patient group				Mann-Whitney statistics				
	Mean	SE	95% CI lower	95% CI upper	Mean	SE	95% CI lower	95% CI upper	U	z	η2	p	adj p
**PCA component 1**													
NREMP	3.456	7.271	-11.263	18.176	-11.555	8.817	-30.347	7.237	386.000	1.371	0.034	.173	.693
NT1					-27.044	9.886	-48.247	-5.842	417.000	2.404	0.107	.017	.067
iRBD					27.351	5.632	15.411	39.291	226.000	-1.880	0.063	.061	.245
FM					-0.921	7.270	-17.368	15.526	221.000	0.645	0.008	.527	1.000

Notes: pairwise comparisons were performed only for PCA/ICA components for those with significant differences adjusted in the Kruskal–Wallis test between all 5 groups together (**Table S17**).

Please refer to **Table S1** for detailed information about the groups.

Abbreviations: **adj p,** Bonferroni-adjusted p-value**; C3,** channel's code in the standard 10-20% electroencephalography montage; **FM,** fibromyalgia; **ICA**, independent component analysis; **NREM,** non-rapid eye movement stages; **NREMP**, non-REM parasomnia; **NT1,** narcolepsy type 1; **p**, p-value; **PCA**, principal component analysis; **iRBD,** idiopathic/isolated rapid eye movement sleep behavior disorder; **REM,** rapid eye movement sleep stage; **SE,** standard error of mean; **SO,** slow oscillations; **U**, Mann–Whitney U test statistics; **z,** z-score; **η2,** effect size; **95% CI lower/upper**, 95% confidence interval lower/upper.

Table S21. Summary of the two-tailed Mann–Whitney pairwise tests between Control group and each of patients' groups (NREM + REM sleep stages together, channel C4, PCA and ICA projections of SO-phase histogram datasets)

Patient group	Control				Patient group				Mann-Whitney statistics				
	Mean	SE	95% CI lower	95% CI upper	Mean	SE	95% CI lower	95% CI upper	U	z	η2	p	adj p
**PCA component 1**													
NREMP	-2.917	7.517	-18.135	12.300	14.969	6.980	-0.003	29.940	213.000	-1.535	0.044	.127	.508
NT					26.445	9.987	5.025	47.865	175.000	-2.269	0.095	.024	.095
iRBD					-27.487	5.994	-40.193	-14.780	420.000	1.577	0.044	.117	.467
FM					-3.650	4.425	-13.509	6.208	188.000	-0.621	0.008	.543	1.000

Notes: pairwise comparisons were performed only for PCA/ICA components for those with significant differences adjusted in the Kruskal–Wallis test between all 5 groups together (**Table S17**).

Please refer to **Table S1** for detailed information about the groups.

Abbreviations: **adj p,** Bonferroni-adjusted p-value**; C4,** channel's code in the standard 10-20% electroencephalography montage; **FM,** fibromyalgia; **ICA**, independent component analysis; **NREM,** non-rapid eye movement stages; **NREMP**, non-REM parasomnia; **NT1,** narcolepsy type 1; **p**, p-value; **PCA**, principal component analysis; **iRBD,** idiopathic/isolated rapid eye movement sleep behavior disorder; **REM,** rapid eye movement sleep stage; **SE,** standard error of mean; **SO,** slow oscillations; **U**, Mann–Whitney U test statistics; **z,** z-score; **η2,** effect size; **95% CI lower/upper**, 95% confidence interval lower/upper.

Table S22. Summary of the two-tailed Mann–Whitney pairwise tests between Control group and each of patients' groups (NREM + REM sleep stages together, channel O1, PCA and ICA projections of SO-phase histogram datasets)

Patient group	Control				Patient group				Mann-Whitney statistics				
	Mean	SE	95% CI lower	95% CI upper	Mean	SE	95% CI lower	95% CI upper	U	z	η2	p	adj p
**PCA component 5**													
**NREMP**	**1.723**	**2.811**	**-3.973**	**7.419**	**-12.370**	**3.580**	**-20.000**	**-4.740**	**456.000**	**2.879**	**0.154**	**.004**	**.016***
NT1					-7.334	4.701	-17.353	2.685	371.000	1.269	0.030	.208	.831
iRBD					7.762	4.196	-1.134	16.658	248.000	-1.366	0.034	.175	.699
FM					10.713	5.614	-1.796	23.221	152.000	-1.366	0.038	.176	.703
**ICA component 2**													
NREMP	0.156	0.175	-0.198	0.510	-0.156	0.146	-0.468	0.155	388.000	1.591	0.047	.114	.455
NT1					0.533	0.296	-0.099	1.164	236.000	-1.288	0.031	.201	.804
**iRBD**					**-0.721**	**0.163**	**-1.066**	**-0.376**	**519.000**	**3.570**	**0.232**	**3.700E-04**	**.001***
FM					0.030	0.253	-0.533	0.593	226.000	0.407	0.003	.693	1.000

Notes: pairwise comparisons were performed only for PCA/ICA components for those with significant differences adjusted in the Kruskal–Wallis test between all 5 groups together (**Table S17**).

* (in bold) denotes statistically significant differences for adjusted p-values after the Bonferroni correction, p ≤ .05. Please refer to **Table S1** for detailed information about the groups.

Abbreviations: **adj p,** Bonferroni-adjusted p-value**; FM,** fibromyalgia; **ICA**, independent component analysis; **NREM,** non-rapid eye movement stages; **NREMP**, non-REM parasomnia; **NT1,** narcolepsy type 1; **O1,** channel's code in the standard 10-20% electroencephalography montage; **p**, p-value; **PCA**, principal component analysis; **iRBD,** idiopathic/isolated rapid eye movement sleep behavior disorder; **REM,** rapid eye movement sleep stage; **SE,** standard error of mean; **SO,** slow oscillations; **U**, Mann–Whitney U test statistics; **z,** z-score; **η2,** effect size; **95% CI lower/upper**, 95% confidence interval lower/upper.

Table S23. Summary of the two-tailed Mann–Whitney pairwise tests between Control group and each of patients' groups (NREM + REM sleep stages together, channel O2, PCA and ICA projections of SO-phase histogram datasets)

Patient group	Control				Patient group				Mann-Whitney statistics				
	Mean	SE	95% CI lower	95% CI upper	Mean	SE	95% CI lower	95% CI upper	U	z	η2	p	adj p
**PCA component 5**													
**NREMP**	**-1.046**	**2.324**	**-5.755**	**3.664**	**-12.974**	**3.303**	**-20.110**	**-5.838**	**395.000**	**2.661**	**0.136**	**.008**	**.032***
NT1					-4.470	4.605	-14.286	5.345	334.000	0.568	0.006	.576	1.000
iRBD					8.472	4.037	-0.133	17.078	190.000	-2.160	0.086	.032	.126
FM					14.303	7.674	-2.796	31.403	127.000	-1.965	0.079	.051	.203

Notes: pairwise comparisons were performed only for PCA/ICA components for those with significant differences adjusted in the Kruskal–Wallis test between all 5 groups together (**Table S17**).

* (in bold) denotes statistically significant differences for adjusted p-values after the Bonferroni correction, $p \leq .05$. Please refer to **Table S1** for detailed information about the groups.

Abbreviations: **adj p,** Bonferroni-adjusted p-value**; FM,** fibromyalgia; **ICA**, independent component analysis; **NREM,** non-rapid eye movement stages; **NREMP**, non-REM parasomnia; **NT1,** narcolepsy type 1; **O2,** channel's code in the standard 10-20% electroencephalography montage; **p**, p-value; **PCA**, principal component analysis; **iRBD,** idiopathic/isolated rapid eye movement sleep behavior disorder; **REM,** rapid eye movement sleep stage; **SE,** standard error of mean; **SO,** slow oscillations; **U**, Mann–Whitney U test statistics; **z,** z-score; **η2,** effect size; **95% CI lower/upper**, 95% confidence interval lower/upper.

Table S24. Summary of Kruskal–Wallis test between five groups (PCA and ICA components of SO-phase histogram datasets; NREM sleep stages only; components with significant differences only)

Channel	Component	Total N	H	DF	η2	p	α
F3	ICA 1	97	15.769	4	0.128	.003	**.057***
F3	ICA 4	97	16.867	4	0.140	.002	**.047***
F4	ICA 3	98	25.069	4	0.227	4.873E-05	**.004***
C3	PCA 1	97	22.160	4	0.197	1.862E-04	**.032***
C3	ICA 3	97	18.442	4	0.157	.001	**.043***
C4	PCA 1	97	21.729	4	0.193	2.269E-04	**.032***
C4	ICA 3	97	16.719	4	0.138	.002	**.047***

Notes: * (in bold) denotes statistically significant differences for adjusted α-values after the Benjamini-Yekutieli procedure, * α ≤ .1. There were no significant differences for O1 and O2 channels (α>.1 in all the cases). Please refer to **Table S1** for detailed information about the groups.

Abbreviations: **[C3, C4, F3, F4, O1, O2],** channels' codes in the standard 10-20% electroencephalography montage; **DF,** degree of freedom; **H**, Kruskal-Wallis H test statistics; **ICA**, independent component analysis; **NREM,** non-rapid eye movement stages; **p**, p-value; **PCA**, principal component analysis; **REM,** rapid eye movement sleep stage; **SO,** slow oscillations; **Total N**, number of subjects; **α,** α-value; **η2**, effect size.

Table S25. Summary of the two-tailed Mann–Whitney pairwise tests between Control group and each of patients' groups (NREM sleep stages only, channel F3, PCA and ICA projections of SO-phase histogram datasets)

Patient group	Control				Patient group				Mann-Whitney statistics				
	**Mean**	**SE**	**95% CI lower**	**95% CI upper**	**Mean**	**SE**	**95% CI lower**	**95% CI upper**	**U**	**z**	**η2**	**p**	**adj p**
**ICA component 1**													
NREMP	0.216	0.157	-0.102	0.535	0.162	0.246	-0.362	0.686	322.000	0.504	0.005	.621	1.000
NT1					0.434	0.203	0.002	0.866	260.000	-0.697	0.009	.492	1.000
**iRBD**					**-0.523**	**0.204**	**-0.956**	**-0.090**	**453.000**	**2.579**	**0.123**	**.010**	**.041***
**FM**					**-0.786**	**0.329**	**-1.519**	**-0.053**	**309.000**	**2.588**	**0.140**	**.010**	**.040***
**ICA component 4**													
NREMP	0.032	0.189	-0.351	0.416	0.093	0.217	-0.370	0.556	259.000	-0.717	0.010	.479	1.000
NT1					0.574	0.230	0.083	1.065	200.000	-1.860	0.065	.064	.257
iRBD					-0.736	0.147	-1.048	-0.425	441.000	2.356	0.103	.019	.076
FM					0.059	0.207	-0.403	0.522	177.000	-0.650	0.009	.524	1.000

Notes: pairwise comparisons were performed only for PCA/ICA components for those with significant differences adjusted in the Kruskal–Wallis test between all 5 groups together (**Table S24**).

* (in bold) denotes statistically significant differences for adjusted p-values after the Bonferroni correction, p ≤ .05. Please refer to **Table S1** for detailed information about the groups.

Abbreviations: **adj p,** Bonferroni-adjusted p-value**; F3,** channel's code in the standard 10-20% electroencephalography montage; **FM,** fibromyalgia; **ICA**, independent component analysis; **NREM,** non-rapid eye movement stages; **NREMP**, non-REM parasomnia; **NT1,** narcolepsy type 1; **p**, p-value; **PCA**, principal component analysis; **iRBD,** idiopathic/isolated rapid eye movement sleep behavior disorder; **REM,** rapid eye movement sleep stage; **SE,** standard error of mean; **SO,** slow oscillations; **U**, Mann–Whitney U test statistics; **z,** z-score; **η2,** effect size; **95% CI lower/upper**, 95% confidence interval lower/upper.

Table S26. Summary of the two-tailed Mann–Whitney pairwise tests between Control group and each of patients' groups (NREM sleep stages only, channel F4, PCA and ICA projections of SO-phase histogram datasets)

Patient group	Control				Patient group				Mann-Whitney statistics				
	Mean	SE	95% CI lower	95% CI upper	Mean	SE	95% CI lower	95% CI upper	U	z	η2	p	adj p
**ICA component 3**													
NREMP	0.193	0.178	-0.168	0.554	-0.128	0.131	-0.407	0.150	344.000	0.758	0.011	.454	1.000
NT1					0.680	0.188	0.279	1.080	184.000	-2.273	0.096	.024	.094
**iRBD**					**-0.868**	**0.244**	**-1.386**	**-0.351**	**520.000**	**3.588**	**0.234**	**3.451E-04**	**.001***
FM					-0.127	0.148	-0.457	0.203	230.000	0.503	0.005	.623	1.000

Notes: pairwise comparisons were performed only for PCA/ICA components for those with significant differences adjusted in the Kruskal–Wallis test between all 5 groups together (**Table S24**).

* (in bold) denotes statistically significant differences for adjusted p-values after the Bonferroni correction, p ≤ .05. Please refer to **Table S1** for detailed information about the groups.

Abbreviations: **adj p,** Bonferroni-adjusted p-value**;** **F4,** channel's code in the standard 10-20% electroencephalography montage; **FM,** fibromyalgia; **ICA**, independent component analysis; **NREM,** non-rapid eye movement stages; **NREMP**, non-REM parasomnia; **NT1,** narcolepsy type 1; **p**, p-value; **PCA**, principal component analysis; **iRBD,** idiopathic/isolated rapid eye movement sleep behavior disorder; **REM,** rapid eye movement sleep stage; **SE,** standard error of mean; **SO,** slow oscillations; **U**, Mann–Whitney U test statistics; **z,** z-score; **η2,** effect size; **95% CI lower/upper**, 95% confidence interval lower/upper.

Table S27. Summary of the two-tailed Mann–Whitney pairwise tests between Control group and each of patients' groups (NREM sleep stages only, channel C3, PCA and ICA projections of SO-phase histogram datasets)

Patient group	Control				Patient group				Mann-Whitney statistics				
	Mean	SE	95% CI lower	95% CI upper	Mean	SE	95% CI lower	95% CI upper	U	z	η2	p	adj p
**PCA component 1**													
NREMP	4.035	7.158	-10.456	18.526	-14.232	7.204	-29.588	1.124	402.000	1.668	0.051	.097	.389
**NT1**					**-29.890**	**10.418**	**-52.235**	**-7.544**	**423.000**	**2.520**	**0.118**	**.012**	**.048***
iRBD					31.033	5.470	19.438	42.629	223.000	-1.933	0.067	.054	.217
FM					-0.887	6.673	-15.982	14.208	228.000	0.819	0.014	.420	1.000
**ICA component 3**													
NREMP	0.044	0.155	-0.270	0.358	-0.396	0.200	-0.821	0.029	396.000	1.557	0.044	.122	.487
NT1					-0.687	0.263	-1.250	-0.123	408.000	2.231	0.092	.026	.105
iRBD					0.692	0.175	0.321	1.063	197.000	-2.397	0.103	.017	.068
FM					0.316	0.335	-0.441	1.074	166.000	-0.719	0.011	.480	1.000

Notes: pairwise comparisons were performed only for PCA/ICA components for those with significant differences adjusted in the Kruskal–Wallis test between all 5 groups together (**Table S24**).

* (in bold) denotes statistically significant differences for adjusted p-values after the Bonferroni correction, p ≤ .05. Please refer to **Table S1** for detailed information about the groups.

Abbreviations: **adj p,** Bonferroni-adjusted p-value**; C3,** channel's code in the standard 10-20% electroencephalography montage; **FM,** fibromyalgia; **ICA**, independent component analysis; **NREM,** non-rapid eye movement stages; **NREMP**, non-REM parasomnia; **NT1,** narcolepsy type 1; **p**, p-value; **PCA**, principal component analysis; **iRBD,** idiopathic/isolated rapid eye movement sleep behavior disorder; **REM,** rapid eye movement sleep stage; **SE,** standard error of mean; **SO,** slow oscillations; **U**, Mann–Whitney U test statistics; **z,** z-score; **η2,** effect size; **95% CI lower/upper**, 95% confidence interval lower/upper.

Table S28. Summary of the two-tailed Mann–Whitney pairwise tests between Control group and each of patients' groups (NREM sleep stages only, channel C4, PCA and ICA projections of SO-phase histogram datasets)

Patient group	Control				Patient group				Mann-Whitney statistics				
	Mean	SE	95% CI lower	95% CI upper	Mean	SE	95% CI lower	95% CI upper	U	z	η2	p	adj p
**PCA component 1**													
NREMP	3.010	7.356	-11.883	17.902	-15.504	6.722	-29.921	-1.087	375.000	1.593	0.047	.113	.453
NT1					-28.712	9.886	-49.915	-7.509	418.000	2.424	0.109	.016	.063
iRBD					31.409	5.789	19.136	43.681	220.000	-1.987	0.070	.048	.192
FM					1.084	4.965	-9.980	12.147	242.000	0.644	0.008	.527	1.000
**ICA component 3**													
NREMP	-0.076	0.166	-0.413	0.260	0.425	0.233	-0.076	0.925	206.000	-1.671	0.052	.097	.387
NT1					0.619	0.262	0.058	1.181	184.000	-2.095	0.081	.037	.148
iRBD					-0.680	0.154	-1.006	-0.354	452.000	2.147	0.082	.032	.130
FM					-0.103	0.255	-0.671	0.464	217.000	0.059	0.000	.963	1.000

Notes: pairwise comparisons were performed only for PCA/ICA components for those with significant differences adjusted in the Kruskal–Wallis test between all 5 groups together (**Table S24**).

* (in bold) denotes statistically significant differences for adjusted p-values after the Bonferroni correction, p ≤ .05. Please refer to **Table S1** for detailed information about the groups.

Abbreviations: **adj p,** Bonferroni-adjusted p-value**; C4,** channel's code in the standard 10-20% electroencephalography montage; **FM,** fibromyalgia; **ICA**, independent component analysis; **NREM,** non-rapid eye movement stages; **NREMP**, non-REM parasomnia; **NT1,** narcolepsy type 1; **p**, p-value; **PCA**, principal component analysis; **iRBD,** idiopathic/isolated rapid eye movement sleep behavior disorder; **REM,** rapid eye movement sleep stage; **SE,** standard error of mean; **SO,** slow oscillations; **U**, Mann–Whitney U test statistics; **z,** z-score; **η2,** effect size; **95% CI lower/upper**, 95% confidence interval lower/upper.

Split-half reliability correlation matrices (SO-power histogram datasets, NREM + REM sleep stages together)

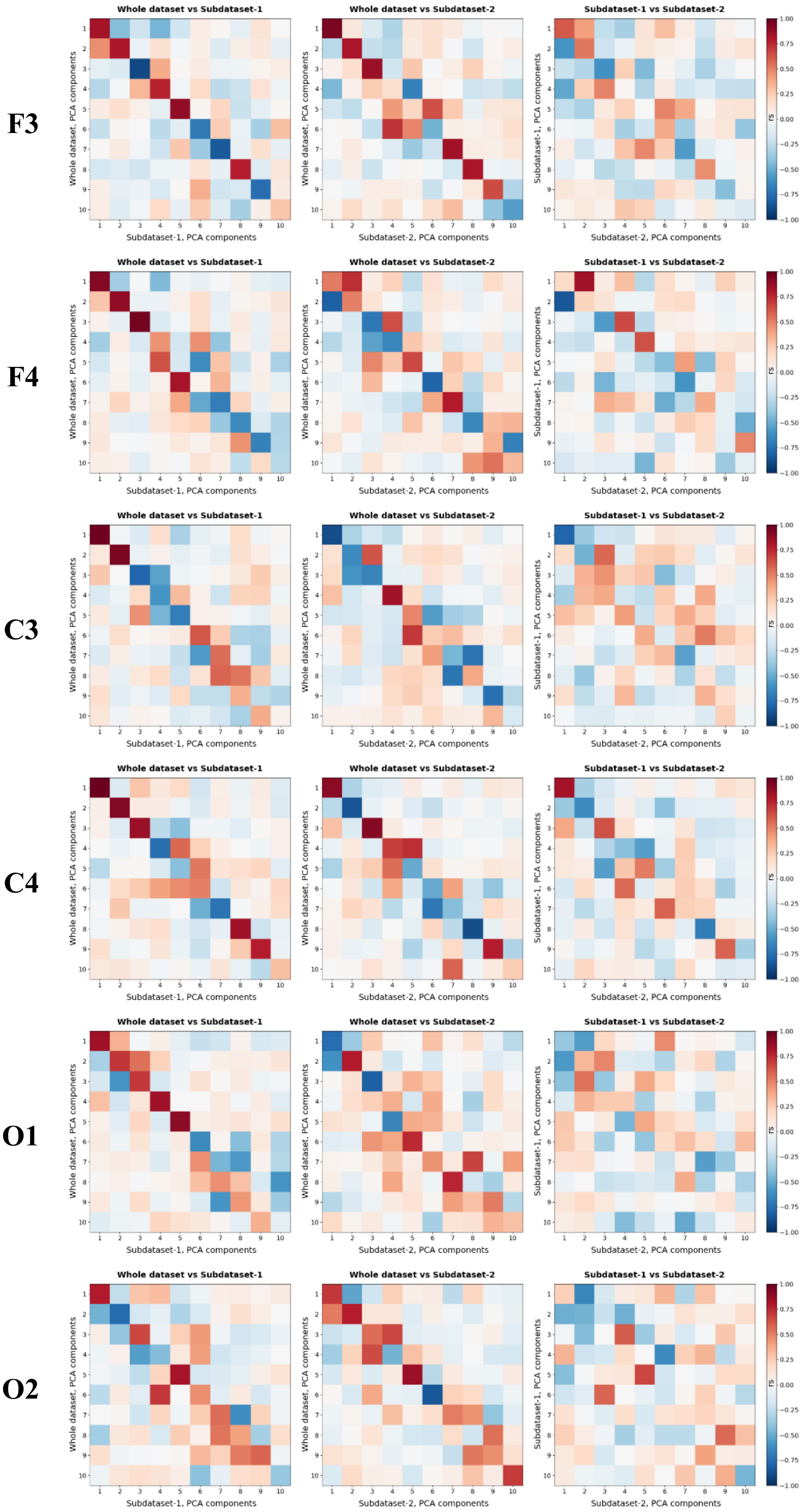

Fig. S12. Correlations between PCA component eigenvectors of the whole dataset and 2 subdatasets. Please see **Table S1** for detailed information about each group.

Abbreviations: **[C3, C4, F3, F4, O1, O2],** channel's code in the standard 10-20% electroencephalography montage; **PCA,** principal component analysis; **rs,** Spearman's correlation coefficient; **SO**, slow oscillations.

Split-half reliability correlation matrices (SO-phase histogram datasets, NREM + REM sleep stages together)

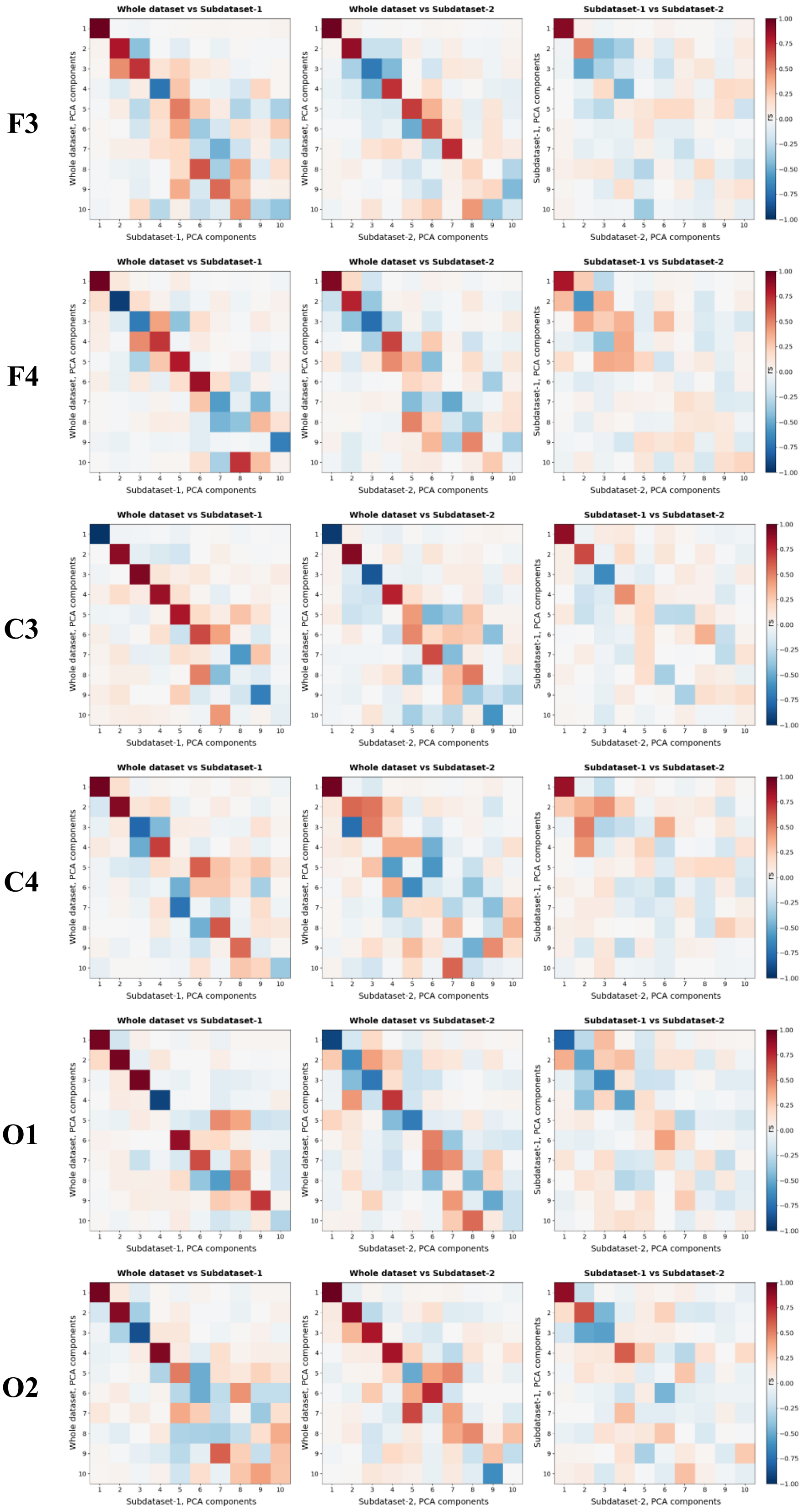

Fig. S13. Split-half reliability correlation matrices for SO-phase histograms (NREM+REM sleep). Correlations between PCA component eigenvectors of the whole dataset and 2 subdatasets. Please see **Table S1** for detailed information about each group.

Abbreviations: **[C3, C4, F3, F4, O1, O2],** channel's code in the standard 10-20% electroencephalography montage; **PCA,** principal component analysis; **rs,** Spearman's correlation coefficient; **SO**, slow oscillations.

ROC curves of the SO-power histogram datasets separability (NREM+REM stages together)

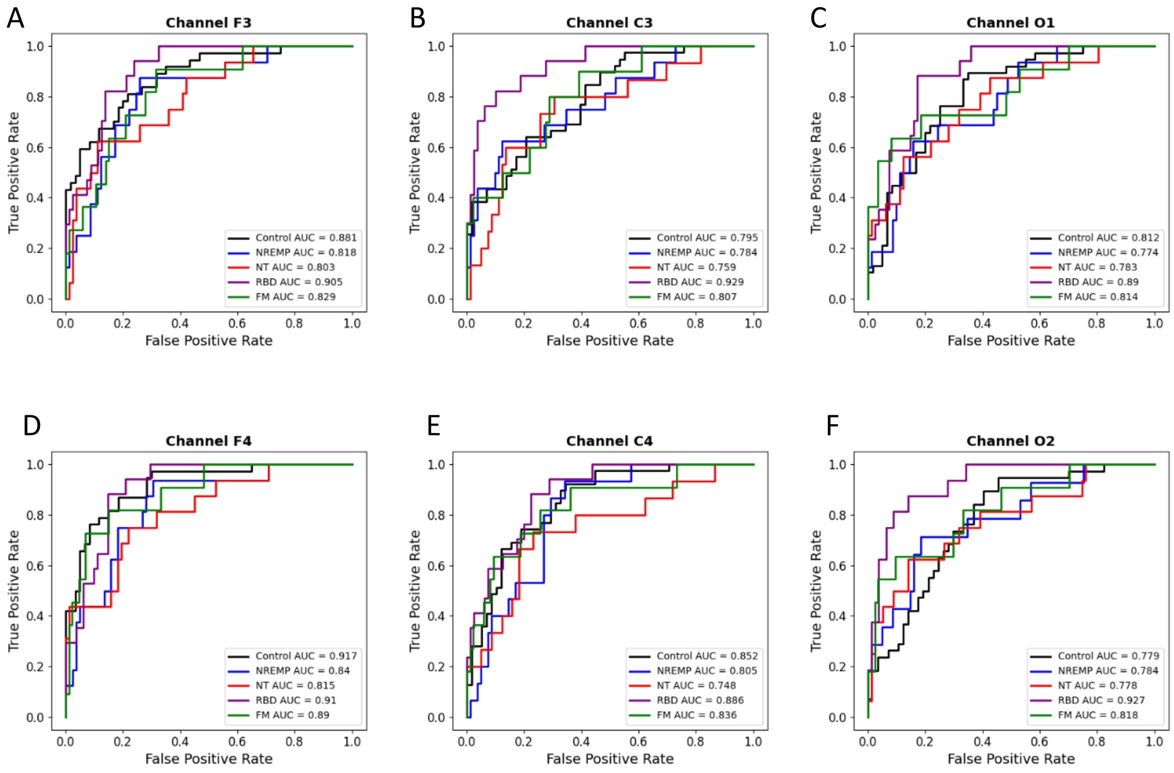

Figure S14. ROC curves illustrating group-level discrimination based on PCA projections of SO-power histograms (NREM+REM sleep stages combined).
Each curve reflects a binary logistic regression model trained to distinguish one group versus all others, using internal cross-validation. PCA was performed on the full dataset to preserve interpretability of the components. Detailed group information is provided in **Table S1**.

A, B, C, D, E, F: ROC curves for channels F3, C3, O1, F4, C4, O2, respectively.

Abbreviations: **AUC,** area under the ROC curve; [**C3, C4, F3, F4, O1, O2],** channel's code in the standard 10-20% electroencephalography montage; **FM,** fibromyalgia; **NREMP**, non-REM parasomnia**; NT1**, narcolepsy type 1; **PCA**, principal component analysis; **iRBD**, idiopathic REM behavior disorder; **REM,** rapid eye movement sleep stage; **ROC,** receiver operating characteristic curve; **SO,** slow oscillations.

ROC curves of the SO-phase histogram datasets separability (NREM + REM stages together)

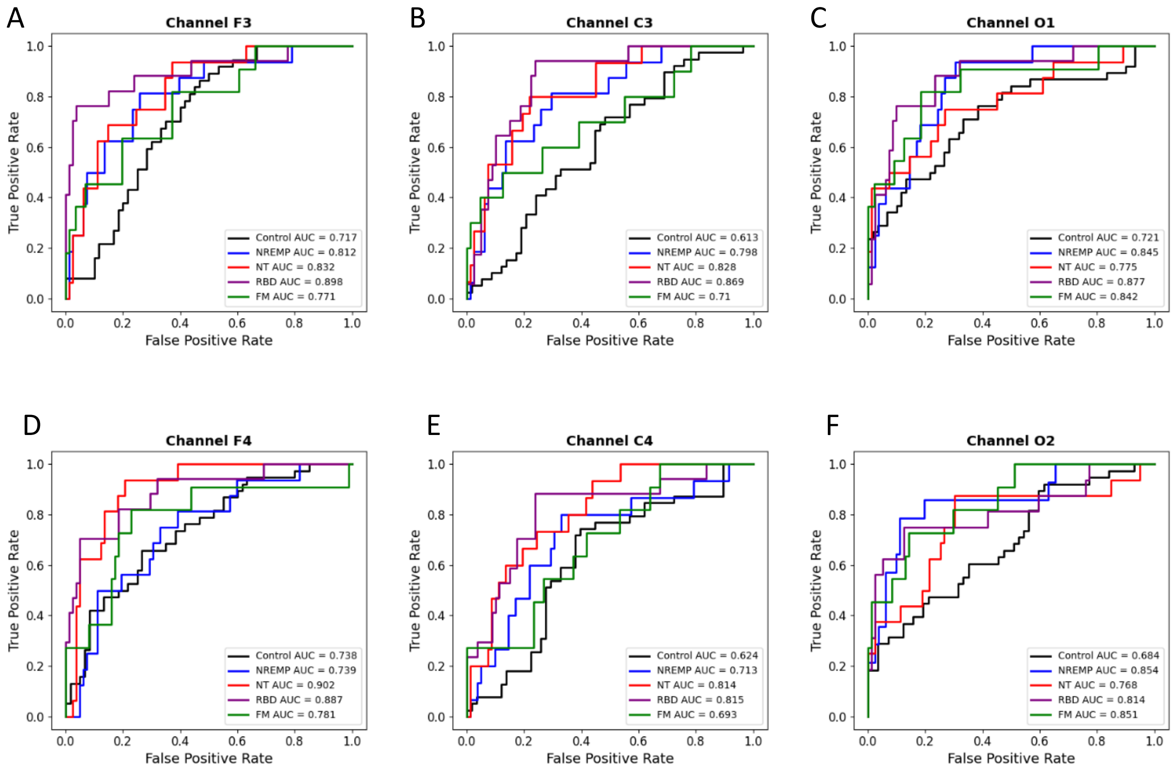

Figure S15. ROC curves illustrating group-level discrimination based on PCA projections of SO-phase histograms (NREM+REM sleep stages combined).

Each curve reflects a binary logistic regression model trained to distinguish one group versus all others, using internal cross-validation. PCA was applied to the full dataset to preserve component interpretability. Detailed group information is provided in **Table S1.**

A, B, C, D, E, F: ROC curves for channels F3, C3, O1, F4, C4, O2, respectively.

Abbreviations: **AUC,** area under the ROC curve; [**C3, C4, F3, F4, O1, O2],** channel's code in the standard 10-20% electroencephalography montage; **FM,** fibromyalgia; **NREMP**, non-REM parasomnia**; NT1**, narcolepsy type 1; **PCA**, principal component analysis; **iRBD**, idiopathic REM behavior disorder; **REM,** rapid eye movement sleep stage; **ROC,** receiver operating characteristic curve; **SO,** slow oscillations.

Permutations tests for SO-power histogram datasets separability
(NREM + REM sleep stages together)

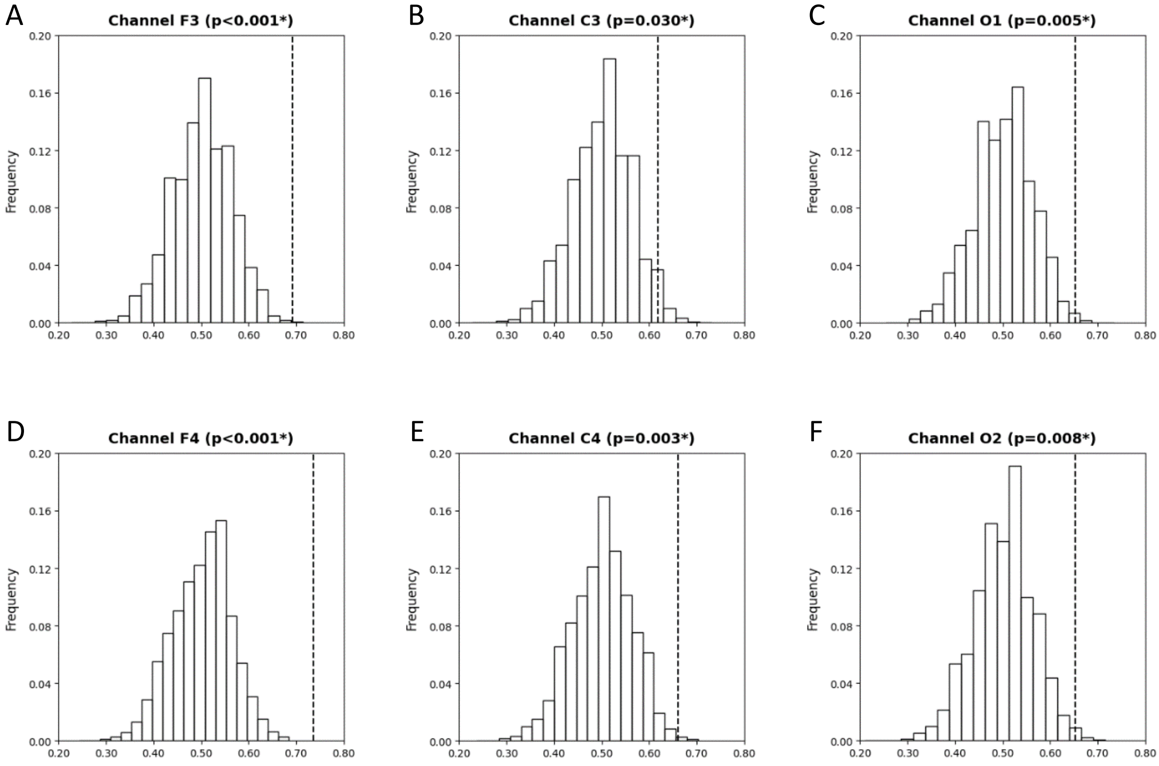

Figure S16. Permutation tests for logistic regression models trained in a fixed PCA feature space derived from SO-power histograms (NREM+REM stages combined).
Dashed lines indicate classifier performance (ROC-AUC) using true labels; histograms show the distribution of ROC-AUC values from 1,000 label shuffles. Detailed group information is provided in **Table S1**.

A, B, C, D, E, F: results of the permutations tests for channels F3, C3, O1, F4, C4, O2, respectively.
* $p \leq .05$. P-values for the channels F3 and F4 were 7.0E-4 and 1.0E-4, respectively.

Abbreviations: **[C3, C4, F3, F4, O1, O2],** channel's code in the standard 10-20% electroencephalography montage; **p**, p-value; **PCA**, principal component analysis; **SO,** slow oscillations.

Permutations tests for SO-phase histogram datasets separability
(NREM + REM sleep stages together)

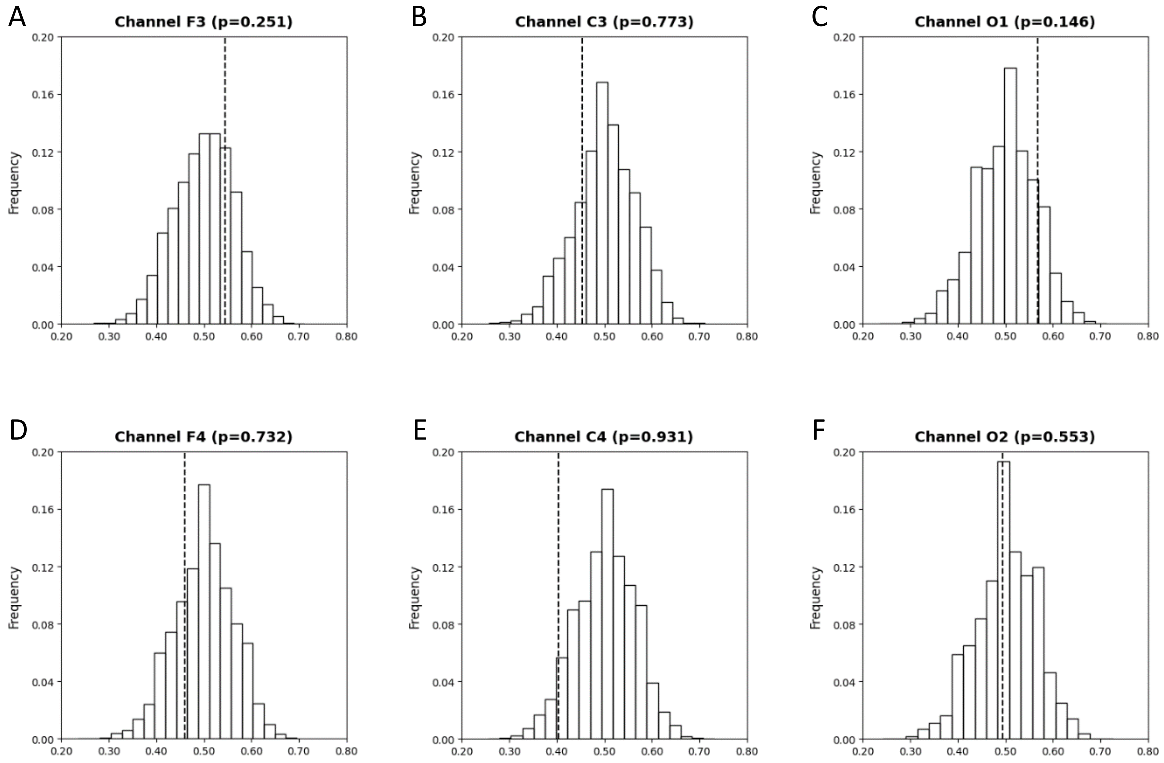

Figure S17. Permutation tests for logistic regression models trained in a fixed PCA feature space derived from SO-phase histograms. Dashed lines show ROC-AUC values on original labels. Detailed information about the groups is presented in **Table S1**.

A, B, C, D, E, F: results of the permutations tests for channels F3, C3, O1, F4, C4, O2, respectively.

Abbreviations: **[C3, C4, F3, F4, O1, O2],** channel's code in the standard 10-20% electroencephalography montage; **p**, p-value; **PCA**, principal component analysis; **SO,** slow oscillations.

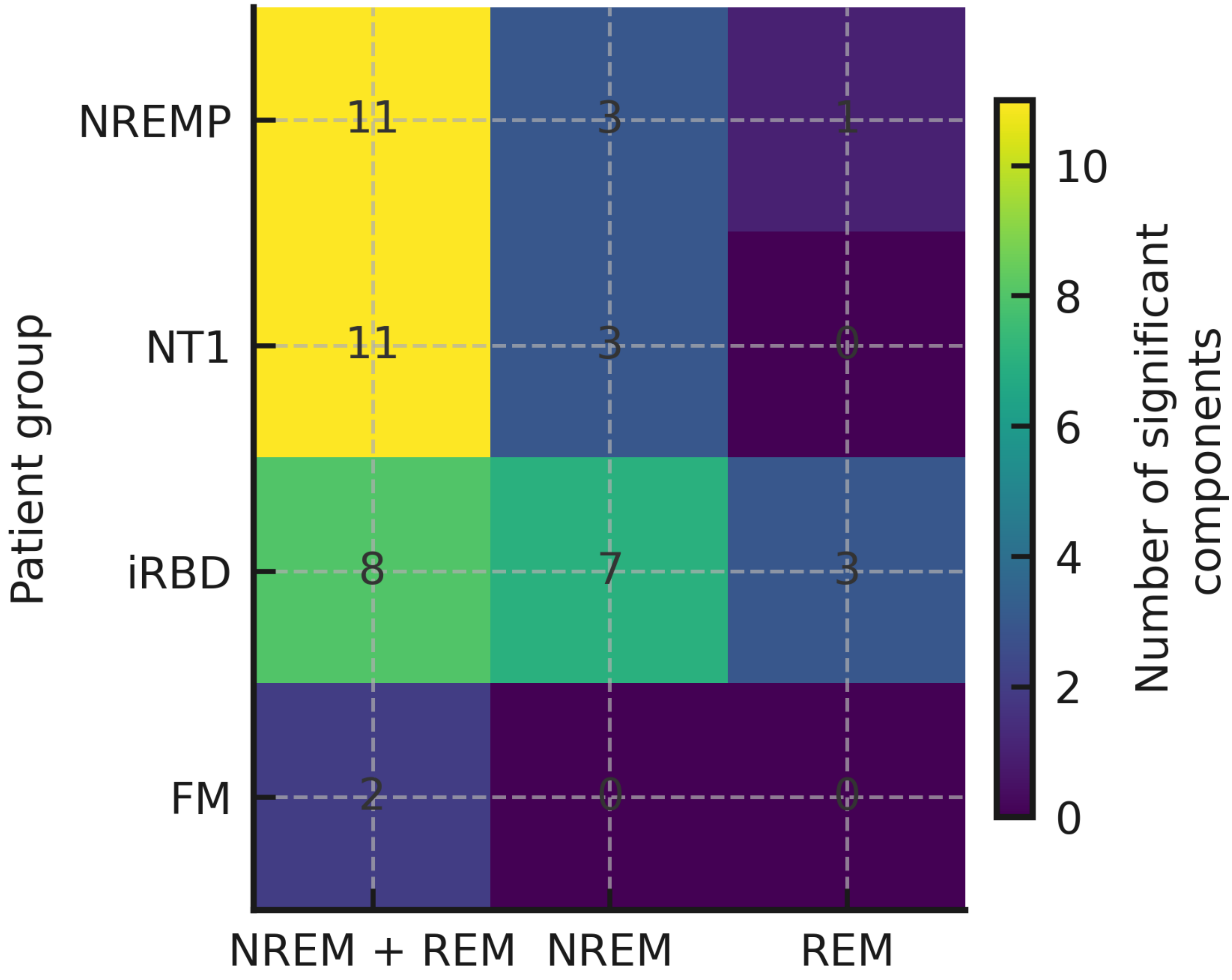

Figure S18. Summary of significant SO-power differences between controls and patient groups. Heatmap showing, for each patient group (rows) and sleep stage (columns), the number of SO-power components in which TF-peak features differed significantly from controls (Bonferroni-adjusted $p \leq 0.05$). Counts aggregate across channels (F3/F4, C3/C4, O1/O2) and across PCA and ICA components derived from SO-power histograms. NREM+REM sleep refers to analyses performed on combined NREM and REM sleep stages; NREM and REM sleep refer to stage-specific analyses. Numerical values in each cell indicate the exact number of significant components, and colour intensity encodes the same quantity. Component-wise statistics are reported in Supplementary Tables S3–S8 (NREM+REM), S10–S13 (NREM only), and S15–S16 (REM only).

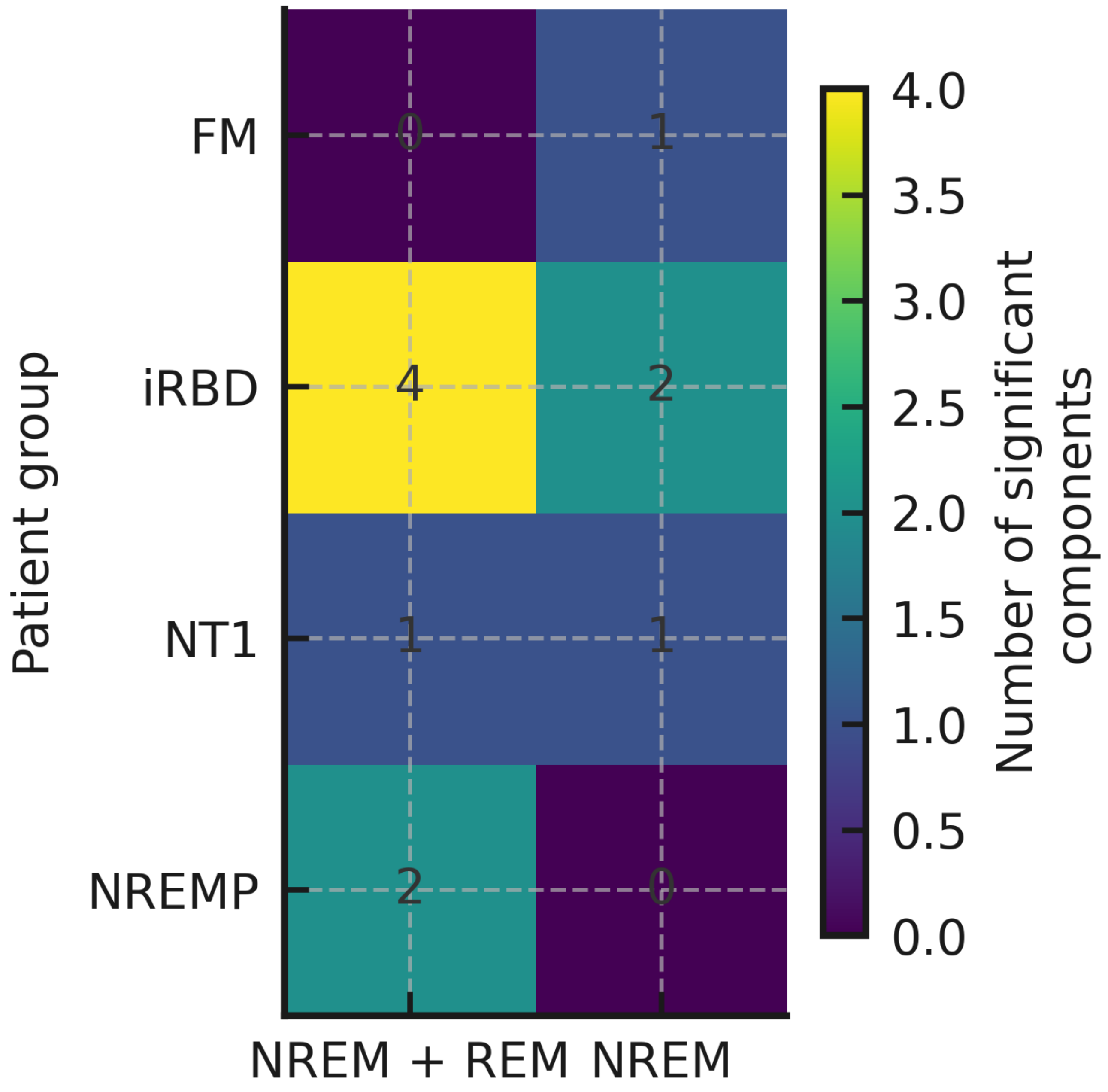

Figure S19. Summary of significant SO-phase differences between controls and patient groups. Heatmap showing, for each patient group (rows) and sleep stage (columns), the number of SO-phase components in which TF-peak features differed significantly from controls (Bonferroni-adjusted $p \leq 0.05$). Counts aggregate across channels and across PCA and ICA components derived from SO-phase histograms. As in Figure S18, NREM+REM sleep denotes analyses on combined stages, whereas NREM sleep refers to stage-specific analyses; no REM-only sleep SO-phase components reached significance after correction. Numerical values in each cell indicate the exact number of significant components, and colour intensity reflects the same quantity. Component-wise statistics are reported in Supplementary Tables S18–S23 (NREM+REM) and S25–S28 (NREM only).
